# Supplementary material for: Towards a better characterisation of deep-diving whales’ distributions by using prey distribution model outputs?
Source: PLoS One. 2021 Aug 4;16(8):e0255667. doi: 10.1371/journal.pone.0255667 (PMC8336804; doi:10.1371/journal.pone.0255667)
Supplement: S1 Appendix — Total effort represents the total length of transects of each survey (without removing the transects with a Beaufort sea-state > 4). NE-ATL: Northeast Atlantic Ocean; NW-ATL: Northwest Atlantic Ocean. (PDF) [file pone.0255667.s001.pdf]

S1 Appendix. Details of surveys used in the analyses. Total effort represents the total length of transects of each survey (without removing the transects with a Beaufort sea-state > 4). NE-ATL: Northeast Atlantic Ocean; NW-ATL: Northwest Atlantic Ocean.

Survey names (Fig. 1)	Organisation	Platform type	Surveyed years	Surveyed regions and sectors	Total effort (km)	References
AMBAR	AMBAR	Ship	2004-2005	South east of the Bay of Biscay; NE-ATL	5,073	Vázquez et al., 2003; 2004 ; 2005
ATLANCET	PELAGIS	Plane	2002	Bay of Biscay; NE-ATL	3,815	Certain et al., 2008
BMMRO	BMMRO	Ship	2000-2005	Bahamas; TROPICS	3,685	Shick et al., 2011
CODA	SMRU	Ship	2007	Northeast Atlantic; NE-ATL	9,645	Rogan et al., 2017
JNCC-ESAS	Joint Nature Conservation Committee - European Seabirds at Sea data providers	Ship	1998-2000, 2002, 2005, 2008-2010	Northeast Atlantic; NE-ATL	292,363	Reid et al., 2003
INDEMARES	CEMMA	Ship	2009-2011	West of the Spanish coasts; NE-ATL	6,488	López & Martínez-Cedeira, 2012
JUVENA	AZTI	Ship	2012-2015	Bay of Biscay; NE-ATL	8,862	Boyra et al., 2013
NEFSC	NEFSC	Plane and ship	1998-1999, 2010-2014	Continental shelf of the USA; NW-ATL	556,963	Roberts et al., 2016
PELACUS	Instituto Español de Oceanografía (IEO)	Ship	2007-2012	North and NW Spanish shelf waters; NE-ATL	9,585	Santos et al., 2013
PELGAS	PELAGIS	Ship	2007-2013	Bay of Biscay; NE-ATL	34,997	Certain et al., 2008
REMMOA	PELAGIS	Plane	2008	French West Indies and Guyana; TROPICS	15,356	Ridoux et al., 2010; Mannocci et al., 2013

SAMM	PELAGIS	Plane	2011-2012	Bay of Biscay, English Channel and western Mediterranean Sea; NE-ATL and MED	98,799	Laran et al., 2017; Lambert et al., 2017
SCANS II	SMRU	Ship	2005	Northeast Atlantic; NE-ATL	19,827	Rogan et al., 2017
THUNNUS	CEMMA	Ship	2007-2010	Bay of Biscay; NE-ATL	11,693	Martínez-Cedeira & López, 2010

References

Boyra, G., Martinez, U., Cotano, U., Santos, M., Irigoien, X. & Uriarte, A. (2013). Acoustic surveys for juvenile anchovy in the Bay of Biscay: abundance estimate as an indicator of the next year's recruitment and spatial distribution patterns. ICES Journal of Marine Science, 70:1354–1368.

Certain, G., Ridoux, V., Van Canneyt, O., & Bretagnolle, V. (2008). Delphinid spatial distribution and abundance estimates over the shelf of the Bay of Biscay. ICES Journal of Marine Science, 65(4): 656-666.

Laran, S., Authier, M., Blanck, A., Doremus, G., Falchetto, H., Monestiez, P., ... & Ridoux, V. (2017). Seasonal distribution and abundance of cetaceans within French waters-Part II: The Bay of Biscay and the English Channel. Deep Sea Research Part II: Topical Studies in Oceanography, 141: 31-40.

López, A., & Martínez-Cedeira, J. (2012). Final report of the project "LIFE 07/NAT/E/000732 INDEMARES". Unpublished technical report. CEMMA. 305 pp.

Mannocci, L., Monestiez, P., Bolaños-Jiménez, J., Dorémus, G., Jeremie, S., Laran, S., ... & Ridoux, V. (2013). Megavertebrate communities from two contrasting ecosystems in the western tropical Atlantic. Journal of Marine Systems 111: 208-222.

Martínez-Cedeira, J., & López, A. (2010). Final Report Thunnus 2007-2010 Surveys. Unpublished technical report. CEMMA. 87 pp.

Reid, J.B., Evans, P.G., & Northridge, S.P. (2003). Atlas of cetacean distribution in north-west European waters. Joint Nature Conservation Committee.

Ridoux, V, Certain, G., Doremus, G., Laran, S., van Canneyt, O., & Watremez, P. (2010). Mapping diversity and relative density of cetaceans and other pelagic megafauna across the tropics: general design and progress of the REMMOA aerial surveys conducted in the French EEZ and adjacent waters (Vol. 14). SC/62.

Roberts, J.J., Best, B.D., Dunn, D.C., Treml, E.A., & Halpin, P.N. (2010). Marine Geospatial Ecology Tools: An integrated framework for ecological geoprocessing with ArcGIS, Python, R, MATLAB, and C++. Environ. Model. Softw. 25: 1197–1207.

Rogan, E., Cañadas, A., Macleod, K., Santos, M.B., Mikkelsen, B., Uriarte, A., … Hammond, P.S., (2017). Distribution abundance and habitat use of deep diving cetaceans in the North-East Atlantic. Deep Sea Research Part II: Topical Studies in Oceanography, 141: 8-19.

Santos, M.B., González-Quirós, R., Riveiro, I., Iglesias, M. Louzao, M., & Pierce, G.J. (2013). Characterization of the pelagic fish community of the North Western and Northern Spanish shelf waters. Journal of Fish Biology, 83(4): 716-738.

Schick, R.S., Halpin, P.N., Read, A.J., Urban, D.L., Best, B.D., Good, C.P., ... Hyrenbach, K.D. (2011). Community structure in pelagic marine mammals at large spatial scales. Marine Ecology Progress Series, 434: 165-181.

Vázquez, A., Ruiz, L., Maestre, Z., Ruiz-Gondra, J., Ruiz-Guijarro, J., Benedicto, L., … Goenaga I. (2003). Land based sightings from the Basque Country coast (northeast Spain). 17th Annual Conference of the European Cetacean Society, Las Palmas de Gran Canaria, Canary Islands (Spain).

Vázquez, J.A., Cermeño, P., Williams, A., Martin, C., Lazkano, O., Ruiz, L., … Guzman, I. (2004). Identifying areas of special interest for Cuvier's beaked whale (*Ziphius cavirostris*) in the southern part of the Bay of Biscay. 18th Annual Conference of the European Cetacean Society, Kolmårdon, Sweden.

Vázquez, J.A., Guzmán, I. Lazkano, O., & Olondo, M. (2005). Encounter rates of small cetaceans, pilot whales and Ziphiidae in coastal waters of Basque Country (Southern Bay of Biscay). 19th Annual Conference of the European Cetacean Society, La Rochelle, France.
