# Supplementary material for: Towards a better characterisation of deep-diving whales’ distributions by using prey distribution model outputs?
Source: PLoS One. 2021 Aug 4;16(8):e0255667. doi: 10.1371/journal.pone.0255667 (PMC8336804; doi:10.1371/journal.pone.0255667)
Supplement: S2 Appendix — Base map from https://www.gebco.net/. (PDF) [file pone.0255667.s002.pdf]

S2 Appendix. Average conditions of the static, oceanographic and SEAPODYM variables over the entire period (from 1998 to 2015). Base map from https://www.gebco.net/.

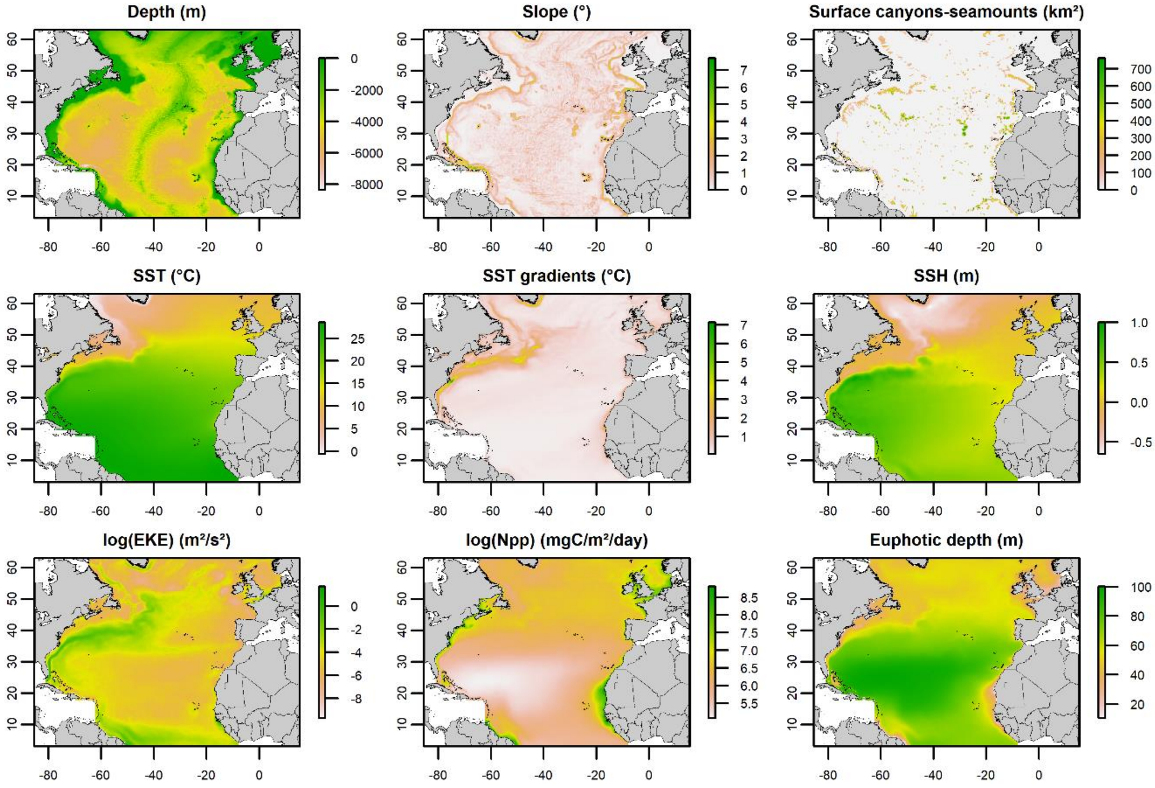

S2.1 Fig. Average conditions of the static and oceanographic variables over the entire period (from 1998 to 2015). SST: sea surface temperature; SSH: sea surface height; EKE: eddy kinetic energy; Npp: net primary production. Base map from https://www.gebco.net/.

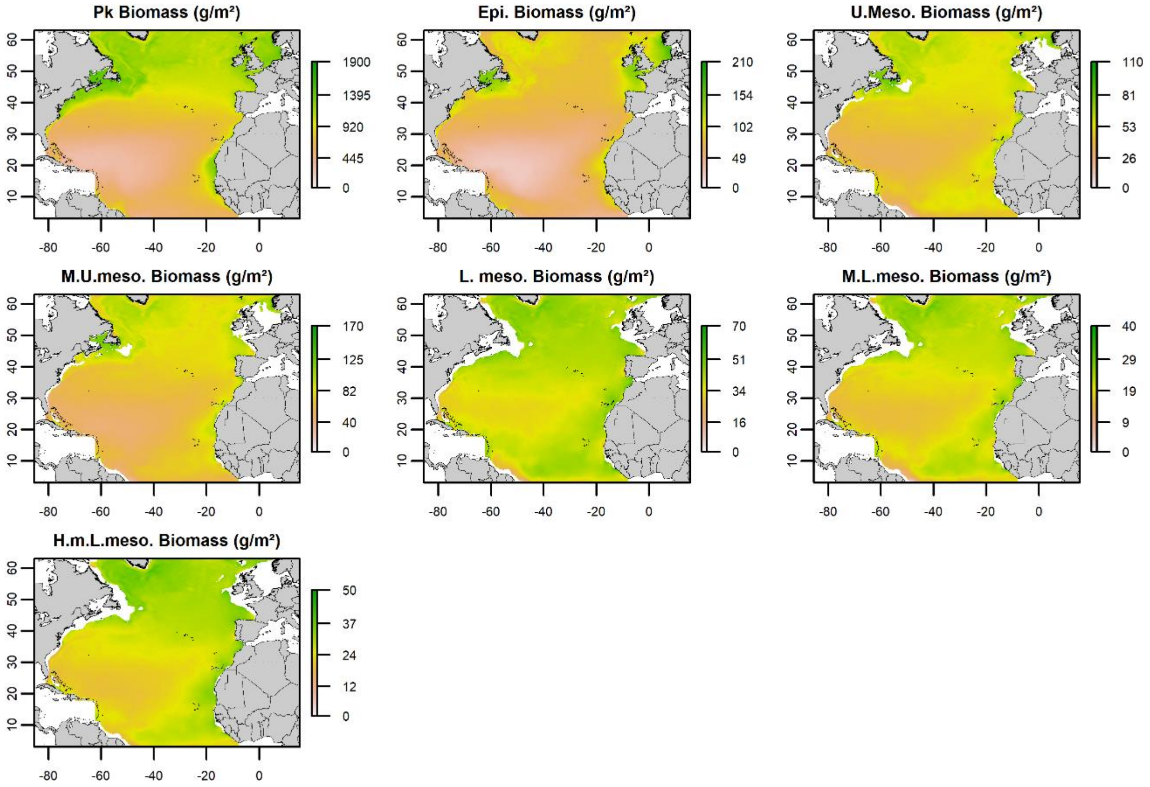

S2.2 Fig. Average conditions of the biomass SEAPDOYM variables over the entire period (from 1998 to 2015). Pk: zooplankton; Epi: epipelagic; U.Meso: upper mesopelagic; M.U.meso: migrant upper

mesopelagic; L.meson: lower mesopelagic; M.L.meso: migrant lower mesopelagic; H.m.L.meso: highly migrant lower mesopelagic. Base map from https://www.gebco.net/.

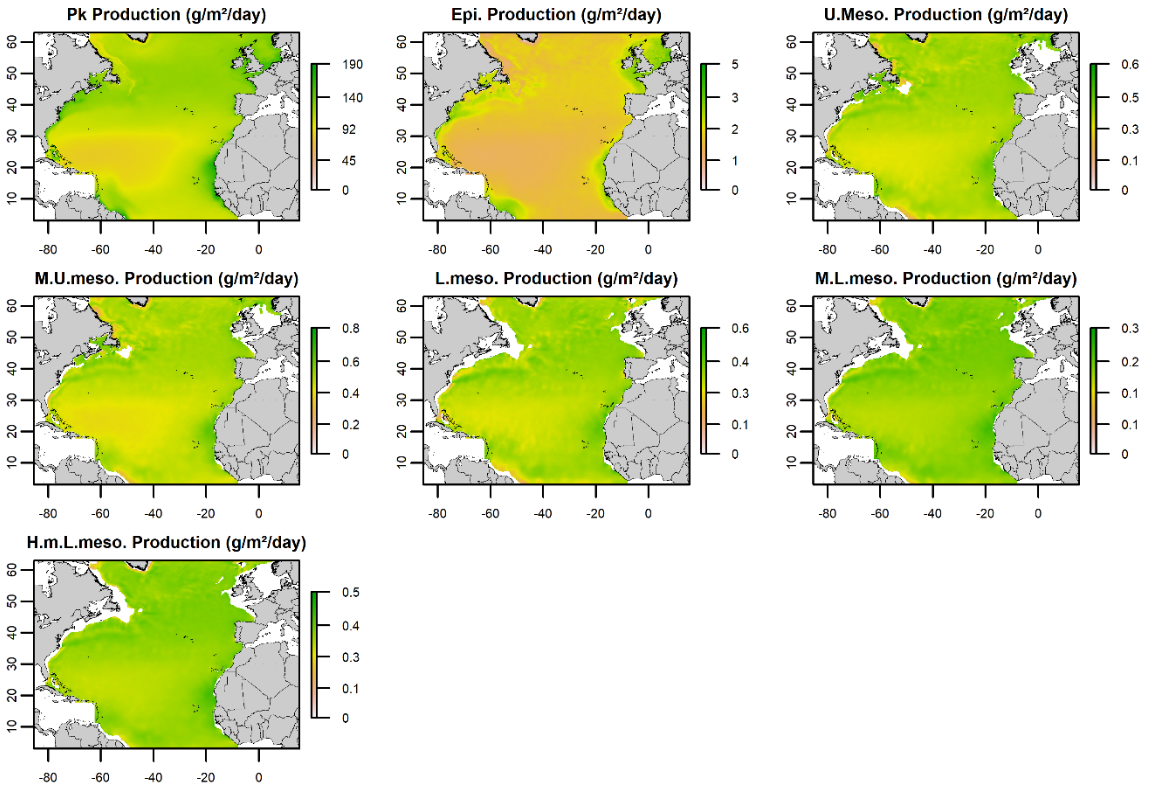

S2.3 Fig. Average conditions of the production SEAPDOYM variables over the entire period (from 1998 to 2015). Pk: zooplankton; Epi: epipelagic; U.Meso: upper mesopelagic; M.U.meso: migrant upper mesopelagic; L.meson: lower mesopelagic; M.L.meso: migrant lower mesopelagic; H.m.L.meso: highly migrant lower mesopelagic. Base map from https://www.gebco.net/.
