# Supplementary material for: Towards a better characterisation of deep-diving whales’ distributions by using prey distribution model outputs?
Source: PLoS One. 2021 Aug 4;16(8):e0255667. doi: 10.1371/journal.pone.0255667 (PMC8336804; doi:10.1371/journal.pone.0255667)
Supplement: S3 Appendix — (PDF) [file pone.0255667.s003.pdf]

S3 Appendix. Correlations between environmental and SEAPODYM variables and model outputs for beaked and sperm whales.

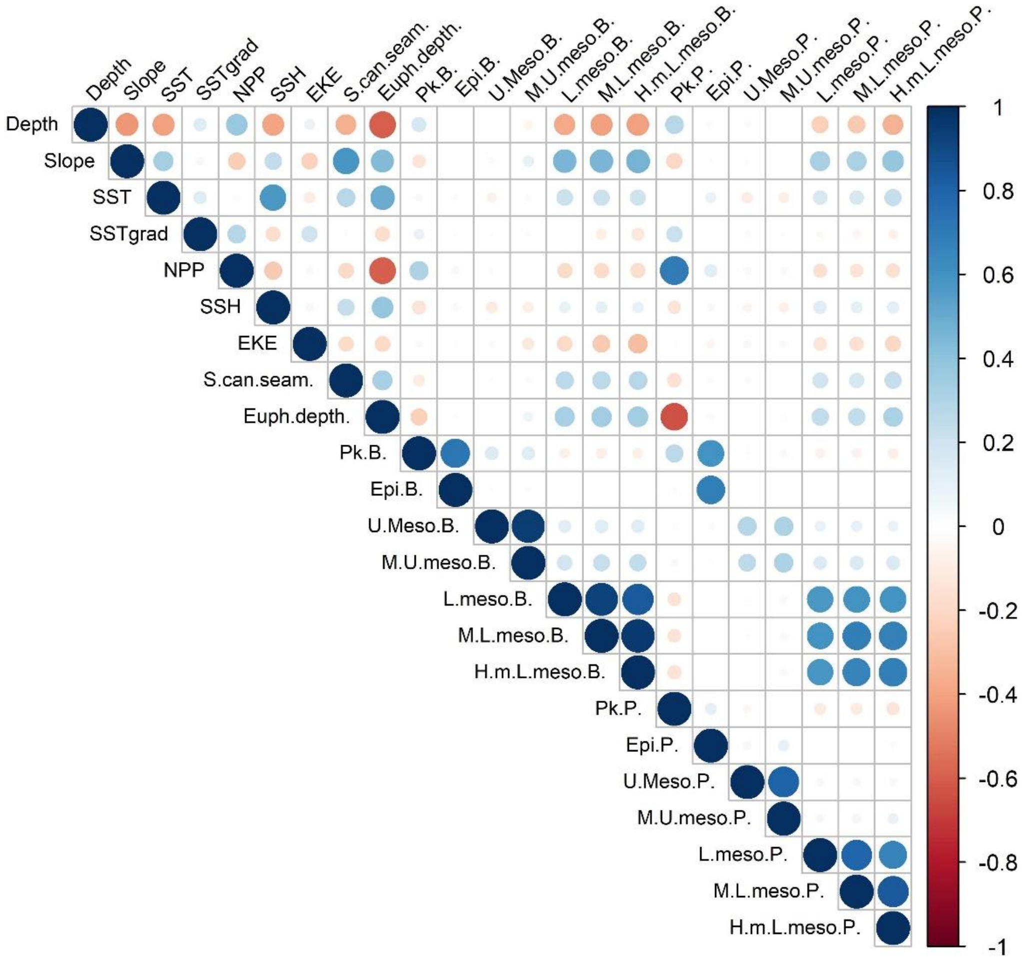

S3.1 Fig. Correlation matrix calculated between environmental and SEAPODYM variables. We used Pearson's correlation coefficients to calculate the matrix and the 'corrplot' R-package (Wei & Simko, 2017) to plot the matrix. Positive correlations are displayed in blue and negative correlations in red. The intensity of the colour and the size of the circles are proportional to the correlation coefficients. To the right of the correlogram, the colour legend shows the correlation coefficients and the corresponding colours. SST: sea surface height; SSTgrad: gradient of sea surface temperature; NPP: net primary production; SSH: sea surface height; EKE: eddy kinetic energy; S.can.seam: surface of the cell occupied by canyons and seamounts; Euph.depth: Euphotic depth; B.: biomass; P.: production; M.: migrant; H.m.: highly migrant; U: upper; L: lower; Epi.: epipelagic; Meso.: mesopelagic; Pk: zooplankton.

S3.1 Table. Five best ENVIRONMENTAL, SEAPODYM and COMBINED beaked whale models ranked by Akaike criterion (AIC). We only show the five best models to limit the size of the appendix. D*: explained deviance in %; ΔAIC: relative difference between the best model (which has a ΔAIC of zero) and each other model in the set; BW: beaked whale; s(): smooth function; k: sets the upper limit on the degrees of freedom; SSTgrad: gradients of sea surface temperature; S.can.seam: surface of the cell occupied by canyons and seamounts; SST: sea surface temperature; NPP: net primary production; SSH: sea surface height; Euph. depth: euphotic depth; B.: biomass; P.: production; M.: migrant; H.m.: highly migrant; u: upper; L: lower; Epi.: epipelagic; Meso.: mesopelagic.

**ENVIRONMENTAL model**	**D***	**AIC**	**ΔAIC**
BW~1 + s(Depth,k = 4) + s(Slope,k = 4) + s(SST,k = 4) + s(SSTgrad,k = 4)	39.8	135,451	0.0
BW~1 + s(Depth,k = 4) + s(Slope,k = 4) + s(SSTgrad,k = 4) + s(S.can.seam,k = 4)	39.7	135,453	1.9
BW~1 + s(Depth,k = 4) + s(Slope,k = 4) + s(SSTgrad,k = 4) + s(SSH,k = 4)	39.4	135,456	5.0
BW~1 + s(Depth,k = 4) + s(Slope,k = 4) + s(SSTgrad,k = 4) + s(NPP,k = 4)	40.4	135,460	8.9
BW~1 + s(Depth,k = 4) + s(SST,k = 4) + s(SSTgrad,k = 4) + s(S.can.seam,k = 4)	39.1	135,461	10.5
**SEAPODYM model**			
BW~1 + s(Euph.depth,k = 4) + s(L.meso.B,k = 4) + s(M.U.meso.B,k = 4) + s(M.L.meso.P,k = 4)	27.5	135,786	0.0
BW~1 + s(Euph.depth,k = 4) + s(L.meso.B,k = 4) + s(Epi.B,k = 4) + s(M.L.meso.P,k = 4)	27.6	135,790	4.2
BW~1 + s(Euph.depth,k = 4) + s(L.meso.B,k = 4) + s(M.L.meso.P,k = 4) + s(Epi.P,k = 4)	27.1	135,794	8.0
BW~1 + s(Euph.depth,k = 4) + s(L.meso.B,k = 4) + s(U.meso.B,k = 4) + s(M.L.meso.P,k = 4)	26.9	135,797	10.6
BW~1 + s(Euph.depth,k = 4) + s(L.meso.B,k = 4) + s(M.L.meso.P,k = 4) + s(U.meso.P,k = 4)	27.1	135,799	12.0
**COMBINED model**			
BW~1 + s(Depth,k = 4) + s(Slope,k = 4) + s(SST,k = 4) + s(SSTgrad,k = 4)	39.8	135,451	0.0
BW~1 + s(Depth,k = 4) + s(SST,k = 4) + s(SSTgrad,k = 4) + s(S.can.seam,k = 4)	39.1	135,461	9.8
BW~1 + s(Depth,k = 4) + s(Slope,k = 4) + s(SSTgrad,k = 4)	39.0	135,465	13.9
BW~1 + s(Depth,k = 4) + s(Slope,k = 4) + s(SSTgrad,k = 4) + s(L.meso.B.,k = 4)	39.2	135,466	14.5
BW~1 + s(Depth,k = 4) + s(Slope,k = 4) + s(SSTgrad,k = 4) + s(M.L.meso.P.,k = 4)	39.0	135,467	15.6

S3.2 Table. Five best ENVIRONMENTAL, SEAPODYM and COMBINED sperm whale models ranked by Akaike criterion (AIC). We only show the five best models to limit the size of the appendix. D*: explained deviance in %; ΔAIC: relative difference between the best model (which has a ΔAIC of zero) and each other model in the set; BW: beaked whale; s(): smooth function; k: sets the upper limit on the degrees of freedom; SSTgrad: gradients of sea surface temperature; SSH: sea surface height; EKE: eddy kinetic energy; SST: sea surface temperature; Euph. depth: euphotic depth; B.: biomass; P.: production; M.: migrant; H.m.: higly migrant; U: upper; L: lower; Epi.: epipelagic; Meso.: mesopelagic.

**ENVIRONMENTAL model**	**D***	**AIC**	**ΔAIC**
SW~1 + s(Depth,k = 4) + s(Slope,k = 4) + s(SSTgrad,k = 4) + s(SSH,k = 4)	31.9	133,843	0.0
SW~1 + s(Depth,k = 4) + s(SSTgrad,k = 4) + s(EKE,k = 4) + s(SSH,k = 4)	31.2	133,844	0.6
SW~1 + s(Depth,k = 4) + s(SSTgrad,k = 4) + s(SSH,k = 4)	31.0	133,845	1.7
SW~1 + s(Depth,k = 4) + s(SSTgrad,k = 4) + s(SSH,k = 4) + s(SST,k = 4)	31.5	133,846	2.4
SW~1 + s(Depth,k = 4) + s(Slope,k = 4) + s(SSTgrad,k = 4) + s(EKE,k = 4)	31.2	133,846	2.8
**SEAPODYM model**			
SW~1 + s(Euph.depth,k = 4) + s(L.meso.B,k = 4) + s(Epi.B,k = 4) + s(Epi.P,k = 4)	19.3	134,029	0.0
SW~1 + s(Euph.depth,k = 4) + s(Epi.B,k = 4) + s(M.L.meso.B,k = 4) + s(Epi.P,k = 4)	19.2	134,044	15.6
SW~1 + s(Euph.depth,k = 4) + s(Epi.B,k = 4) + s(H.m.L.meso.P,k = 4) + s(Epi.P,k = 4)	17.8	134,049	20.4
SW~1 + s(Euph.depth,k = 4) + s(Epi.B,k = 4) + s(H.m.L.meso.B,k = 4) + s(Epi.P,k = 4)	18.5	134,052	22.9
SW~1 + s(Euph.depth,k = 4) + s(U.meso.P,k = 4) + s(Epi.B,k = 4) + s(Epi.P,k = 4)	16.7	134,053	23.8
**COMBINED model**			
SW~1 + s(Depth,k = 4) + s(SSTgrad,k = 4) + s(SSH,k = 4) + s(Epi.P,k = 4)	31.1	133,841	0.0
SW~1 + s(Depth,k = 4) + s(Slope,k = 4) + s(SSTgrad,k = 4) + s(SSH,k = 4)	31.9	133,843	2.6
SW~1 + s(Depth,k = 4) + s(SSTgrad,k = 4) + s(EKE,k = 4) + s(SSH,k = 4)	31.2	133,844	3.1
SW~1 + s(Depth,k = 4) + s(SSTgrad,k = 4) + s(SSH,k = 4) + s(Euph.depth,k = 4)	31.3	133,844	3.3
SW~1 + s(Depth,k = 4) + s(SSTgrad,k = 4) + s(SSH,k = 4)	31.0	133,845	4.3

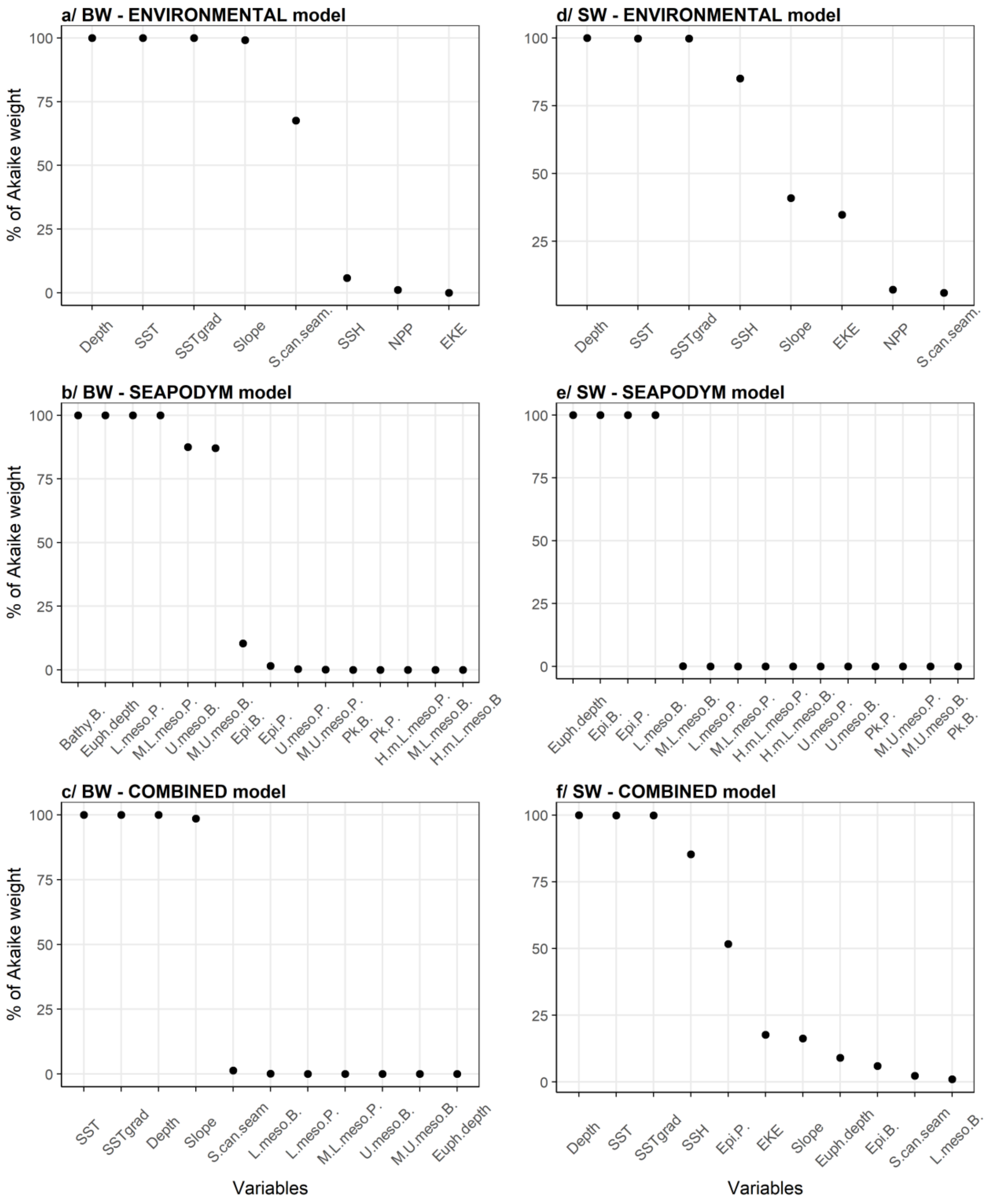

S3.2 Fig. Importance of each variable in the ENVIRONMENTAL, SEAPODYM and COMBINED models for beaked (BW) and sperm (SW) whales. Variables were ranked by summing the Akaike weights of the models in which the variable was selected to obtain a percentage of Akaike weight. We used the 'qpcR' R-package (Spiess, 2014). SST: sea surface height; SSTgrad: gradient of sea surface temperature; NPP: net primary production; SSH: sea surface height; EKE: eddy kinetic energy; S.can.seam: surface of the cell occupied by canyons and seamounts; Euph.depth: Euphotic depth; B.: biomass; P.: production; M.: migrant; H.m.: highly migrant; U: upper; L: lower; Epi.: epipelagic; Meso.: mesopelagic; Pk: zooplankton.

References

Spiess A. 2014. qpcR: Modelling and analysis of real-time PCR data. R package version 1.4-0. Retrieved from https://CRAN.R-project.org/package=qpcR.

Wei, T. & Simko, V. (2017). R package "corrplot": Visualization of a Correlation Matrix (Version 0.84). Available from https://github.com/taiyun/corrplot
