# Supplementary figures and images for: Towards a better characterisation of deep-diving whales’ distributions by using prey distribution model outputs?
Source: PLoS One. 2021 Aug 4;16(8):e0255667. doi: 10.1371/journal.pone.0255667 (PMC8336804; doi:10.1371/journal.pone.0255667)

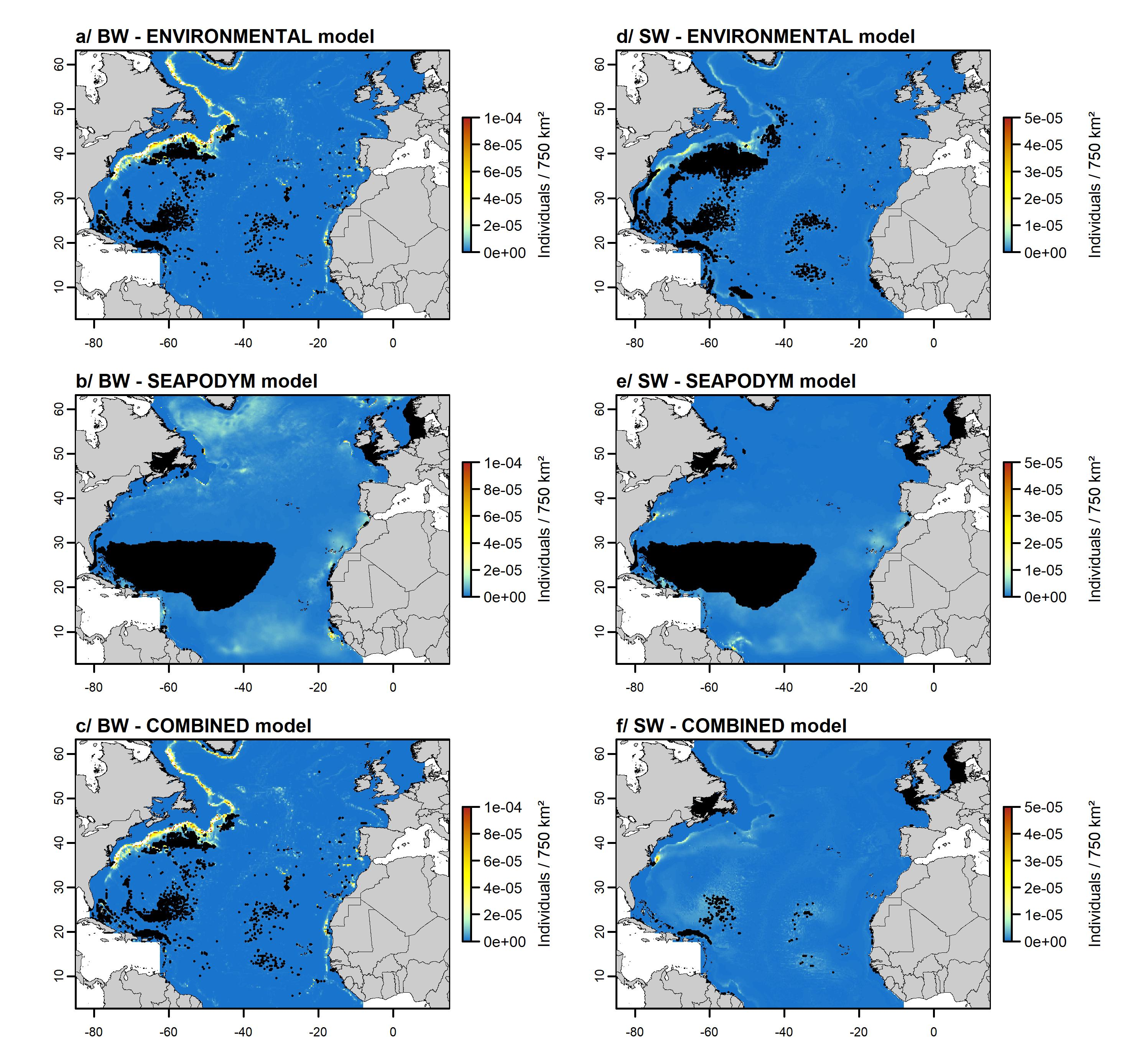

Supplement: S4 Appendix — Black areas represent extrapolation where we did not extrapolate the predictions. Base map from https://www.gebco.net/. (TIF) [file pone.0255667.s004.tif]
