# Supplementary material for: Towards a better characterisation of deep-diving whales’ distributions by using prey distribution model outputs?
Source: PLoS One. 2021 Aug 4;16(8):e0255667. doi: 10.1371/journal.pone.0255667 (PMC8336804; doi:10.1371/journal.pone.0255667)
Supplement: Attachment — Submitted filename: PONE-D-21-03978_reviewer.pdf [file pone.0255667.s005.pdf]

PLOS ONE

Towards a better characterisation of deep-diving whales' distributions by using prey distribution model outputs

--Manuscript Draft--

**Manuscript Number:**	PONE-D-21-03978
**Article Type:**	Research Article
**Full Title:**	Towards a better characterisation of deep-diving whales' distributions by using prey distribution model outputs
**Short Title:**	Habitat modelling with prey distribution model outputs
**Corresponding Author:**	Auriane Virgili<br>La Rochelle University: Universite de La Rochelle<br>La Rochelle, FRANCE
**Keywords:**	Beaked whales; Habitat-based density modelling; SEAPODYM; Sperm whales; prey fields
**Abstract:**	In habitat modelling, environmental variables are assumed to be proxies of lower trophic levels distribution and by extension, of marine top predator distributions. More proximal variables, such as potential prey fields, could refine relationships between top predator distributions and their environment. In situ data on prey distributions are not available over large spatial scales but, a numerical model, the Spatial Ecosystem And POpulation DYnamics Model (SEAPODYM), provides simulations of the biomass and production of zooplankton and six functional groups of micronekton at the global scale. Here, we aimed at exploring if models fitted to simulated prey distribution data better fit deep-diver densities (here beaked and sperm whales) than models fitted to environmental variables and testing whether the combination of environmental and prey distribution data would further improve model fit. We compared the explanatory power of three generalised additive models that used environmental variables ('ENVIRONMENTAL model'), SEAPODYM-derived variables ('SEAPODYM model') and a combination of both variable types. For beaked and sperm whales, the highest relative densities in the ENVIRONMENTAL models where associated with deep depths, strong spatial gradients of sea surface temperature and gentle slopes. In the SEAPODYM models, beaked whales were closely related to low biomasses of bathypelagic, epipelagic and mesopelagic organisms and deep euphotic depth, while sperm whales were closely related to low biomasses of bathypelagic and epipelagic organisms, high production of epipelagic organisms and deep euphotic depth. In models that combined both types of variables, no SEAPODYM variable was selected for beaked whales while for sperm whales, epipelagic biomass and production were selected. SEAPODYM gives new insights about potential prey fields targeted by deep-divers at large spatial scales. Considering current SEAPODYM parameterisation, if predators feed on pelagic prey assemblages, the combined use of prey and environmental variables would represent an improvement through more accurate modelling of their densities.
**Order of Authors:**	Auriane Virgili
	Laura Hedon
	Matthieu Authier
	Beatriz Calmettes
	Diane Claridge
	Tim Cole
	Peter Corkeron
	Ghislain Dorémus
	Charlotte Dunn
	Tim E Dunn

Powered by Editorial Manager® and ProduXion Manager® from Aries Systems Corporation

	Sophie Laran
	Patrick Lehodey
	Mark Lewis
	Maite Louzao
	Laura Mannocci
	José Martinez-Cedeira
	Pascal Monestiez
	Debra Palka
	Emeline Pettex
	Jason J Roberts
	Leire Ruiz
	Camilo Saavedra
	M. Begoña Santos
	Olivier Van Canneyt
	José Antonio Vázquez Bonales
	Vincent Ridoux
**Additional Information:**	
**Question**	**Response**
**Financial Disclosure**<br><br>Enter a financial disclosure statement that describes the sources of funding for the work included in this submission. Review the submission guidelines for detailed requirements. View published research articles from *PLOS ONE* for specific examples.<br><br>This statement is required for submission and **will appear in the published article** if the submission is accepted. Please make sure it is accurate.	AV was funded by the Direction Générale de l'Armement.<br>ML was funded by a Ramón y Cajal (RYC-2012-09897) postdoctoral contract of the Spanish Ministry of Economy, Industry and Competitiveness.<br>The funders had no role in study design, data collection and analysis, decision to publish, or preparation of the manuscript.

Powered by Editorial Manager® and ProduXion Manager® from Aries Systems Corporation

**Unfunded studies**<br>Enter: *The author(s) received no specific funding for this work.*<br><br>**Funded studies**<br>Enter a statement with the following details:<br>• Initials of the authors who received each award<br>• Grant numbers awarded to each author<br>• The full name of each funder<br>• URL of each funder website<br>• Did the sponsors or funders play any role in the study design, data collection and analysis, decision to publish, or preparation of the manuscript?<br>• **NO** - Include this sentence at the end of your statement: *The funders had no role in study design, data collection and analysis, decision to publish, or preparation of the manuscript.*<br>• **YES** - Specify the role(s) played.<br><br>* typeset	
**Competing Interests**<br><br>Use the instructions below to enter a competing interest statement for this submission. On behalf of all authors, disclose any competing interests that could be perceived to bias this work—acknowledging all financial support and any other relevant financial or non-financial competing interests.<br><br>This statement is required for submission and **will appear in the published article** if the submission is accepted. Please make sure it is accurate and that any funding sources listed in your Funding Information later in the submission form are also declared in your Financial Disclosure statement.<br><br>View published research articles from *PLOS ONE* for specific examples.	The authors have declared that no competing interests exist.

**NO authors have competing interests**<br><br>Enter: *The authors have declared that no competing interests exist.*<br><br>**Authors with competing interests**<br><br>Enter competing interest details beginning with this statement:<br><br>*I have read the journal's policy and the authors of this manuscript have the following competing interests: [insert competing interests here]*<br><br>* typeset	
**Ethics Statement**<br><br>Enter an ethics statement for this submission. This statement is required if the study involved:<br><br>• Human participants<br>• Human specimens or tissue<br>• Vertebrate animals or cephalopods<br>• Vertebrate embryos or tissues<br>• Field research<br><br>Write "N/A" if the submission does not require an ethics statement.<br><br>General guidance is provided below. Consult the submission guidelines for detailed instructions. **Make sure that all information entered here is included in the Methods section of the manuscript.**	N/A

Powered by Editorial Manager® and ProduXion Manager® from Aries Systems Corporation

**Format for specific study types**<br><br>**Human Subject Research (involving human participants and/or tissue)**<br>• Give the name of the institutional review board or ethics committee that approved the study<br>• Include the approval number and/or a statement indicating approval of this research<br>• Indicate the form of consent obtained (written/oral) or the reason that consent was not obtained (e.g. the data were analyzed anonymously)<br><br>**Animal Research (involving vertebrate animals, embryos or tissues)**<br>• Provide the name of the Institutional Animal Care and Use Committee (IACUC) or other relevant ethics board that reviewed the study protocol, and indicate whether they approved this research or granted a formal waiver of ethical approval<br>• Include an approval number if one was obtained<br>• If the study involved *non-human primates*, add *additional details* about animal welfare and steps taken to ameliorate suffering<br>• If anesthesia, euthanasia, or any kind of animal sacrifice is part of the study, include briefly which substances and/or methods were applied<br><br>**Field Research**<br><br>Include the following details if this study involves the collection of plant, animal, or other materials from a natural setting:<br>• Field permit number<br>• Name of the institution or relevant body that granted permission	
**Data Availability**<br><br>Authors are required to make all data underlying the findings described fully available, without restriction, and from the time of publication. PLOS allows rare exceptions to address legal and ethical concerns. See the PLOS Data Policy and FAQ for detailed information.	Yes - all data are fully available without restriction

A Data Availability Statement describing where the data can be found is required at submission. Your answers to this question constitute the Data Availability Statement and **will be published in the article**, if accepted.<br><br>**Important:** Stating ‘data available on request from the author’ is not sufficient. If your data are only available upon request, select ‘No’ for the first question and explain your exceptional situation in the text box.<br><br>Do the authors confirm that all data underlying the findings described in their manuscript are fully available without restriction?	
**Describe where the data may be found in full sentences. If you are copying our sample text, replace any instances of XXX with the appropriate details.**<br><br>• If the data are **held or will be held in a public repository**, include URLs, accession numbers or DOIs. If this information will only be available after acceptance, indicate this by ticking the box below. For example: *All XXX files are available from the XXX database (accession number(s) XXX, XXX.).*<br>• If the data are all contained **within the manuscript and/or Supporting Information files**, enter the following: *All relevant data are within the manuscript and its Supporting Information files.*<br>• If neither of these applies but you are able to provide **details of access elsewhere**, with or without limitations, please do so. For example:<br><br>*Data cannot be shared publicly because of [XXX]. Data are available from the XXX Institutional Data Access / Ethics Committee (contact via XXX) for researchers who meet the criteria for access to confidential data.*<br><br>*The data underlying the results presented in the study are available from (include the name of the third party*	All sighting and effort data used in this study are available in the OBIS SEAMAP database: http://seamap.env.duke.edu/. All data providers can be contacted via the OBIS SEAMAP website.

and contact information or URL).<br>• This text is appropriate if the data are owned by a third party and authors do not have permission to share the data.<br><br>* typeset	
Additional data availability information:	

Powered by Editorial Manager® and ProduXion Manager® from Aries Systems Corporation

Manuscript

Click here to access/download;Manuscript;Virgili_et_al_PlosOne.docx

Towards a better characterisation of deep-diving whales' distributions by using prey distribution model outputs

Auriane Virgili [1*], Laura Hedon [1], Matthieu Authier [1,2], Beatriz Calmettes [3], Diane Claridge [4], Tim Cole [5], Peter Corkeron [5], Ghislain Dorémus [1], Charlotte Dunn [4], Tim E. Dunn [6], Sophie Laran [1], Patrick Lehodey [3], Mark Lewis [5], Maite Louzao [7], Laura Mannocci [8], José Martínez-Cedeira [9], Pascal Monestiez [10,11], Debra Palka [5], Emeline Pettex [12,2], Jason J. Roberts [13], Leire Ruiz [14], Camilo Saavedra [15], M. Begoña Santos [15], Olivier Van Canneyt [1], José Antonio Vázquez Bonales [16], Vincent Ridoux [1,11]

[1] Observatoire PELAGIS, UMS 3462 CNRS - Université de La Rochelle, Systèmes d'Observation pour la Conservation des Mammifères et des Oiseaux Marins, 17000 La Rochelle, France; [2] ADERA, 162 avenue Albert Schweitzer, CS 60040, 33608 Pessac Cedex, 33000 Pessac, France; [3] Space Oceanography Division, CLS, 8-10 rue Hermes, Ramonville 31520, France; [4] Bahamas Marine Mammal Research Organisation, P.O. Box AB-20714, Marsh Harbour, Abaco, Bahamas; [5] Protected Species Branch, NOAA Fisheries Northeast Fisheries Science, 166 Water St Woods Hole Massachusetts, 02543 USA; [6] Joint Nature Conservation Committee, Inverdee House, Baxter Street, Aberdeen, AB11 9QA, UK; [7] AZTI, Marine Research, Basque Research and Technology Alliance (BRTA), Herrera Kaia, Portualdea z/g, 20110 Pasaia, Spain; [8] MARBEC, Univ Montpellier, CNRS, Ifremer, IRD, Sète, France; [9] CEMMA, Camiño do Ceán, n° 2, 36350, Nigrán, Pontevedra, Spain; [10] BioSP, INRA, 84914 Avignon, France; [11] Centre d'Etudes Biologiques de Chizé - La Rochelle, UMR 7372 CNRS - Université de La Rochelle, Institut du Littoral et de l'Environnement, 17000 La Rochelle, France; [12] Cohabys - ADERA, Université de La Rochelle 2, rue Olympe de Gouges, Bâtiment ILE, 17000 La Rochelle; [13] Marine Geospatial Ecology Laboratory, Duke University, Durham, North Carolina, USA; [14] AMBAR Elkartea organisation, Ondarreta Ibilbidea z/g, 48620 Plentzia, Bizkaia, Spain; [15] Instituto Español de Oceanografía, Centro Oceanográfico de Vigo, 36390 Vigo, Spain; [16] Alnilam Research and Conservation, Pradillos 29, 28491-Navacerrada, Madrid, Spain.

Corresponding author: Auriane Virgili, 5 Allées de l'Océan 17000 La Rochelle, France.

auriane.virgili@univ-lr.fr

Abstract

In habitat modelling, environmental variables are assumed to be proxies of lower trophic levels distribution and by extension, of marine top predator distributions. More proximal variables, such as potential prey fields, could refine relationships between top predator distributions and their environment. *In situ* data on prey distributions are not available over large spatial scales but, a numerical model, the Spatial Ecosystem And POpulation DYnamics Model (SEAPODYM), provides simulations of the biomass and production of zooplankton and six functional groups of micronekton at the global scale. Here, we aimed at exploring if models fitted to simulated prey distribution data better fit deep-diver densities (here beaked and sperm whales) than models fitted to environmental variables and testing whether the combination of environmental and prey distribution data would further improve model fit. We compared the explanatory power of three generalised additive models that used environmental variables ('ENVIRONMENTAL model'), SEAPODYM-derived variables ('SEAPODYM model') and a combination of both variable types. For beaked and sperm whales, the highest relative densities in the ENVIRONMENTAL models where associated with deep depths, strong spatial gradients of sea surface temperature and gentle slopes. In the SEAPODYM models, beaked whales were closely related to low biomasses of bathypelagic, epipelagic and mesopelagic organisms and deep euphotic depth, while sperm whales were closely related to low biomasses of bathypelagic and epipelagic organisms, high production of epipelagic organisms and deep euphotic depth. In models that combined both types of variables, no SEAPODYM variable was selected for beaked whales while for sperm whales, epipelagic biomass and production were selected. SEAPODYM gives new insights about potential prey fields targeted by deep-divers at large spatial scales. Considering current SEAPODYM parameterisation, if predators feed on pelagic prey assemblages, the combined use of prey and environmental variables would represent an improvement through more accurate modelling of their densities.

Keywords: Beaked whales, Habitat-based density modelling, SEAPODYM, Sperm whales, prey fields

1. Introduction

Cetaceans are subject to many anthropogenic threats such as vessel collisions, pollutants, bycatch and underwater noise, leading to the decline of many populations [1-3]. To develop effective conservation strategies, describing and predicting their distribution is essential because cetacean distributions are barely known due to missing data which is an important pitfall for conservation. Habitat modelling is a useful tool to predict species distributions [4]. In these models, species observations are related to proximal or distal variables that are assumed to influence their distributions. Proximal variables are biological variables to which the species is assumed to react more directly (*e.g.* prey distribution) than distal variables, which describe the physical environment [5]. Distal variables encompass two types of variables; physiographic variables which are static descriptors that relate to the bathymetry (*e.g.* depth, slope) and oceanographic variables which are dynamic ~~descriptors~~ that describe the water masses (*e.g.* sea surface temperature - SST, eddy kinetic energy - EKE, surface chlorophyll *a* concentration). These variables, obtained from satellite imagery or numerical models, are more widely available than proximal data and are often used to describe and predict marine top predator distributions [6-8].

In the marine environment, the surface chlorophyll *a* concentration is commonly used as a proxy for the biomass of phytoplankton in cetacean habitat models [8-10], but the distribution and biomass of low- (phytoplankton and zooplankton) and mid- (micronekton) trophic levels are more proximal predictors of top predator distribution [7,11]. Surface chlorophyll *a* concentration data is widely used because it is derived from satellites and available at a global scale, but there is a time-lag between a change in phytoplankton biomass and its effects on upper trophic levels [7,9,11,12,13]. The use of more proximal variables, such as prey biomass, could reduce this lag because marine top predators are expected to be mostly sensitive to prey abundance [11,14, 15,16,17]. However, the limited spatio-temporal extent of prey data available from *in situ* sampling is a major bottleneck for modelling predator distributions from the distribution of their prey over large oceanic regions. Ecosystem models simulating the biomass and production of low- and mid-trophic levels provide a new numerical way to

fill the prey data gap globally [18-21]. The Spatial Ecosystem And POpulation DYnamics Model (SEAPODYM) provides simulations of the global 3-dimensional distributions of zooplankton and six functional groups of the micronekton defined by their daily vertical migration patterns in three biological layers from 0 to *ca.* 1,000 m. SEAPDOYM was initially developed to model tuna populations for fishery applications [22,23], but its usage was extended to predict turtles, cetaceans and elephant seals habitat use [19,20,21,24].

In a previous study [25], we developed habitat models for deep-diving cetaceans (beaked whales *Ziphiidae*, sperm whales *Physeteriidae* and kogia whales *Kogiidae*) using physiographic variables together with surface oceanographic variables. As deep-diving cetaceans spend most of their time at depth and generally feed on mesopelagic to bathypelagic preys (*e.g.* [26,27]), the use of surface variables may limit the ability to correctly infer their habitats. Consequently, using the same sighting survey data as in Virgili et al. [25], here we aimed to explore (1) if models fitted to simulated prey data explained the beaked and sperm whale distributions better than models fitted to environmental variables only and (2) if the combination of environmental and simulated prey data would further improve model fit. We compared the fit and explanatory power of three Generalised Additive Models (GAMs), fitted to beaked and sperm whales sighting data, that used (1) static variables ~~such as depth and slope and oceanographic variables such as SST and EKE~~ (hereafter 'ENVIRONMENTAL model'), (2) simulated prey distribution variables from SEAPODYM (hereafter 'SEAPODYM model', differing from 'SEAPODYM' that refers to the model that simulated prey distribution data) and (3) a combination of both variable types (hereafter 'COMBINED model'). We then predicted beaked and sperm whale densities in the North Atlantic Ocean and compared the three models. We expected a better explanatory power of the model that combined the two types of variables because it considered static variables known to influence the beaked and sperm whale distribution (*e.g.* [28,29]), together with prey distribution data, presumably better suited to describe the species distributions.

2. Material and methods

2.1. Study area

The study area encompassed the North Atlantic Ocean from the Guiana Plateau to Iceland, *i.e.* approximately from 5-65°N, excluding semi-enclosed seas like the Gulf of Mexico, the Hudson Bay, the Baltic sea and the Mediterranean Sea (Fig. 1a).

In the North Atlantic Ocean, the general thermohaline circulation is characterised by the formation of deep saline and cold-water masses flowing from the Labrador basin and Greenland Sea southward and warmer surface waters generally flowing northward but affected by a large gyre [30,31]. This subtropical gyre is delimited by the North Atlantic, Azores and Canary Currents in the east and the North Equatorial Current and Gulf Stream in the west. The latter is narrower and swifter than its eastern counterparts and follows the continental slope from Florida to the Grand Banks off Newfoundland. Large seasonal variations of the wind field (except in the subtropical area), high salinity and a general decreasing gradient of temperature from west to east (about 8°C difference) are characteristics of the North Atlantic Ocean central gyre [31]. Within this ocean basin, primary production is quite low in the tropical zone, and varies seasonally elsewhere. Maximum productivity is reported in winter in the subtropical zone; a spring bloom and oligotrophic summer conditions are found at mid-latitude; and productivity is maximum in summer in the subpolar zone [32].

Fig. 1. Study area divided into sub-regions showing assembled survey effort (a), along with beaked whale (b) and sperm whale (c) sightings recorded during all surveys. The blue polygon delineates overall study area and other polygons delineate sub-regions. Surveys were carried out along transects following a line-transect methodology (survey details in Appendix S1 in Supporting Information). Sightings were classified by group sizes with each point representing one group of individuals and point size representing the number of animals in a group.

2.2. Data collection and collation

In this study, we used some of the effort and sighting data assembled in Virgili et al. ([25]; Fig.1). We only considered beaked and sperm whale sightings and effort data recorded in the North Atlantic Ocean. We assembled visual shipboard and aerial surveys performed by nine independent organisations in the North Atlantic Ocean between 1998 and 2015 (details of the surveys in Appendix

S1). A single common dataset was created, aggregating all survey datasets standardised for units and formats. Effort data were linearized and divided into 5 km segments using ArcGIS 10.3 [33] and the Marine Geospatial Ecology Tools software [34].

Except for the JNCC-ESAS surveys that used a 300-m strip-transect methodology, cetacean sightings were recorded following line-transect methodologies that allow Effective Strip Width (ESW) to be estimated from the measurement of the perpendicular distances to the sightings [35]. For each taxon, we built a hierarchical model to estimate the ESW depending on observation conditions and survey types (for more details, see [25]).

To account for the difficulty to identify them at species level, beaked whale species were pooled into one group including Cuvier's beaked whales (*Ziphius cavirostris),* mesoplodonts (*Mesoplodon* spp.) and northern bottlenose whales *(Hyperoodon ampullatus)*.

A total of 481 sightings of beaked whales and 542 sightings of sperm whales, mainly distributed in the northeast and the northwest Atlantic Ocean (north of the 35°N latitude) were assembled for the present study (Fig.1b and c). Aggregated data represented about 1,101,200 km of on-effort transects (*i.e.* following a transect at a specified speed and altitude with a specified level of visual effort) of which 58% was carried out by plane and the rest by boat (Fig.1a, Table 1).

Table 1. Effort performed by platform type and Beaufort sea-state per sector in the North Atlantic Ocean. This table presents the total effort conducted in each sector broken down by platform type and Beaufort sea-state. Beaufort sea-state values reported with decimals in the surveys were rounded up. For the analyses, all segments with Beaufort sea-state > 4 were excluded. 'NE-ATL' means northeast Atlantic Ocean and 'NW-ATL' means northwest Atlantic Ocean.

Sectors	Total survey effort (km and %)	Total aerial effort (km)	Total shipboard effort (km)	Total effort by Beaufort sea-state class (km)				
				0-1	1-2	2-3	3-4	4-7
**NE-ATL**	521,000<br>47 %	70,400	450,600	76,700	118,500	135,700	190,000	200
**NW-ATL**	561,100<br>51 %	549,800	11,300	43,000	122,400	200,900	132,700	62,100
**TROPICS**	19,100<br>2 %	15,400	3,700	10,600	2,500	3,700	2,300	0
**STUDY AREA**	1,101,200	635,600<br>58 %	465,600<br>42 %	130 ,300<br>11 %	243,400<br>22 %	340,300<br>31 %	324,900<br>30 %	62,300<br>6 %

2.3. Data processing

2.3.1. Static and oceanographic variables

We considered static and dynamic variables that can affect the distribution of beaked and sperm whales (Table 2). All variables were resampled at a 0.25° resolution to match the spatial resolution of the SEAPODYM variables (Table 2; Appendix S2). Depth and slope were derived from GEBCO-08 30 arc-second database (https://www.gebco.net/; 30 arc-second is approximately equal to 0.008°). The surface of canyons and seamounts per 0.25° cell was calculated in ArcGIS 10.3 from the shapefile of canyons and seamounts provided by Harris et al. [36]. Dynamic variables, ~~which relate~~ to the movements of water masses, were upscaled at a monthly resolution, *i.e.* averaged over the 29 days prior to each sampled day to avoid gaps in remote sensing oceanographic variables and to consider a potential time-lag between an environmental condition and its effect on higher trophic levels. The mean, standard error and gradient of SST were calculated from the GHRSST Level 4 CMC SST v.2.0 ([37], https://podaac.jpl.nasa.gov/dataset/CMC0.2deg-CMC-L4-GLOB-v2.0). Spatial gradients of SST were calculated as the difference between the minimum and maximum SST values found in the eight pixels surrounding any given pixel of the grid. The Aviso ¼° DT-MADT geostrophic currents dataset was used to compute mean and standard deviation of Sea Surface Height (SSH) and EKE (https://www.aviso.altimetry.fr/en/data/products/sea-surface-height-products/global/madt-h-uv.html). Net primary production (NPP) was derived from SeaWIFS and Aqua using the Vertically Generalised Production Model (http://orca.science.oregonstate.edu/1080.by.21608day.hdf.vgpm.s.chl.a.sst.php) and was used as a proxy of prey availability.

2.3.2. SEAPODYM variables

SEAPODYM is a numerical model driven by sea temperature, currents, net primary production and chlorophyll *a* concentration [18,22,38]. It simulates the development in time and space of micronekton and, more recently, zooplankton [18,22,38,39]. In SEAPODYM, the zooplankton is defined as all non-

migratory phytoplanktivorous organisms with a size between 200 µm and 2 cm that live in the epipelagic layer [39].

Micronekton encompasses active swimming organisms in the range of 1-20 g and 2-20 cm and includes fishes, crustaceans and cephalopods [39,40]. Depending on the vertical distribution of the organism biomass; three layers are defined according to the euphotic depth (*i.e.* the layer of sea water that receives enough sunlight for photosynthesis to occur; Fig. 2; [40]), computed according to the VGPM model of Behrenfeld &Falkowski [41]. The epipelagic layer extends from the surface to 1.5*euphotic depth; the mesopelagic layer, between 1.5 and 4.5*euphotic depth and the bathypelagic layer which extends from 4.5 to 10.5*euphotic depth with a maximum set at 1,000 m [40]. Micronekton can undertake nychthemeral vertical migrations between these three layers. According to migration patterns, micronekton includes six functional groups: epipelagic, non-migrant mesopelagic, migrant mesopelagic, non-migrant bathypelagic, migrant bathypelagic and highly migrant bathypelagic organisms (Fig.2; [18,22,40]). These migrations are induced by daylight variations and may be due to a strategy of predator avoidance: during daytime, they dive in deeper waters to avoid predation [42]. Migrant mesopelagic and highly migrant bathypelagic organisms migrate between the epipelagic layer at night and respectively the mesopelagic and bathypelagic layers during daytime while the migrant bathypelagic organisms migrate between the mesopelagic and the bathypelagic layers. For each functional group (zooplankton and micronekton) biomass (in $g.m^{-2}$) and production (in $g.m^{-2}.day^{-1}$) are simulated at a 0.25° resolution. Production is defined as the recruitment of a new cohort of organisms into a micronekton functional group when they reach 1 g body mass (fixed value). A positive relationship of predators with production variables would indicate a relationship with the distribution of smaller and more abundant preys of the functional group while a positive relationship with biomass variables would indicate a relationship with the distribution of larger preys.

Table 2. Candidate environmental and SEAPODYM predictors used for the habitat-based density modelling. All variables were resampled at a 0.25° resolution. A: https://www.gebco.net/; 30 arc-second is approximately equal to 0.0083°. B: Harris et al. (2014). C: Canada Meteorological Centre (2012), https://podaac.jpl.nasa.gov/dataset/CMC0.2deg-CMC-L4-GLOB-v2.0. D: https://www.aviso.altimetry.fr/en/data/products/sea-surface-height-products/global/madt-h-uv.html. E: http://orca.science.oregonstate.edu/1080.by.2160.8day.hdf.vgpm.s.chl.a.sst.php. F: Lehodey et al. (2010). Although it is an oceanographic environmental variable, the euphotic depth was included in the SEAPODYM models because it is this variable that defines the three layers of SEAPODYM.1d: daily; 8d: eight days; 1w: weekly.

Variables used in the study with abbreviations and units	Original Resolution	Sources	Effects on pelagic ecosystems of potential interest to deep-divers
**Physiographic**			
Depth (m)	30 arc sec	A	Deep-divers feed on squids and fish in the deep-water column
Slope (°)	30 arc sec	A	Associated with currents, high slopes induce prey aggregation or enhanced primary production
Surface of canyons and seamounts in a 0.25° cell – S.can.seam (km²)	30 arc sec	B	Deep-divers are often associated with canyons and seamounts structures; the variable indicates the proportion of this habitat in each cell
**Oceanographic**			
Mean sea surface temperature – SST (°C)	0.2°, 1d	C	Variability over time and horizontal gradients of SST reveal front locations, potentially associated with prey aggregation or enhanced primary production
Mean gradient of SST – SSTgrad (°C)	0.2°, 1d	C	
Mean of sea surface height – SSH (m)	0.25°, 1d	D	High SSH is associated with high mesoscale activity and enhanced prey aggregation or primary production
Mean of eddy kinetic energy – EKE ($m^2.s^{-2}$)	0.25°, 1d	D	High EKE relates to the development of eddies and sediment resuspension inducing prey aggregation
Mean of net primary production – NPP ($mgC.m^{-2}.day^{-1}$)	9 km, 8d	E	Net primary production as a proxy of prey availability
**SEAPODYM variables**			
Euphotic depth – Euph. depth (m)	0.25°, 1w	F	Depth of the euphotic zone as proxy of prey availability
Epipelagic biomass and production – Epi. B. ($g.m^{-2}$) and Epi. P. ($g.m^{-2}.day^{-1}$)	0.25°, 1w	F	All these variables relate to the distribution of potential direct or indirect preys of deep-divers.
Mesopelagic biomass and production – Meso. B. ($g.m^{-2}$) and Meso. P. ($g.m^{-2}.day^{-1}$)	0.25°, 1w	F	
Migrant mesopelagic biomass and production – M.meso. B. ($g.m^{-2}$) and M.meso. P. ($g.m^{-2}.day^{-1}$)	0.25°, 1w	F	
Bathypelagic biomass and production – Bathy. B. ($g.m^{-2}$) and Bathy. P. ($g.m^{-2}.day^{-1}$)	0.25°, 1w	F	
Migrant Bathypelagic biomass and production – M.bathy. B. ($g.m^{-2}$) and M.bathy. P. ($g.m^{-2}.day^{-1}$)	0.25°, 1w	F	
Highly migrant bathypelagic biomass and production – H.m.bathy. B. ($g.m^{-2}$) and H.m.bathy. P. ($g.m^{-2}.day^{-1}$)	0.25°, 1w	F	
Zooplankton biomass and production – Pk B. ($g.m^{-2}$) and Pk P. ($g.m^{-2}.day^{-1}$)	0.25°, 1w	F	

As for static and oceanographic environmental variables, SEAPODYM variables were upscaled at a monthly resolution, *i.e.* averaged over the 29 days prior to each sampled day. Due to the absence of the bathypelagic layer on the continental shelf, a zero, and not an NA (for not available), was assigned to the effort segments on the continental shelf to avoid deleting the segments during model fitting.

Fig.2. Vertical repartition of the functional groups of micronekton in SEAPODYM in the water column. The three layers, epi, meso and bathypelagic layers, are the layers defined in SEAPODYM; fishes represent the micronekton functional groups; the red dots represent the zooplankton and the yellow arrows represent the daily vertical migrations of the functional groups between layers.

2.4. Habitat-based density modelling

First, we modelled the distribution of beaked whales and sperm whales based on two habitat models: one with physiographic and oceanographic variables (‘ENVIRONMENTAL model’) and one with SEAPODYM variables (‘SEAPODYM model’). We fitted GAMs [43,44] with a Tweedie distribution to account for over-dispersion in the cetacean count data [45] with the ‘mgcv’ R-package (R-3.3.1. version; [46]). GAMs extend Generalised Linear Models to allow for smooth, nonlinear functions of predictor variables to be determined by observed data rather than by strict parametric relationships [43,44]. The mean number of individuals per segment was linked to the additive predictors with a log-function with a maximum of four number of knots to attenuate the scope for over-fitting [44]. An offset equal to segment length multiplied by twice the ESW or twice the 300 m-strip for JNCC-ESAS surveys was included ([47]; refer to Virgili et al. [25] for the ESW estimation; ESWs were calculated for each combination of platform - plane or boat, class of Beaufort sea-state and class of observation height). We removed combinations of variables with Pearson correlation coefficients higher than |0.7|and tested all models with combinations of one to four variables to avoid excessive complexity [48]. Environmental variables were poorly correlated with each other and fairly poorly correlated with the SEAPODYM variables (Figure S3.1 in Appendix S3). However, some SEAPODYM variables were correlated with each other, notably the biomass and production variables in the same layer. Model selection was done with the Akaike information criterion (AIC, the lower the better; [49] and Akaike weight (‘qpcR’ package; [50]).

Then we built a model, for beaked whales and sperm whales, which combined environmental and SEAPODYM variables (‘COMBINED model’). To limit the computational burden, we reduced the number of variables implemented in the variable selection procedure of the COMBINED model. Instead of choosing among the 23 available variables (8 environmental variables and 15 SEAPODYM variables),

we selected only the variables that were selected in the five best ENVIRONMENTAL and SEAPODYM models (ordered by AIC) *i.e.* 7 environmental variables and 7 SEAPODYM variables. As for the ENVIRONMENTAL and SEAPODYM models, all models with combinations of one to four variables (among the 14 available variables) were tested and the best COMBINED model with the lowest AIC was selected. By design, we expected a better fit of the COMBINED model because we only used the best variables selected in the first procedure.

Predictions of relative densities were then provided at 0.25° resolution over the entire study region. There were not enough data to fit a model by month or by season (the number of sightings in winter was too low) so we fitted ENVIRONMENTAL, SEAPODYM and COMBINED models to all data of beaked whales and sperm whales but we predicted relative densities for each month of the period and averaged values to obtain climatological prediction maps for the 1998-2015 period. These predictive maps provided the expected distribution of beaked and sperm whales according to static and monthly oceanographic conditions, or prey distributions or a combination of both. To provide uncertainty maps over the 1998-2015 period, averaged variances around the overall predictions were computed as the mean of the standard errors of the monthly predictions; high variances indicate high errors associated with density estimates. Here we only considered uncertainty associated with the densities predicted with the generalised additive model but we ignored other sources of uncertainty such as those associated with the estimation of the SEAPODYM variables.

To model distributions of beaked whales and sperm whales in the North Atlantic Ocean, we gathered survey data from a large region collected over a long period. The cumulative effort was not homogeneously distributed and showed extensive geographical gaps. Consequently, predicting across the entire Atlantic basin would require extensive geographical extrapolation. Therefore, we conducted a gap analysis on environmental space coverage to identify areas where habitat models could produce reliable predictions outside survey blocks, *i.e.* geographical extrapolation, whilst remaining within the ranges of surveyed conditions for the combinations of covariates selected by the models, *i.e*. areas of environmental interpolation ([51-53], as in Virgili et al. [25]. We estimated the convex hull defined by

the environmental data used to fit habitat models *i.e.* the smallest convex envelop that contains all the points. We assessed whether a prediction from a set of environmental covariates fell inside or outside this convex hull with the "WhatIf" R package [54-56]. A combination of climatological predictor values that fall inside the convex hull corresponds to an interpolation. We produced maps delineating the extent of interpolation areas and overlaid them on the density prediction maps to highlight areas with greater reliability and showing that no prediction was made outside interpolation areas.

Finally, the model fit of each model was assessed thanks to the explained deviance [44] and the AIC. This allowed a comparison of the three models in order to assess which variables better explained the distribution of beaked and sperm whales.

3. Results

Beaked whales

The percentage of null deviance explained varied between the three models (Fig.3; Table S3.1 in Appendix S3). The explained deviances of the ENVIRONMENTAL and COMBINED models were identical (40.7%) and higher than the explained deviance of the SEAPODYM model (29.0%). The AICs were the lowest for the ENVIRONMENTAL and COMBINED models (ΔAIC = 196 with the SEAPOYM model; Fig.3).

In the best ENVIRONMENTAL model (Fig.3; Table S3.1 in Appendix S3), the highest beaked whale relative densities were found at depths *ca.* 2,20[illegible] m, spatial gradients of SST *ca.* 2.5 °C, slope *ca.* 1° and surface of canyons and seamounts *ca.* 200 km² in the 5–95% percentile intervals (Fig.3a). All selected variables were poorly correlated (Fig.S3.1 in Appendix S3). The highest relative densities were predicted on the western side of the North Atlantic Ocean near the Gulf Stream, along continental slopes and along the Mid-Atlantic Ridge (Fig.4a).

In the best SEAPODYM model (Fig.3; Table S3.1 in Appendix S3), all selected variables were poorly correlated [illegible]Fig.S3.1 in Appendix S3), the highest beaked whale densities were found at lo[illegible]iomasses of bathypelagic (*ca.* 3 g.m^{-2}), epipelagic [illegible] 2 g.m^{-2}) and mesopelagic (*ca.* 0.01 g.m^{-2}) organisms per unit surface area and deep euphotic depth (*ca.* 65 m) in the 5–9[illegible]% percentile intervals (Fig.3b). This

resulted in a fairly homogenous prediction of beaked whales in oceanic waters beyond the continental slope (Fig.4b).

The COMBINED model pooled variables of the ENVIRONMENTAL and the SEAPODYM models and was identical to the environmental model as no SEAPODYM variable was selected (Fig.3c; Table S3.1 in Appendix S3). This resulted in the same distribution patterns as for the ENVIRONMENTAL model (Fig.4c).

Interpolation areas varied between the three models with the highest percentage of interpolation observed for the ENVIRONMENTAL and COMBINED models (97%) and the lowest for the SEAPODYM model (84%; Fig.4). In all cases, variances associated with the predictions were small (Appendix S4).

Fig.3. The functional relationships between beaked (BW) and sperm (SW) whales and the selected variables of the ENVIRONMENTAL (a, d), SEAPODYM (b, e) and COMBINED (c, f) models. Solid lines represent the estimated smooth functions and the blue shaded regions show the approximate 95% confidence intervals. The relative density of individuals (individuals per 750 km²) is shown on the y-axis, where a zero indicates no effect of the covariate. Red vertical lines indicate the 2.5th and 97.5th percentile intervals corresponding to the core data. (D*: explained deviance; AIC: Akaike criterion; SSTgrad: gradients of sea surface temperature; S.can.seam: surface of the cell occupied by canyons and seamounts; Euph. depth: euphotic depth; Bathy. B.: bathypelagic biomass; Epi. B.: epipelagic biomass; Meso. P.: mesopelagic production; EKE: eddy kinetic energy; Epi. P.: epipelagic production).

Sperm whale

The percentage of null deviance explained varied between the three models (Fig.3; Table S3.2 in Appendix S3). The explained deviance of the ENVIRONMENTAL model (29.9%) was similar to that of the COMBINED model (30.1%) and higher than that of the SEAPODYM model (25.7%). The AICs were identical in the ENVIRONMENTAL and COMBINED model and lower than the AIC of the SEAPODYM model (ΔAIC = 74 with the SEAPOYM model; Fig.3).

In the best ENVIRONMENTAL model (Fig.3; Table S3.2 in Appendix S3), the highest sperm whale densities were found at depths *ca.* 2,200 m, high EKE (log(EKE) > -5 m².s^{-2}), spatial gradients of SST higher than 2.5°C and slope *ca.* 1° in the 5–95% percentile intervals (Fig.3d). All selected variables were poorly correlated (Fig.S3.1 in Appendix S3). The highest densities were predicted on the western side

of the North Atlantic Ocean near the Gulf Stream, along continental slopes and along the Mid-Atlantic Ridge (Fig.4d).

In the best SEAPODYM model (Fig.3; Table S3.2 in Appendix S3), all selected variables were poorly correlated (Fig.S3.1 in Appendix S3), the highest densities were found at low biomasses of bathypelagic (*ca.* 3 g.m^{-2}) and epipelagic (*ca.* 0.01 g.m^{-2}) organisms, production of epipelagic organisms higher than 0.25 g.m^{-2}.day^{-1} and deep euphotic depth higher than 75 m in the 5–95% percentile intervals (Fig.3e). This resulted in fairly homogenous predicted densities in oceanic waters with the highest densities predicted in the tropical waters and near the north-western African coast (Fig.4e).

In the best COMBINED model (Fig.3; Table S3.2 in Appendix S3), all selected variables were poorly correlated (Fig.S3.1 in Appendix S3), the highest densities were found at depths *ca.* 2,200 m, low biomass (*ca.* 0.01 g.m^{-2}) of epipelagic organisms, production of epipelagic organisms higher than 0.25 g.m^{-2}.day^{-1} and gradients of SST *ca.* 2.5°C in the 5–95% percentile intervals (Fig.3f). This resulted in similar patterns as for the ENVIRONMENTAL model but with more homogenous densities (Fig.4f).

Interpolation areas varied between the three models and were less extended for the SEAPODYM (85%) and ENVIRONMENTAL models (94%) compared to the COMBINED model (97%; Fig.4). In all cases, variances associated with the predictions were small (Appendix S4).

Fig. 4. The predicted relative densities of beaked (BW) and sperm (SW) whales obtained from the ENVIRONMENTAL (a, d), SEAPODYM (b, e) and COMBINED (c, f) models. Black areas on prediction maps represent zones where we did not extrapolate the predictions. Percentages represent the proportion of the study area defined as interpolation (Inter.) with the gap analysis.

4. Discussion

Indirect proxies of prey distribution such as SST or EKE are routinely used in habitat models to describe marine top predator distributions because prey data are generally unavailable. The relationships estimated between these proxies and predator occurrence or abundance are therefore indirect. The integration of higher trophic levels in habitat models could improve the model performances and the ecological explanation of the relationships, as shown in Lambert et al. [20] or

Putra & Mustika [57]. In this study, we incorporated potentially more direct variables into habitat-based density models by using simulated distributions of functional groups of prey. The objective was first to compare models using environmental variables to models using simulated prey distribution data as variables in order to evaluate the explanatory power of both types of models. Second, to combine the two types of variables in a single model to determine whether their combination would improve model fit to the data. The models were fitted to sightings of deep-diving cetaceans (beaked and sperm whales) because their habitat use is still poorly known. For species that spend most of their time at depth, like deep-divers, information on potential prey in deep layers could improve current habitat models because they are mostly based on surface variables and not on variables that characterise deep layers [25,28,29,58].

Here we built basin-wide models and did not investigate region-specific models (*sensu* [59]) in order to leverage large sample sizes for investigating the explanatory power of SEAPODYM variables for habitat use of deep-divers. We acknowledge that pooling regions across an oceanic basin may introduce bias, but this study is a first investigation, and appreciate region-specific models would represent an obvious improvement (once enough data are available at the regional scale).

The model comparison showed that for beaked whales, the explained deviance was higher and the AIC lower in the ENVIRONMENTAL model than in the SEAPODYM model, so environmental variables seemed to better explain their distribution than SEAPODYM variables; this could explain why no SEAPODYM variable was selected in the COMBINED model. As for Torres et al. [60], it is possible that the integration of prey data did not improve the explanatory power of the models because the prey sampling was not appropriate or there was a mismatch between the distribution of prey and predators because they respond to oceanographic processes at different spatial and temporal scales. The link between predators and the environment may therefore be simpler to model than the link between predators and prey [60]. In contrast, for sperm whales, the ENVIRONMENTAL and COMBINED models were comparable but the explained deviance and AIC were slightly better for the COMBINED model so SEAPODYM variables seemed to provide additional information to that provided by the

environmental variables. These results agreed with those of Hazen et al. [15] who showed that the performance of a model which included static (depth), oceanographic (salinity and temperature) and biological (deep scattering layer and number of available prey) variables was higher than that of the model which only included prey data. One hypothesis, suggested by Hazen et al. [15], would be that beaked whales do not necessarily track a particular type of prey but rather large-scale physiographic structures while sperm whales would be more pelagic predators, so the prey simulated by SEAPDOYM would correspond more closely to the prey targeted by sperm whales than by beaked whales

However, the prey functional groups that were simulated from SEAPODYM may not directly reflect the prey targeted by deep-divers. The three biological layers of SEAPODYM are defined between 0 and *ca.* 1,000 m with organisms ranging from 2 to 20 cm [39,40], but deep-divers can feed beyond 1,000 m on prey bigger than 20 cm [27,61,62]. From stomach contents, Spitz et al. [27] showed that the diet of Cuvier's beaked whales consisted largely of cephalopods bigger than 20 cm, *Teuthowenia megalops* (21,2 ± 3,3 cm) and *Galiteuthis armata* (24,2 ± 2,4 cm) and the diet of sperm whales was mostly composed of cephalopods close to 20 cm, *Histiotheuthis bonnellii bonnellii* (17,8 ± 3,7 cm) in the north-east Atlantic Bay of Biscay. Thus, the functional groups of preys simulated in SEAPODYM may not be directly targeted by deep-divers but the probable link could be mediated by deep-diver prey instead. The prey simulated in SEAPODYM could correspond more closely to the prey of the prey targeted by deep-divers. A similar phenomenon was observed in the study of Putra & Mustika [57] which showed that the zooplankton biomass was a strong predictor of the distribution of manta rays (*Mobula birostris*) because they feed directly on zooplankton whereas it was not a strong predictor for dolphins (*Stenella* spp.) because they are second- and third-level consumers. Nevertheless, the study shed light on dolphin foraging habits. It is likely the same situation in our study, the prey targeted by deep-divers may be larger than those simulated in SEAPODYM [27], therefore the simulated trophic levels may be lower than those actually targeted by deep-divers. This may explain why epipelagic organisms were retained in the models, many bathypelagic squids and fishes are vertically migrating predators feeding at night in the epipelagic layer. In addition, the functional groups simulated by

SEAPODYM encompass multiple organisms (fishes, cephalopods, crustaceans; [39,40]), so deep-diver preys, which are mainly cephalopods, might be poorly represented in the functional groups in terms of biomass and production, even in mesopelagic and bathypelagic layers, explaining why they were not selected in the COMBINED models. The combination of sighting data from different ecosystems (*e.g.* North-East and North-West Atlantic) may also mask inter-regional differences in the relationships between cetacean densities and the environmental predictors, as shown by Mannocci et al. [59]. However, the objective here, was to compare models using environmental variables, prey distribution variables, and a combination of the two types of variables to determine the extent to which the models could be improved and not necessarily explain precisely the mechanisms influencing the distribution of beaked and sperm whales, which would be more consistent at the scale of a smaller region.

Although it would be more consistent to fit models at a regional scale, we highlighted interesting relationships with the environment and/or the simulated prey at a large scale. The highest relative densities in the ENVIRONMENTAL models were associated with deep depths, strong spatial gradients of SST and gentle slopes for both beaked whales and sperm whales, and also with the presence of canyons and seamounts for beaked whales and with high EKE for sperm whales. This suggests a preference for habitats associated with topographic features and thermal fronts, as previously shown [25,28,29]. Comparatively, SEAPODYM variables reveal areas of prey concentration whereas environmental variables are only indirect proxies of the prey distribution. Putra & Mustika [57] suggested that chlorophyll *a* concentrations are not spatially correlated to high zooplankton biomasses because of the time lag between phytoplankton development and meso-zooplankton growth, therefore dolphin (*Stenella* spp.) densities would be higher where zooplankton biomasses would be higher but where chlorophyll *a* concentrations would be lower. SEAPODYM variables could thus give a better idea of the foraging areas used by the species [18,22,40]. We found that beaked whales were closely related to low biomasses of bathypelagic, epipelagic and mesopelagic organisms and deep euphotic depth, while sperm whales were closely related to low biomasses of bathypelagic and epipelagic organisms, high production of epipelagic organisms and deep euphotic depth. This suggests

a preference for moderately productive offshore habitats. ~~In the COMBINED models, no SEAPODYM variable was selected for beaked whales, *i.e.* the model was identical to the environmental model, whereas for sperm whales, two SEAPODYM variables (biomass and production of epipelagic organisms) and two environmental variables (depth and gradients of SST) were selected~~, suggesting a preference for habitats associated with topographic features and thermal fronts for beaked whales and sperm whales but the distribution of sperm whales would also be influenced by the biomass and production of pelagic prey. If beaked whale were more associated ~~to~~ physiographic features around 2000 m deep, they might forage on organisms living close to the seabed that may be poorly modelled by SEAPODYM. By contrast, sperm whales would be less constrained by the presence of the slope, and would therefore prey on organisms that are truly pelagic, and therefore possibly better modelled by SEAPODYM.

The euphotic depth, which is the maximum depth of the light zone suitable for phytoplankton photosynthesis, was selected for both beaked whales and sperm whales in the SEAPODYM models with maximum densities predicted around 65 m for beaked whales while for sperm whales we obtained an increasing relationship with the euphotic depth. This suggests that beaked whales would be more likely to be found in continental slope areas with a moderate depth of the euphotic zone (50-70 m; see Appendix S2), whereas sperm whales would be more dispersed offshore.

For both beaked and sperm whales, depth was selected in COMBINED models. There is a great interest in including depth in the models because it is a well-measured variable, *i.e.* with low associated measurement error, unlike remote sensed or modelled variables. Physiographic variables such as depth, because measured with little error compared to other variables in the model, may help in improving the explanatory power of the models. Pendleton et al. [63] observed similar results for bowhead whales (*Balaena mysticetus*). They compared a model using only environmental and biological variables such as sea ice thickness, sea temperature, diatoms, flagellates, copepods and zooplankton from the Biology Ice Ocean Modeling and Assimilation System (BIOMAS) to a model using these variables plus bathymetry and showed that the best model included bathymetry and BIOMAS

variables. The importance of bathymetry suggests there is probably a close relationship between bathymetry and the biophysical processes that determine the distribution of these cetaceans.

Overall, prediction maps of species relative densities were similar between the three models for beaked and sperm whales with highest densities predicted offshore along continental slopes where prey aggregate [64,65], but prediction maps were smoother with SEAPODYM variables than with environmental variables, as previously showed in Lambert et al. [20]. This may be due to the large resolution of the variables. The spatial resolution of the SEAPODYM variables is currently set to 0.25° but is planned to be refined to 0.125° and a finer spatial resolution may reduce this smooth effect. However, by adding environmental variables, particularly depth, in the sperm whale COMBINED model, the prediction was more spatially detailed.

The interpolation areas varied between the different models, from 84/85% for the SEAPODYM models to 97%/94% for the ENVIRONMENTAL models and 97% for the COMBINED models, which means that 15% of the area represented an environmental extrapolation with the SEAPODYM models, compared to only 3% with the combined models. The uncertainties were thus much greater with the SEAPDYM models and it appeared riskier to perform models at a large scale with SEAPODYM variables only, than with environmental variables. However, by combining both types of variables, this risk was greatly reduced compared to the SEAPODYM models. Thus, areas of species concentration seemed more spatially detailed and better characterised with the COMBINED models. To meet species conservation objectives, it seems more valuable to use models combining environmental variables and prey distribution data. Habitat models are regularly used to identify areas where species concentrate in order to set up protection areas [66,67,68]. They are also useful for predicting species distribution in non-sampled or poorly documented areas [69,70], provided that areas of interpolation and extrapolation are considered [25,51,52]. Considering key habitats of species, particularly foraging habitats, in the design of marine protected areas thanks to the combination of environmental and prey variables would thus represent an improvement in the tools available for species conservation, and

consideration could be given to reducing the overlap between impacting human activities and these key habitats [71,72].

5. Conclusion

Prey distribution data are not widely available to model the distribution of marine top predators. However, there are models, still not widely used, such as SEAPODYM, which simulate the distribution of functional groups of prey. Our approach showed the possibility of improving the explanatory power of habitat-based density models by using a combination of physiographic and oceanographic variables and variables that estimate expected prey availability. The areas of sperm whale concentrations seemed better explained by the model that combined both types of variables. This was not the case for beaked whales, possibly because they prey on organisms more associated to seabed features and consequently less well predicted by SEAPODYM that focus on pelagic prey assemblages. We highlighted the importance of the physiographic variables to understand static mechanisms that influence the distribution of deep-diving cetaceans. The use of more direct estimates of prey availability, rather than oceanographic proxies, could be important for predicting species distributions because the relationships between proxies and prey may change in a climate change context.

Although SEAPODYM outputs alone did not seem ideal for modelling prey of deep-diving cetaceans, we were able to better define the areas of concentration of these species. To improve these results, it would be interesting to fit region-specific models or to apply this approach to species whose functional groups of prey would be better represented in SEAPODYM such as baleen whales or small delphinids. The development of SEAPODYM, in particular the increase in spatial resolution of the variables and the inclusion of measurement errors in SEAPODYM variables, could make SEAPODYM an essential data source to help improving knowledge on species and to be included in management procedures that aimed at improving the conservation of emblematic species such as cetaceans.

Acknowledgments

We are grateful to the many observers who participated in the surveys and collected all the data but also the ships' captains, crews and pilots. We thank Phil Hammond and his team for providing SCANS and CODA survey data. THUNNUS survey was carried out thanks to the collaboration of the General Directorate of Fisheries and Maritime Affairs, Government of Galicia. We thank the *Direction Générale de l'Armement* (DGA), including Odile Gérard and Carole Nahum, for funding Auriane Virgili's doctoral research grant. ML was funded by a Ramón y Cajal (RYC-2012-09897) postdoctoral contract of the Spanish Ministry of Economy, Industry and Competitiveness. This study is a contribution to the CHALLENGES (CTM2013-47032-R) project of the Spanish Ministry of Economy, Industry and Competitiveness.

Data accessibility

All sighting and effort data used in this study are available in the OBIS SEAMAP database: http://seamap.env.duke.edu/. All data providers can be contacted via the OBIS SEAMAP website.

References

[1] Schipper, J., Chanson, J.S., Chiozza, F., Cox, N.A., Hoffmann, M., et al. 2008. The Status of the World's Land and Marine Mammals: Diversity, Threat, and Knowledge. Science, 322: 225–230.

[2] Avila, I.C., Kaschner, K., & Dormann, C.F. 2018. Current global risks to marine mammals: Taking stock of the threats. Biological Conservation, 221: 44-58.

[3] Panti, C., Baini, M., Lusher, A., Hernandez-Milan, G., Rebolledo, E.L.B., Unger, B., ... & Fossi, M.C. 2019. Marine litter: one of the major threats for marine mammals. Outcomes from the European Cetacean Society workshop. Environmental pollution, 247: 72-79.

[4] Elith, J., & Leathwick, J.R. 2009. Species distribution models: ecological explanation and prediction across space and time. Annual review of ecology, evolution, and systematics, 40: 677-697.

[5] Guisan, A., & Zimmermann, N.E. 2000. Predictive habitat distribution models in ecology. Ecological modelling, 135(2-3): 147-186.

[6] Yen, P.P., Sydeman, W.J., & Hyrenbach, K.D. 2004. Marine bird and cetacean associations with bathymetric habitats and shallow-water topographies: implications for trophic transfer and conservation. Journal of Marine systems, 50(1-2): 79-99.

[7] Redfern, J.V., Ferguson, M.C., Becker, E.A., Hyrenbach, K.D., Good, C. et al. 2006. Techniques for cetacean – habitat modeling. Marine Ecology Progress Series, 310: 271–295.

[8] Lambert, C., Laran, S., David, L., Dorémus, G., Pettex, E., Van Canneyt, O., & Ridoux, V. 2017. How does ocean seasonality drive habitat preferences of highly mobile top predators? Part I: the north-western Mediterranean Sea. Deep Sea Research Part II: Topical Studies in Oceanography, 141: 115-132.

[9] Jaquet, N., & Whitehead, H. 1996. Scale-dependent correlation of sperm whale distribution with environmental features and productivity in the South Pacific. Marine ecology progress series: 1-9.

[10] Ferguson, M.C., Barlow, J., Reilly, S.B., Gerrodette, T. 2006. Predicting Cuvier's (*Ziphius cavirostris*) and Mesoplodon beaked whale population density from habitat characteristics in the eastern tropical Pacific Ocean. Journal of Cetacean Research and Management, 7: 287–299.

[11] Grémillet, D., Lewis, S., Drapeau, L., van Der Lingen, C. D., Huggett, J. A., Coetzee, J. C., ... & Ryan, P. G. 2008. Spatial match–mismatch in the Benguela upwelling zone: should we expect chlorophyll and sea-surface temperature to predict marine predator distributions? Journal of Applied Ecology, 45(2): 610-621.

[12] Austin, D., Bowen, W.D., McMillan, J.I., Iverson, S.J. 2006. Linking Movement, Diving, and Habitat to Foraging Success in a Large Marine Predator. Ecology, 87: 3095–3108.

[13] Cotté, C., Guinet, C., Taupier-Letage, I., Mate, B., Petiau, E. 2009. Scale-dependent habitat use by a large free-ranging predator, the Mediterranean fin whale. Deep Sea Research Part I: Oceanographic Research Papers, 56: 801–811.

[14] Österblom, H., Olsson, O., Blenckner, T., & Furness, R.W. 2008. Junk-food in marine ecosystems. Oikos, 117: 967–977.

[15] Hazen, E.L., Nowacek, D.P., Laurent, L.S., Halpin, P.N., & Moretti, D.J. 2011. The relationship among oceanography, prey fields, and beaked whale foraging habitat in the Tongue of the Ocean. PloS one, 6(4): e19269.

[16] Louzao, M., Valeiras, J., García-Barcelona, S., González-Quirós, R., Nogueira, E., Iglesias, M., ... & Pierce, G.J. 2019. Marine megafauna niche coexistence and hotspot areas in a temperate ecosystem. Continental Shelf Research, 186: 77-87.

[17] Rockwood, R.C., Elliott, M.L., Saenz, B., Nur, N., & Jahncke, J. 2020. Modeling predator and prey hotspots: Management implications of baleen whale co-occurrence with krill in Central California. PloS one, 15(7) : e0235603.

[18] Lehodey, P., Murtugudde, R., Senina, I. 2010. Bridging the gap from ocean models to population dynamics of large marine predators: a model of mid-trophic functional groups. Progress in Oceanography, 84(1): 69-84.

[19] Abecassis, M., Senina, I., Lehodey, P., Gaspar, P., Parker, D., Balazs, G., Polovina, J. 2013. A model of loggerhead sea turtle (*Caretta caretta*) habitat and movement in the oceanic North Pacific. PLoS One, 8(9): e73274.

[20] Lambert, C., Mannocci, L., Lehodey, P., Ridoux, V. 2014. Predicting cetacean habitats from their energetic needs and the distribution of their prey in two contrasted tropical regions. PloS one, 9(8): e105958.

[21] Green, D.B., Bestley, S., Trebilco, R., Corney, S.P., Lehodey, P., McMahon, C.R., ... & Hindell, M.A. 2020. Modelled mid-trophic pelagic prey fields improve understanding of marine predator foraging behaviour. Ecography, 43:1-13.

[22] Lehodey, P., Senina, I., Murtugudde, R. 2008. A spatial ecosystem and populations dynamics model (SEAPODYM) – Modeling of tuna and tuna-like populations. Progress in Oceanography, 78(4): 304-318.

[23] Briand, K., Molony, B., & Lehodey, P. 2011. A study on the variability of albacore (*Thunnus alalunga*) longline catch rates in the southwest Pacific Ocean. Fisheries Oceanography, 20(6): 517-529.

[24] Pérez-Jorge, S., Tobeña, M., Prieto, R., Vandeperre, F., Calmettes, B., Lehodey, P., & Silva, M.A. 2020. Environmental drivers of large-scale movements of baleen whales in the mid-North Atlantic Ocean. Diversity and Distributions, 26: 683–698.

[25] Virgili, A., Authier, M., Boisseau, O., Cañadas, A., Claridge, D., Cole, T., ... & Dunn, C. 2019. Combining multiple visual surveys to model the habitat of deep-diving cetaceans at the basin scale. Global Ecology and Biogeography, 28(3): 300-314.

[26] Perrin, W.F., Würsig, B., & Thewissen, J.G.M. (Eds.). 2009. Encyclopedia of marine mammals. Academic Press.

[27] Spitz, J., Cherel, Y., Bertin, S., Kiszka, J., Dewez, A., & Ridoux, V. 2011. Prey preferences among the community of deep-diving odontocetes from the Bay of Biscay, Northeast Atlantic. Deep Sea Research I, 58: 273–282.

[28] Roberts, J.J., Best, B.D., Mannocci, L., Fujioka, E., Halpin, P.N., Palka, D.L., … Lockhart, G.G. 2016. Habitat-based cetacean density models for the U.S. Atlantic and Gulf of Mexico. Scientific Report, 6.

[29] Rogan, E., Cañadas, A., Macleod, K., Santos, M.B., Mikkelsen, B., Uriarte, A., … Hammond, P.S. 2017. Distribution abundance and habitat use of deep diving cetaceans in the North-East Atlantic. Deep Sea Research Part II: Topical Studies in Oceanography, 141: 8–19.

[30] Levin, L.A., & Gooday A. 2003. The Deep Atlantic Ocean. Ecosystems of the deep oceans. (Tyler PA, Ed.). pp. 111-178. Amsterdam; New York: Elsevier.

[31] Tomczak, M., & Godfrey, J.S. 2003. Regional oceanography: an introduction. Elsevier.

[32] Campbell, J.W., & Aarup, T. 1992. New production in the North Atlantic derived from seasonal patterns of surface chlorophyll. Deep Sea Research Part A. Oceanographic Research Papers, 39: 1669–1694.

[33] ESRI. 2016. ArcGIS – A complete integrated system. Redlands, CA: Environmental Systems Research Institute Inc. Retrieved from http://esri.com/arcgis.

[34] Roberts, J.J., Best, B.D., Dunn, D.C., Treml, E.A., & Halpin, P.N. 2010. Marine geospatial ecology tools: An integrated framework for ecological geoprocessing with ArcGIS, Python, R, MATLAB, and C++. Environmental Modelling and Software, 25: 1197–1207.

[35] Buckland, S.T., Rexstad, E.A., Marques, T. A., & Oedekoven, C.S. 2015. Distance sampling: methods and applications. Springer.

[36] Harris, P.T., Macmillan-Lawler, M., Rupp, J., & Baker, E.K. 2014. Geomorphology of the oceans. Marine Geology, 352: 4–24.

[37] Canada Meteorological Center. 2012. GHRSST level 4 CMC0.2deg global foundation sea surface temperature analysis (GDS version 2). Ver. 2.0. PO.DAAC. Pasadena, CA.

[38] Lehodey, P., Andre, J.M., Bertignac, M., Hampton, J., Stoens, A., Menkès, C., Memeryn L., Grima, N. 1998. Predicting skipjack tuna forage distributions in the equatorial Pacific using a coupled dynamical bio-geochemical model. Fisheries Oceanography, 7(3-4): 317-325.

[39] Conchon, A. 2016. Modeling marine zooplankton and micronekcton (Doctoral dissertation, La Rochelle University).

[40] Lehodey, P., Conchon, A., Senina, I., Domokos, R., Calmettes, B., Jouanno, J., Hernandez, O., Kloser, R. 2014. Optimization of a micronekton model with acoustic data. ICES Journal of Marine Science, 72(5): 1399-1412.

[41] Behrenfeld, M.J., & Falkowski, P.G. 1997. A consumer's guide to phytoplankton primary productivity models. Limnology and Oceanography, 42: 1479–1491.

[42] Hays, G.C. 2003. A review of the adaptive significance and ecosystem consequences of zooplankton diel vertical migrations. In Migrations and Dispersal of Marine Organisms (pp. 163-170). Springer, Dordrecht.

[43] Hastie, T. & Tibshirani, R. 1986. Generalized Additive Models. Statistical Science, 3: 297-313.

[44] Wood, S.N. 2006. On confidence intervals for generalized additive models based on penalized regression splines. Australian & New Zealand Journal of Statistics, 48: 445–464.

[45] Foster, S.D., & Bravington, M.V. 2013. A Poisson-Gamma model for analysis of ecological non-negative continuous data. Environmental and ecological statistics, 20: 533–552.

[46] Wood, S. 2013. mgcv: Mixed GAM Computation Vehicle with GCV/AIC/REML smoothness estimation. Retrieved 7 July 2014, from http://cran.r-project.org/web/packages/mgcv/index.html.

[47] Hedley, S.L., & Buckland, S.T. 2004. Spatial models for line transect sampling. Journal of Agricultural, Biological, and Environmental Statistics, 9(2): 181–199.

[48] Mannocci, L., Catalogna, M., Dorémus, G., Laran, S., Lehodey, P., Massart, W., Monestiez, P., Van Canneyt, O., Watremez, P., Ridoux, V. 2014. Predicting cetacean and seabird habitats across a productivity gradient in the South Pacific gyre. Progress in Oceanography, 120: 383–398.

[49] Burnham, K.P., Anderson, D.R. 2002. *Model selection and multimodel inference*, 2nd edn. Springer, New York.

[50] Spiess, A. 2014. qpcR: Modelling and analysis of real-time PCR data. R package version 1.4-0. Retrieved from https://CRAN.R-project.org/package=qpcR.

[51] Jennings, M.D. 2000. Gap analysis: Concepts, methods, and recent results. Landscape Ecology, 15: 5–20.

[52] Mannocci, L., Roberts, J.J., Halpin, P.N., Authier, M., Boisseau, O., Bradai, M.N., … Vella, J. 2018. Assessing cetacean surveys throughout the Mediterranean Sea: A gap analysis in environmental space. Scientific Reports, 8(1): 3126.

[53] Bouchet, P.J., Miller, D.L., Roberts, J.J., Mannocci, L., Harris, C.M., Thomas, L. 2019. From here and now to there and then: Practical recommendations for extrapolating cetacean density surface models to novel conditions. Technical report 2019-01 v1.0, Centre for Research into Ecological & Environmental Modelling (CREEM), University of St Andrews, 59 p.

[54] Stoll, H., King, G., & Zeng, L. 2014. WhatIf: Software for evaluating counterfactuals. R package version 1.5-6. Retrieved from https:// cran.r-project.org/web/packages/WhatIf/index.html.

[55] Authier, M., Saraux, C., & Péron, C. 2016. Variable selection and accurate predictions in habitat modelling: A shrinkage approach. Ecography, 40: 549–560.

[56] King, G., & Zeng, L. 2007. When can history be our guide? The pitfalls of counterfactual inference. International Studies Quarterly, 51: 183–210.

[57] Putra, M.I.H., & Mustika, P.L.K. 2020. Incorporating in situ prey distribution into foraging habitat modelling for marine megafauna in the Solor waters of the Savu Sea, Indonesia. Aquatic Conservation: marine and freshwater ecosystems, 30(12): 2384-2401.

[58] Cañadas, A., de Soto, N.A., Aissi, M., Arcangeli, A., Azzolin, M., B-Nagy, A., … Roger, Th. 2018. The challenge of habitat modelling for threatened low density species using heterogeneous data: The case of Cuvier's beaked whales in the Mediterranean. Ecological Indicators, 85: 128–136.

[59] Mannocci, L., Roberts, J.J., Pedersen, E.J., & Halpin, P.N. 2020. Geographical differences in habitat relationships of cetaceans across an ocean basin. Ecography, 43:1-10

[60] Torres, L.G., Read, A.J., & Halpin, P. 2008. Fine-scale habitat modeling of a top marine predator: Do prey data improve predictive capacity. Ecological Applications, 18(7): 1702-1717.

[61] Watwood, S.L., Miller, P.J., Johnson, M., Madsen, P.T., & Tyack, P.L. 2006. Deep-diving foraging behaviour of sperm whales (*Physeter macrocephalus*). Journal of Animal Ecology, 75(3): 814-825.

[62] Madsen, P.T., de Soto, N.A., Tyack, P.L., & Johnson, M. 2014. Beaked whales. Current Biology, 24(16): 728–730.

[63] Pendleton, D.E., Holmes, E.E., Redfern, J., & Zhang, J. 2020. Using modelled prey to predict the distribution of a highly mobile marine mammal. Diversity and Distributions, 26(11): 1612-1626.

[64] Bost, C.A., Cotté, C., Bailleul, F., Cherel, Y., Charrassin, J.B., Guinet, C., … Weimerskirch, H. 2009. The importance of oceanographic fronts to marine birds and mammals of the southern oceans. Journal of Marine Systems, 78: 363–376.

[65] Woodson, C.B., & Litvin, S.Y. 2015. Ocean fronts drive marine fishery production and biogeochemical cycling. Proceedings of the National Academy of Sciences, 112(6): 1710–1715.

[66] Cañadas, A., Sagarminaga, R., De Stephanis, R., Urquiola, E., & Hammond, P.S. 2005. Habitat preference modelling as a conservation tool: Proposals for marine protected areas for cetaceans in southern Spanish waters. Aquatic Conservation: Marine and Freshwater Ecosystems, 15(5): 495–521.

[67] McShea, W.J. 2014. What are the roles of species distribution models in conservation planning? Environmental Conservation, 41: 93–96.

[68] Hazen, E.L., Palacios, D.M., Forney, K.A., Howell, E.A., Becker, E., Hoover, A.L., … & Bailey, H. 2017. WhaleWatch: A dynamic management tool for predicting blue whale density in the California Current. Journal of Applied Ecology, 54(5): 1415–1428.

[69] Fleishman, E., Nally, R.M., Fay, J.P., & Murphy, D.D. 2001. Modeling and predicting species occurrence using broad-scale environmental variables: An example with butterflies of the Great Basin. Conservation Biology, 15(6): 1674–1685.

[70] Lumaret, J.P., & Jay-Robert, P. 2002. Modelling the species richness distribution of French dung beetles (Coleoptera, Scarabaeidae) and delimiting the predictive capacity of different groups of explanatory variables. Global Ecology and Biogeography, 11(4): 265–277.

[71] Thaxter, C.B., Lascelles, B., Sugar, K., Cook, A.S., Roos, S., Bolton, M., … & Burton, N.H.K. 2012. Seabird foraging ranges as a preliminary tool for identifying candidate Marine Protected Areas. Biological Conservation, 156: 53–61.

[72] Young, H.S., Maxwell, S.M., Conners, M.G., & Shaffer, S.A. 2015. Pelagic marine protected areas protect foraging habitat for multiple breeding seabirds in the central Pacific. Biological Conservation, 181: 226–235.

Supporting information

Appendix S1: details of surveys used in the analyses. Total effort represents the total length of transects of each survey (without removing the transects with a Beaufort sea-state > 4). NE-ATL: Northeast Atlantic Ocean; NW-ATL: Northwest Atlantic Ocean.

Appendix S2: average conditions of the static, oceanographic and SEAPODYM variables over the entire period (from 1998 to 2015).

Appendix S3: correlations between environmental and SEAPODYM variables and model outputs for beaked and sperm whales.

Appendix S4: uncertainty maps representing the standard error associated with the predicted relative density of beaked (BW) and sperm (SW) whales. Black areas represent extrapolation where we did not extrapolate the predictions.

Figure 1

Click here to access/download;Figure;Figure1.tif

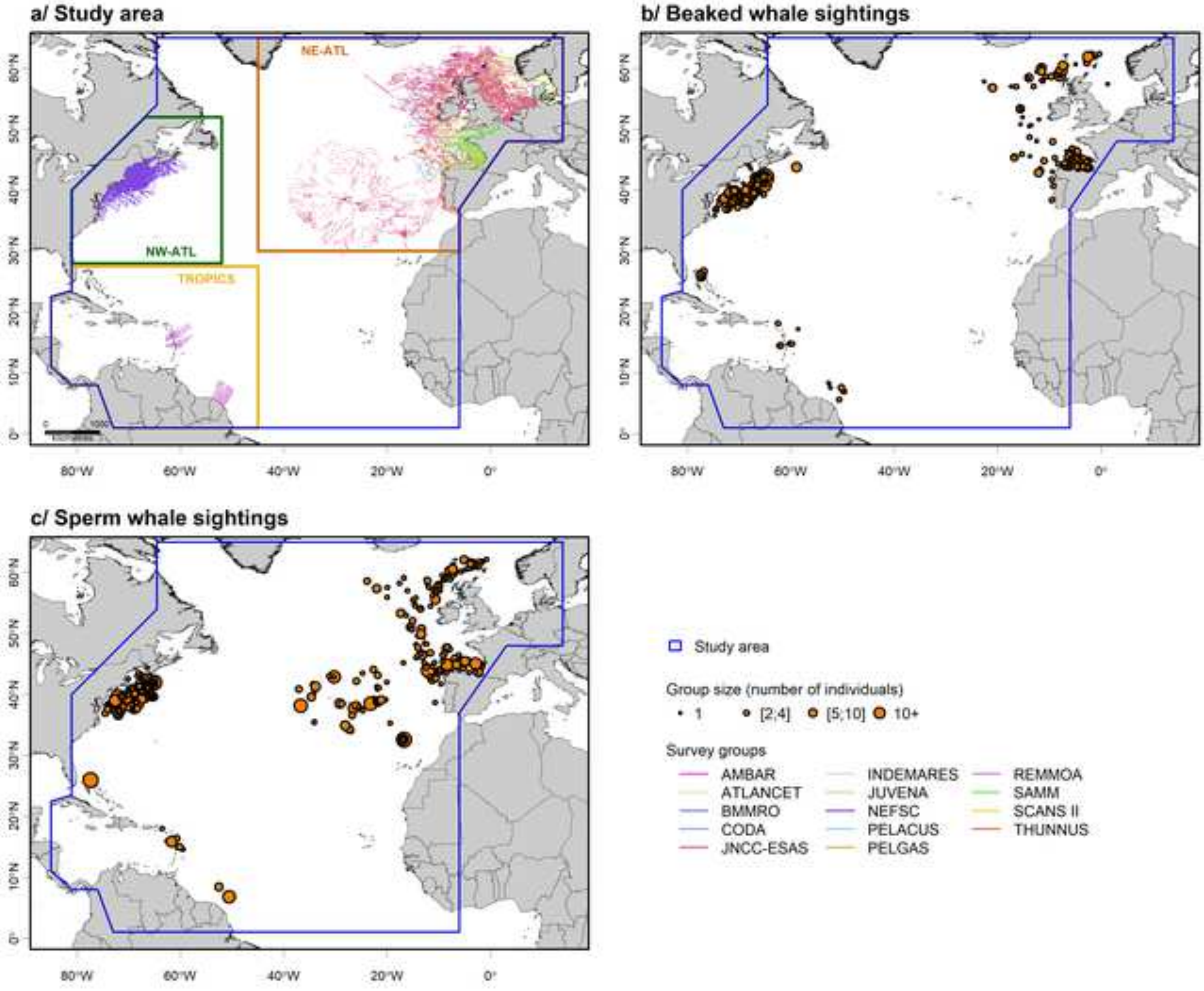

Figure 2

Click here to access/download;Figure;Figure2.png

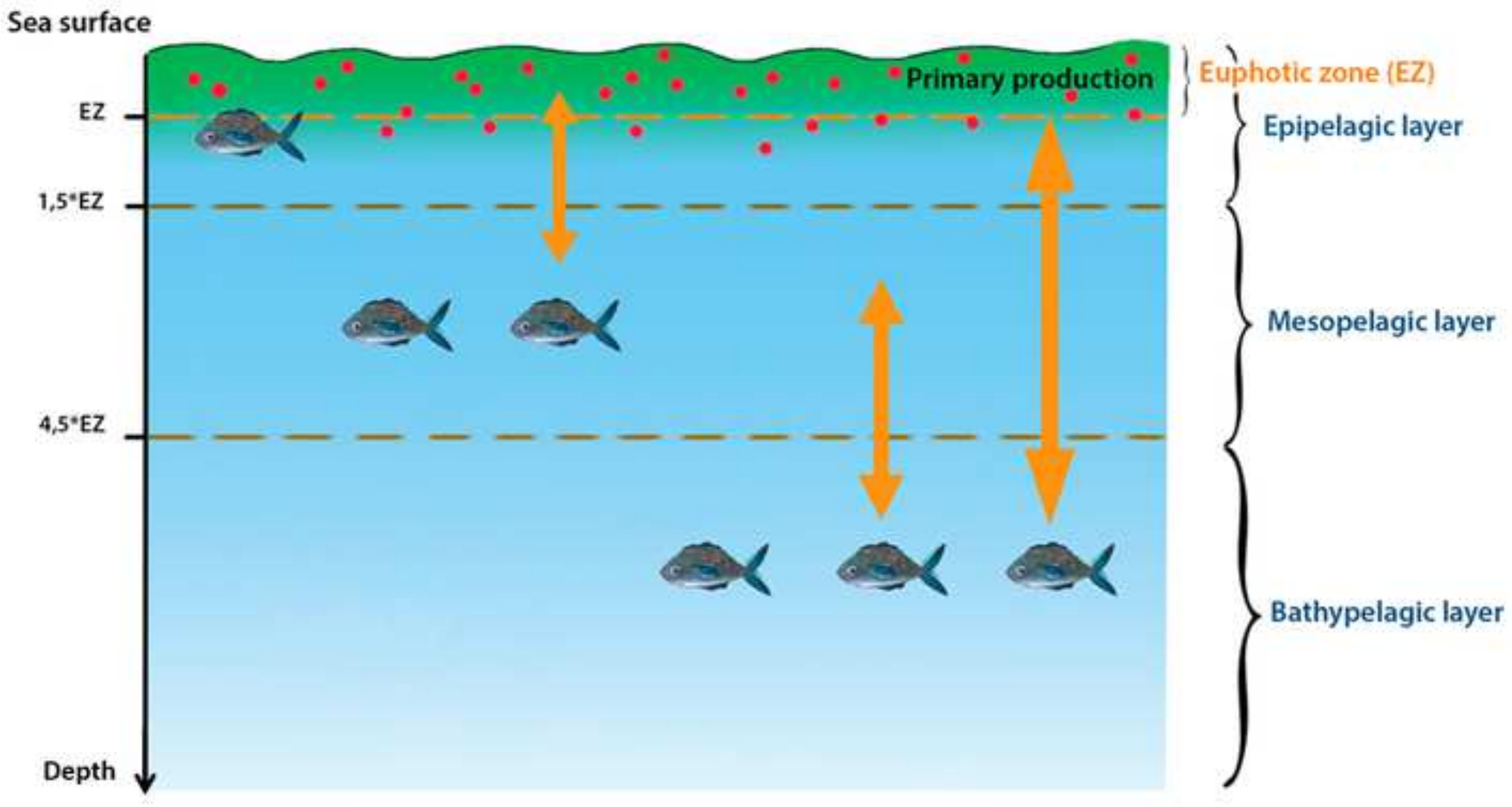

Figure 3

Click here to access/download;Figure;Figure3.png

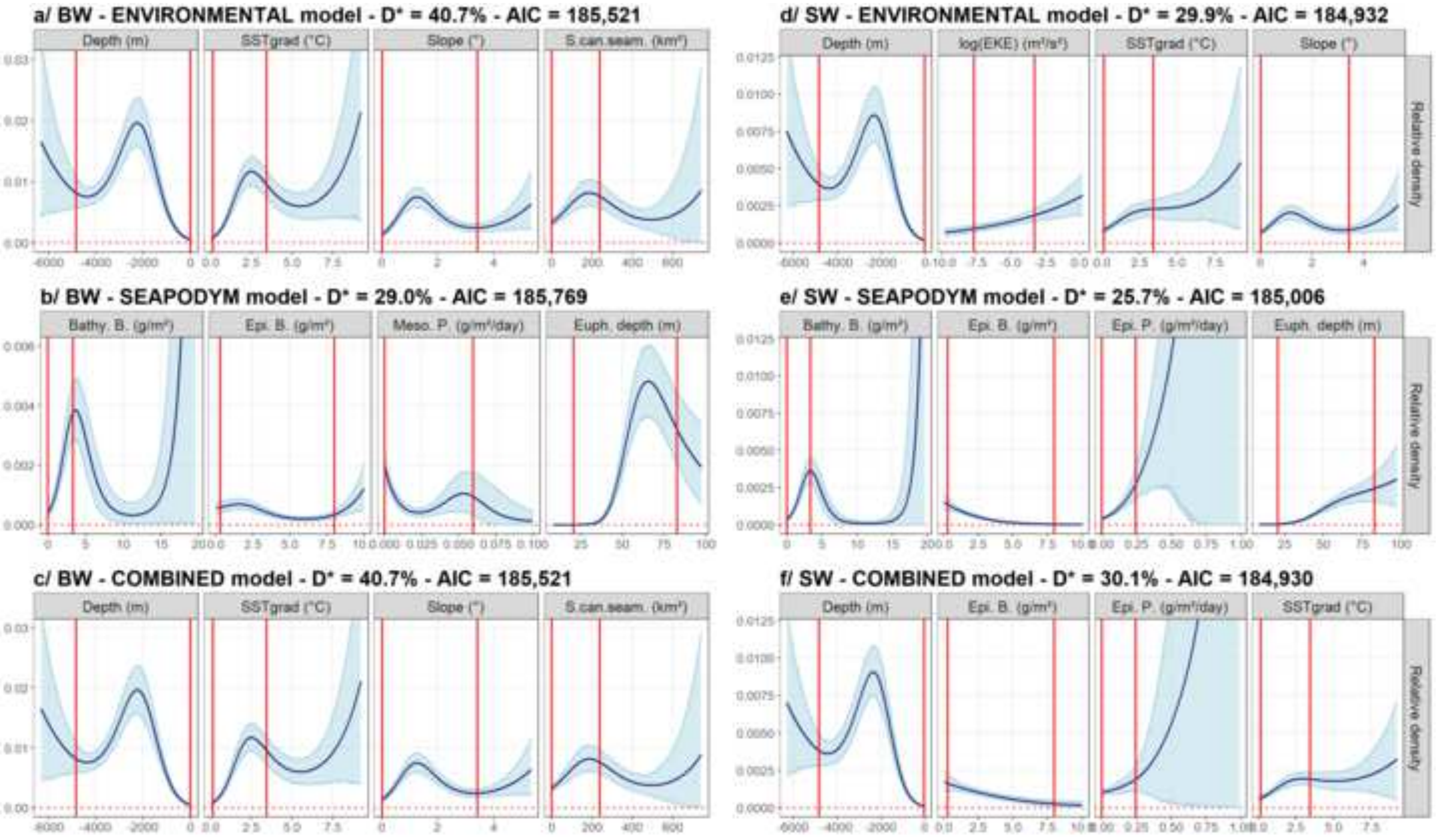

Figure 4

Click here to access/download;Figure;Figure4.png

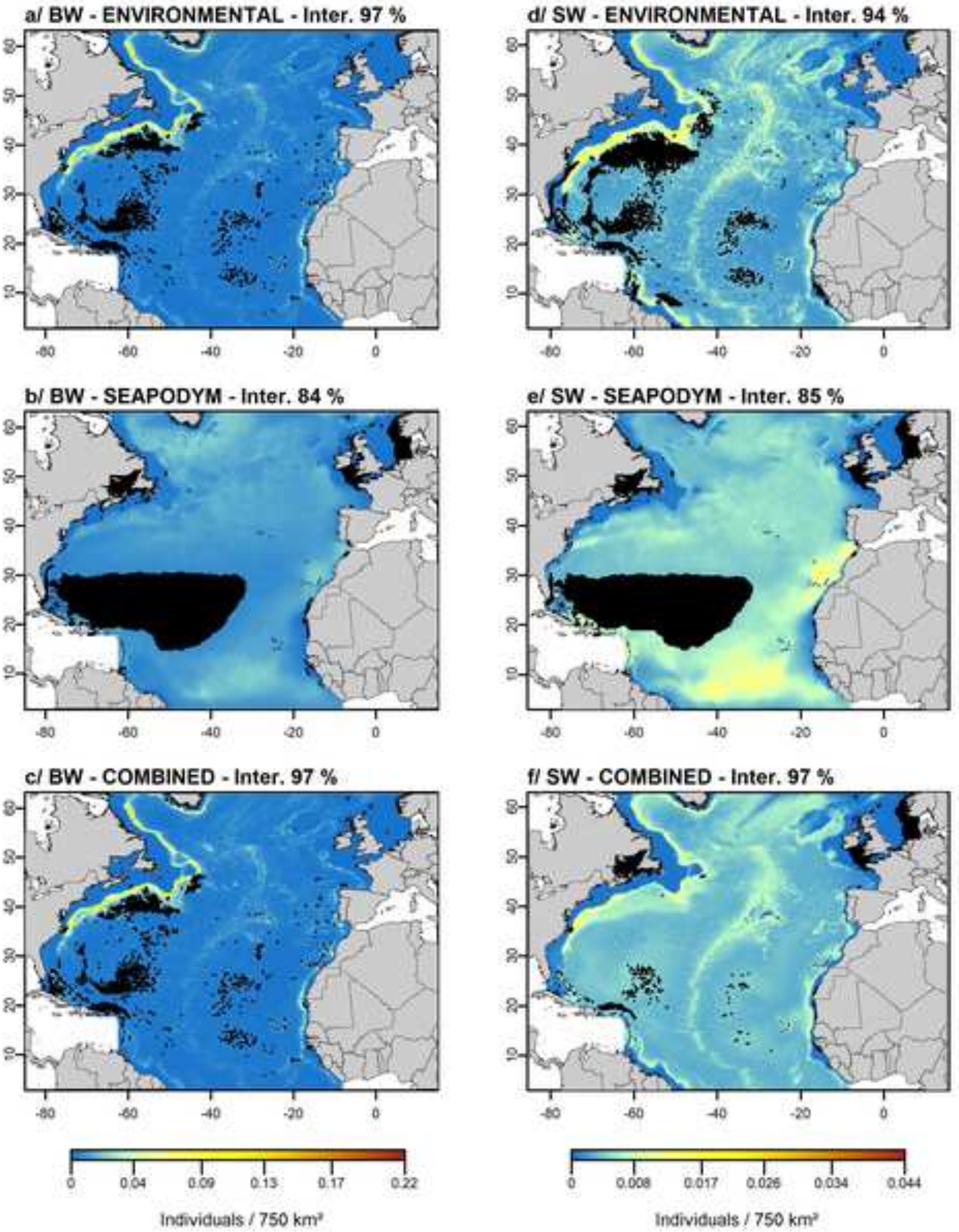

Click here to access/download
Supporting Information
AppendixS1.pdf

Supporting Information 2

Click here to access/download
Supporting Information
AppendixS2.pdf

Click here to access/download
Supporting Information
AppendixS3.pdf

Supporting Information 4

Click here to access/download

Supporting Information

AppendixS4.png
