# Supplementary material for: Towards a better characterisation of deep-diving whales’ distributions by using prey distribution model outputs?
Source: PLoS One. 2021 Aug 4;16(8):e0255667. doi: 10.1371/journal.pone.0255667 (PMC8336804; doi:10.1371/journal.pone.0255667)
Supplement: Attachment — Submitted filename: PONE-D-21-03978_R1_reviewer.pdf [file pone.0255667.s007.pdf]

PLOS ONE

Towards a better characterisation of deep-diving whales’ distributions by using prey distribution model outputs

--Manuscript Draft--

**Manuscript Number:**	PONE-D-21-03978R1
**Article Type:**	Research Article
**Full Title:**	Towards a better characterisation of deep-diving whales’ distributions by using prey distribution model outputs
**Short Title:**	Habitat modelling with prey distribution model outputs
**Corresponding Author:**	Auriane Virgili<br>La Rochelle University: Universite de La Rochelle<br>La Rochelle, FRANCE
**Keywords:**	Beaked whales; Habitat-based density modelling; SEAPODYM; Sperm whales; prey fields
**Abstract:**	In habitat modelling, environmental variables are assumed to be proxies of lower trophic levels distribution and by extension, of marine top predator distributions. More proximal variables, such as potential prey fields, could refine relationships between top predator distributions and their environment. In situ data on prey distributions are not available over large spatial scales but, a numerical model, the Spatial Ecosystem And POpulation DYnamics Model (SEAPODYM), provides simulations of the biomass and production of zooplankton and six functional groups of micronekton at the global scale. Here, we aimed at exploring if generalised additive models fitted to simulated prey distribution data better fit deep-diver densities (here beaked and sperm whales) than models fitted to environmental variables and testing whether the combination of environmental and prey distribution data would further improve model fit by comparing their explanatory power. For both taxa, we highlighted a preference for habitats associated with topographic features and thermal fronts but also for habitats where the euphotic zone is extended and with large lower mesopelagic prey. For beaked whales, combining variables did not improve the model performance, no SEAPDOYM variables were selected in the best model, possibly because they prey on organisms less well predicted by SEAPODYM. For sperm whales, by combining variables, we slightly increased the sperm whale model performance (higher explained deviance and lower root mean squared error). SEAPODYM outputs alone did not seem ideal for modelling prey of deep-diving cetaceans and maybe SEAPODYM was ruled out as a good framework for describing the species distribution, especially for beaked whales. However, SEAPODYM gives new insights about potential prey fields targeted by deep-divers at large spatial scales. Considering current SEAPODYM parameterisation, if predators feed on pelagic prey assemblages, the combined use of prey and environmental variables would represent an improvement through more accurate modelling of their densities.
**Order of Authors:**	Auriane Virgili
	Laura Hedon
	Matthieu Authier
	Beatriz Calmettes
	Diane Claridge
	Tim Cole
	Peter Corkeron
	Ghislain Dorémus
	Charlotte Dunn
	Tim E Dunn
	Sophie Laran

Powered by Editorial Manager® and ProduXion Manager® from Aries Systems Corporation

	Patrick Lehodey
	Mark Lewis
	Maite Louzao
	Laura Mannocci
	José Martinez-Cedeira
	Pascal Monestiez
	Debra Palka
	Emeline Pettex
	Jason J Roberts
	Leire Ruiz
	Camilo Saavedra
	M. Begoña Santos
	Olivier Van Canneyt
	José Antonio Vázquez Bonales
	Vincent Ridoux
**Response to Reviewers:**	Dear Editor,<br><br>Thank you for considering our article entitled “Towards a better characterisation of deep-diving whales’ distributions by using prey distribution model outputs”, for publication in the journal PLOS ONE. We have taken into account all comments from the editor and the three reviewers regarding the article.<br><br>We thank the reviewers for their careful reading of our manuscript and for pointing out elements in the abstract, methods and discussion that needed to be more developed. We did not change the models used in our study, as generalized additive models have repeatedly shown their effectiveness in modelling the distribution of cetaceans (e.g. Roberts et al., 2016; Becker et al., 2017; Cañadas et al., 2018; Virgili et al., 2019) but we have completed the methodology to make it more robust, we have changed the temporal resolution of the SEAPODYM variables so that the link between species distribution and these variables would be more direct, we have modified the process of variable selection for the COMBINED models, and we have added a new parameter for estimating the model performance. Many discussion points have been completed and added in order to meet the expectations of the reviewers.<br><br>We have fixed all typos and grammatical mistakes during the revision and ensured that the manuscript meets the PLOS ONE’s style requirements.<br><br>We hope that the revised manuscript addresses the different concerns raised by the editor and the reviewers, whom we thank for their careful reading and review of this manuscript. We feel the revised manuscript has improved as a result and hope it will be deemed acceptable for consideration of publication in PLOS ONE.<br><br>Sincerely,<br><br>On behalf of all co-authors,<br><br>Auriane Virgili<br><br>Concerning specific Editor and reviewer’s comments:<br><br>Editor’s comments:<br><br>1. Please ensure that your manuscript meets PLOS ONE's style requirements, including those for file naming.

ANSWER: We have checked all the requirements provided on the website and modified the manuscript to meet these requirements.

2. Thank you for stating the following in the Competing Interests section:
'The authors have declared that no competing interests exist.'
We note that one or more of the authors are employed by a commercial company: ADERA
a. Please provide an amended Funding Statement declaring this commercial affiliation, as well as a statement regarding the Role of Funders in your study. If the funding organization did not play a role in the study design, data collection and analysis, decision to publish, or preparation of the manuscript and only provided financial support in the form of authors' salaries and/or research materials, please review your statements relating to the author contributions, and ensure you have specifically and accurately indicated the role(s) that these authors had in your study. You can update author roles in the Author Contributions section of the online submission form.
Please also include the following statement within your amended Funding Statement.
“The funder provided support in the form of salaries for authors [insert relevant initials], but did not have any additional role in the study design, data collection and analysis, decision to publish, or preparation of the manuscript. The specific roles of these authors are articulated in the ‘author contributions’ section.”
If your commercial affiliation did play a role in your study, please state and explain this role within your updated Funding Statement.
b. Please also provide an updated Competing Interests Statement declaring this commercial affiliation along with any other relevant declarations relating to employment, consultancy, patents, products in development, or marketed products, etc.
Within your Competing Interests Statement, please confirm that this commercial affiliation does not alter your adherence to all PLOS ONE policies on sharing data and materials by including the following statement: "This does not alter our adherence to PLOS ONE policies on sharing data and materials.” (as detailed online in our guide for authors http://journals.plos.org/plosone/s/competing-interests). If this adherence statement is not accurate and there are restrictions on sharing of data and/or materials, please state these. Please note that we cannot proceed with consideration of your article until this information has been declared.
c. Please include both an updated Funding Statement and Competing Interests Statement in your cover letter. We will change the online submission form on your behalf.
Please know it is PLOS ONE policy for corresponding authors to declare, on behalf of all authors, all potential competing interests for the purposes of transparency. PLOS defines a competing interest as anything that interferes with, or could reasonably be perceived as interfering with, the full and objective presentation, peer review, editorial decision-making, or publication of research or non-research articles submitted to one of the journals. Competing interests can be financial or non-financial, professional, or personal. Competing interests can arise in relationship to an organization or another person. Please follow this link to our website for more details on competing interests: http://journals.plos.org/plosone/s/competing-interests.

ANSWER:
Funding statement
AV’s doctoral research grant was funded by the Direction Générale de l'Armement (DGA).
ML was funded by a Ramón y Cajal (RYC-2012-09897) postdoctoral contract of the Spanish Ministry of Economy, Industry and Competitiveness. This study is a contribution to the CHALLENGES (CTM2013-47032-R) project of the Spanish Ministry of Economy, Industry and Competitiveness.
MA and EP are affiliated to ADERA a private business dedicated to providing support for the management of research project in public institutions in Region Nouvelle Aquitaine. In this study, ADERA solely provided support in the form of salaries for authors MA and EP, but did not have any additional role in the study design, data collection and analysis, decision to publish, or preparation of the manuscript. The specific roles of these authors are articulated in the ‘author contributions’ section.

Competing interest statement
MA and EP are employed by the commercial company ADERA which did not play any

role in this study beyond that of employer. This does not alter our adherence to PLOS ONE policies on sharing data and materials.

3. We note that you have indicated that data from this study are available upon request. PLOS only allows data to be available upon request if there are legal or ethical restrictions on sharing data publicly. For more information on unacceptable data access restrictions, please see http://journals.plos.org/plosone/s/data-availability#loc-unacceptable-data-access-restrictions.

ANSWER: We indicated the data from this study are available upon request because each of the co-authors of the manuscript owns part of the dataset we used and a request has been made to each of them to have access to the data so we cannot provide the dataset without their agreement. All sighting and effort data used in this study are available in the OBIS SEAMAP database: http://seamap.env.duke.edu/. Authors had no special access to the underlying data and all interested researchers can obtain the data from http://seamap.env.duke.edu in the same way that the authors obtained data by contacting the data providers via the OBIS SEAMAP website.

Email address of organisations/data providers:
AMBAR: ambarelkartea@gmail.com
AZTI: mlouzao@azti.es
BMMRO: dclaridge@bahamaswhales.org
CEMMA: cemmaorganizacion@gmail.com
IEO: Begona.SANTOS@efca.europa.eu
JNCC: tim.dunn@jncc.gov.uk
NEFSC: jason.roberts@duke.edu
PELAGIS: pelagis@univ-lr.fr
SEAPODYM: plehodey@groupcls.com
SMRU: psh2@st-andrews.ac.uk

4. We note that Appendix S2, S4 and Figures 1 and 4 in your submission contain map/satellite images which may be copyrighted.

ANSWER: All captions and figures were completed with the source of the base map (https://www.gebco.net/).

Reviewer 1 #

The article entitled "Towards a better characterisation 1 of deep-diving whales' distributions by using prey distribution model outputs" aimed at comparing 2 habitat modelling approaches to predict the distribution of deep divers using surface environmental variables vs. low and mid-trophic levels outputs. The authors showed that including SEAPODYM covariates does not improve the model performance and conversely lead to drastic different predictions, raising serious concern regarding the reliability of the model output and the algorithm used (classical additive model). Despite the efforts of the authors to provide a clear methodology, the results lack of clarity (numerous repetitions, the results are not sufficiently discussed in regards to other studies) and the bias / limitations of the modelling procedure are not well discussed. I suggested several improvements, including testing other algorithms from machine learning and adding other covariates into the model as well as additional literature. The authors have a dataset will a great potential, and I'm convinced once the methodology will be made more robust and the results clearer, this article will be of great contribution to the habitat modelling and marine mammals' communities!

ANSWER: We thank reviewer 1 for his careful reading of our manuscript and for pointing out methodological and redactional concerns. The methodology we used for the selection of variables in the models has been revised in the new version of the manuscript and a new metric has been used to evaluate the model performance in order to better meet the objectives of the study but we have not tested other algorithms as suggested. Indeed, generalized additive models have repeatedly shown their effectiveness to model cetacean distributions (e.g. Roberts et al., 2016; Becker et al., 2017; Cañadas et al., 2018; Virgili et al., 2019) and we believe they can also be applied in this study. Reviewer 1 proposes to use machine learning algorithms instead which is justified if the main objective is to predict the distribution of species without

necessarily explaining this distribution (Gobeyn et al., 2019) but here our objective was primarily to determine the explanatory performance of models that use different types of explanatory variables. It therefore seemed more relevant to use statistical models rather than machine learning. In addition, surface environmental variables are commonly used in generalized additive models to model the distribution of deep-divers and we wanted to assess whether the addition of prey distribution variables provided additional information to explain their distribution. Therefore, a comparable modelling approach was required. In our future studies, we will consider machine learning algorithms to predict the distribution of species. We have clarified the results and removed repetitions to meet reviewer's expectation (lines 304-366). We have more discussed bias and limitations of our study (lines 382-430, 549-557) and added more literature to discuss our study. We think the revised manuscript has improved as a result.

Specific comments:

Page 2: This result is surprising and not very intuitive. look forward reading some explanations about this; same. isn't it the opposite?; I am wondering how these prey field variables are not strongly autocorrelated, but I presume you have tested that?; more than a description of the most significant variables selected for each model, I would have expected some performance metrics (e.g. deviance explained) to compare the 3 models. I would have been much more informative and less descriptive to have such results in the abstract

ANSWER: We have changed the temporal resolution of the SEAPODYM variables (from 29 to 7 days) and fitted new models so the results have changed in this new version of the manuscript and we have reworked the abstract as a whole, which we hope will address the comments of reviewer 1.

Line 66: predictors? to avoid repetition

ANSWER: Changed

Line 77: also because such micronekton outputs are closer to trophic level of apex predators prey

ANSWER: Complement added in the text lines 77-78.

Line 86: you could also add this reference which shows the use of SEAPODYM to relate olive ridleys turtles to productive eddies: https://www.sciencedirect.com/science/article/abs/pii/S0079661115300094

ANSWER: Reference added line 87.

Lines 96-97: no need here to describe all the predictors you plan to use

ANSWER: Text deleted.

Line 129: the resolution of the figures is very low. I assume it is due to the requested format by the journal, but figures in vector format such as pdf would be needed to get a better result

ANSWER: Figures were provided in a format requested by the journal; the poor resolution is probably due to the creation of the pdf submission.

Line 159: dynamic or oceanographic? to be consistent pick one of these 2

ANSWER: We have changed dynamic into oceanographic in the whole text.

Line 172: you could have done the same procedure with SSH to detect ocean fronts and particularly eddies. SSH and SST fronts can be slightly different and might provide complementary variables. See Chambault et al 2017 DSR II for more information.

ANSWER: We thank the reviewer for this proposal, indeed, it is a variable that could be

interesting. To our knowledge, this variable is not known to have an effect on the deep-diver distribution so we have chosen not to include it in the new version of the manuscript, we have limited ourselves to classically used variables but we will test this variable in our future studies.

Line 174: to be consistent I would suggest to use the same products as the ones assimilated into SEAPODYM. I might be wrong but I think SEAPODYM assimilates SST and currents from CMEMS directly (GLORYS product). If this is the case I would recommend to use products from CMEMS rather than many different sources of data

ANSWER: We do not believe that it makes sense to use the same data source for the environmental and SEAPODYM variables as this will necessarily lead to a correlation between the variables and therefore we would not be able to obtain a combination of the environmental and SEAPODYM variables in the COMBINED models.

Line 176: from AVISO there is also a useful product assembling all the eddies with their associated characteristics (radius, type, amplitude...): it is called the eddy Atlas. It could provide another predictor, especially since your whales seem to target the Gulf Stream

ANSWER: We thank reviewer 1 for this information but we do not think this type of variable at a very fine spatial resolution is very relevant for visual observation data, it would be more relevant for tracking data and therefore would not be applicable here.

Line 178: another dynamic variable that could be of great interest for such diving predators is the bottom temperature available on CMEMS. for these animals that dive to great depths for long periods it makes more sense to also look at vertical variables like bottom temp

ANSWER: We fully agree with reviewer 1 and the use of depth variables will be the subject of another publication. Here, the objective was to compare the SEAPODYM variables to environmental variables classically used in habitat models such as static variables and surface variables that was why we did not include bottom temperature in the models.

Line 242: you should also test the correlation using the vif function from the usdm package. the common way is to retain all variables with a vif coefficient < 4

ANSWER: As suggested by reviewer 1, we have tested the correlation with the VIF function, the results were almost identical as the correlation matrices calculated with this method and our method were identical within 0.001. In addition, our method allowed to test all combinations of variables without first selecting one variable among two correlated variables, contrary to the VIF function, which seemed more relevant, so we have kept our variable selection method.

Line 243: I think 4 is quite a low number of variables, and you therefore risk to strongly limit the performance of your model. In SDM, many studies commonly us more than 4 predictors, so I suggest you don't limit the number of variables and simply remove the ones that are not significant

ANSWER: We understand reviewer's concern, we have constrained the number of variables in the models to a maximum of 4 (ensuring that they were all significant) to avoid excessive complexity of models and difficulty in their interpretation. Although this could limit the performance of our models, we were confident in the procedure parsimony. Indeed, the explained deviances obtained (between 20 and 40%) were high compared to other studies on cetaceans, suggestive of a good fit to the data, and for each model, only four or five variables were actually important, the others representing a very small percentage of the Akaike weights (S3.2 Fig. in Supplementary material 3). As a result, four variable models were the best compromise between avoidance of overfitting, reduction of complexity and maximisation of interpretability. We have added this paragraph lines 420-430 to discuss methodology we have employed.

Line 245: I am very surprised by this result! especially because you consider for each

Powered by Editorial Manager® and ProduXion Manager® from Aries Systems Corporation

layer both the biomass and production. could you please test with the vif function and see if it is still not correlated?

ANSWER: We have checked the correlations between the variables with our method and the VIF function and we confirm these variables were not correlated (see S3.1 Fig. in Supplementary material 3).

Line 263: have you included the month and year as random effect or explanatory variable then?

ANSWER: We did not include month and year as random effect or explanatory variables in the models because as we discuss lines 408-519, “One of the problems faced when studying the distribution of deep-diving cetaceans through habitat modelling is the lack of sighting data, it is necessary to assemble datasets from different surveys to obtain sufficient data to fit habitat models. Sampling is therefore non-uniform, both spatially and temporally, with some areas, years or seasons being much more represented. For example, some years were only sampled in the northwest Atlantic area. Therefore, we chose to combine all the data to ensure we had enough data without considering a temporal effect in the models. However, temporality was taken into account when associating environmental conditions with effort segments, so when there was a sighting, it was associated with environmental conditions averaged over the week for the SEAPODYM variables and over the month for the environmental variables before the sighting, to take into account potential time-lags. When deep-diver data will be large enough to allow a more uniform temporal sampling, the temporal aspect will be taken into account in the models.”.

Line 296: to be more logical and consistent with the text I suggest to convert the bathymetry in absolute values the values on both axes are not readable the Y axis should not be in log?

ANSWER: As suggested by reviewer 1, we have changed the scale of the bathymetry in Figure 3. The Y axis is a relative density in individuals/750 km² so it has not to be in log.

Line 302: I still have a big doubt about that. Thanks for sharing the correlation matrix though, I'll have a look at it, but I think running the vif is strongly recommended here

ANSWER: VIF also showed no correlation between the variables as shown in our correlation matrix in S3.1 Fig.

Line 302: when reading Fig 3b it is the opposite, and much more logical indeed! maybe I missed sth here but your graph shows a positive relationship btw bathy biomass and whale's density

ANSWER: Results have changed in the new version of the manuscript because the temporal resolution of SEAPODYM variables have changed so we have modified the text here.

Line 303: however, relationships with epi B and meso B seem very unclear. were these variables significant or you decided to retain 4 variables even if not significant? if you did so, I think this is not the proper way to do it as these 2 predictors seem to have a very little influence on your response variable

ANSWER: All variables selected in the final models were significant but may have less influence compared to other which may have a great influence, we do not believe the method should be questioned here.

Line 304: in your legend you mention 2.5 to 97.5 percentile. Please be consistent

ANSWER: Reviewer 1 is absolutely right, it is a mistake on our part, we have made the necessary changes in the text.

Line 306: I don't really see that on your Fig 4b, probably due to the legend min max values. I suggest you adjust the colorbar to the values of each model, otherwise it is

very hard to see any favourable habitat here

ANSWER: We agree the colorbar may not be fully optimised but we chose to apply the same colorbar to facilitate the comparison between the different models, on the advice of many colleagues.

Line 319: I don't understand how your 97.5% percentile can be so tight towards the left-hand side for instance in b) bathy B? it means you have only very few data beyond 4?

ANSWER: We thank reviewer 1 for pointing out this mistake, we have modified the figure.

Line 336: weird because you had no data on such a habitat. likely due to the influence of the slope but it raises serious concern about your model performance to predict outside they are of the training dataset. this needs to be discussed

ANSWER: We understand reviewer's concern and we discuss this on lines 549-557 "We performed a gap analysis to highlight geographical extrapolation areas in which environmental and SEAPODYM variables were out ranges of surveyed conditions. The centre of the study area, near the Mid-Atlantic Ridge, was not surveyed but variable conditions remained within the ranges of surveyed conditions, allowing to predict distribution at a large scale. The gap analysis revealed large gaps in environmental space coverage across the study area, especially in the western part of the Atlantic Ocean for the ENVIRONMENTAL models and in tropical waters for the SEAPODYM models. This suggests that sampling effort was not sufficient in deeper and steeper areas and more intensive sampling effort performed in these areas could help better describe the habitat used by deep-divers."

Line 338: again I disagree with your interpretation here. if you consider the values between the 5 and 95% percentile, the highest densities are found for high values of Bathy B and Epi P, not the opposite

ANSWER: The results have changed here and so the text, we hope our new interpretations agree with reviewer's interpretations.

Line 342: the strong dissimilarities between the ENV and SEAPODYM predictions raise serious concerns regarding the reliability of your modelling approach

ANSWER: The dissimilarities between ENVIRONMENTAL and SEAPODYM predictions mainly raised the effect of the variables and this is likely due to the spatial resolution of the SEAPODYM variables as discussed lines 540-545 "Beaked and sperm whales seemed homogeneously distributed in oceanic waters, which was not very consistent with the sightings and highlighted the poorer performance of the models (higher RMSEs). As previously showed in Lambert et al. (2014), these smoother predictions may be due to the large resolution of the variables. The spatial resolution of the SEAPODYM variables is currently set to 0.25° but is planned to be refined to 0.125° and a finer spatial resolution may reduce this smooth effect.".

Lines 364-368: already said earlier, not necessary here

ANSWER: Text removed.

Lines 373-375: true but such approach could partly explain your relatively low deviances and such differences between your models for each species. For example, sperm whales in the Caribbean might adopt a totally different habitat selection compare to their conspecific in the North-East Atlantic, simply due to genetic differentiation or habitat available

ANSWER: We agree with reviewer 1, deep-divers may have different responses between north and northwest Atlantic and we discussed this on lines 394-407. However, explained deviances were not low compare to other studies that use visual observation data (Becker et al., 2017; Cañadas et al., 2018), which suggested a good fit to the data.

Line 385: another explanation could be related to the spatial resolution of your data. 0.25 degrees is quite coarse to depict specific associations between an animal and its environment. I have heard the SEAPODYM might be soon available daily at 0.08 degrees, so I think it is worth mentioning such limitation

ANSWER: We agree with reviewer 1 and we have added this on lines 443-444.

Line 387: 0.2% difference is very negligible to me so this is not a strong statement. For both species, the SEAPODYM model was less performant, so I don't see the point to include such mid-trophic level covariates

ANSWER: With the change in temporal resolution of the SEAPODYM variable, results have changed and the difference was less negligible but we agree with reviewer 1 that the SEAPODYM models were less performant than ENVIRONMENTAL and COMBINED models.

Line 389: it would be interesting to incorporate more variables into each model and not limit it to 4 to see if it changes the results. if your SEAPODYM covariates appear to be important but less important than the 4 first other variables according to you AIC, it could be the reason why you did not find any SEAPODYM variables in the combined model for beaked whales. not sure it'll change the story but it is worth trying it

ANSWER: We agree with the reviewer's comment but as shown in S3.2 Fig in Supplementary material 3, no SEAPODYM variables were important in the beaked whale COMBINED model and only production of epipelagic organisms was an important variable in the sperm whale COMBINED model so even if the number of variables was higher, SEAPODYM variables probably would not have been selected in the models or they would not have been significant.

Line 393: ok but the slope did not appear to be drive a lot beaked whale distribution neither... probably due to the coarse 0.25 ° resolution you used. A similar study predicting sperm whale distribution based on tracking data faced the same issue with the slope when incorporating it into the model:
https://onlinelibrary.wiley.com/doi/full/10.1002/ece3.7154

ANSWER: We do not understand the reviewer's comment here because slope appeared to be an important variable for beaked whales as shown in Fig 3 or in S3.2 Fig in Supplementary material 3.

Line 395: there could also be a temporal mismatch (temporal lag) between SEAPODYM output and the growth rate of the actual prey of these species. This issue was discussed in Chambault et al 2017, DSR

ANSWER: We agree with reviewer's comment and we have added this point on lines 456-457.

Page 17-18:

ANSWER: As suggested by reviewer 1, we have removed repetitions on these two pages and the results have changed in the new version do it is difficult to answer to the specific comments written on these two pages.

Line 449: that's why I suggested to add the bottom temperature as another covariate. such variable was found to play a key role in sperm whale distribution around Mauritius: Chambault et al 2021, Ecol Evol

ANSWER: We fully agree and this will be done in another publication.

Line 451: in theory maybe, but your results show the opposite! also sperm whales can forage on the bottom of the ocean and not necessarily conduct pelagic dives so you need to be careful with your statement:
https://onlinelibrary.wiley.com/doi/full/10.1002/ece3.3322

Powered by Editorial Manager® and ProduXion Manager® from Aries Systems Corporation

ANSWER: We do not understand why our results show the opposite, the slope was not selected in the COMBINED model (Fig S3) and was not considered as important as shown in S3.2 Fig in Supplementary material, sperm whales are likely to feed at the bottom as well, but perhaps less consistently than beaked whales, which performed deeper dives in average.

Line 453: more than the euphotic depth, you should look at the mixed layer depth as well. for deep divers the light indication given by the Zeu might not be very informative while the MLD will provide information on the depth of the thermocline which is frequently used by diving animals. this variable can be easily found on Copernicus as well

ANSWER: We agree with reviewer 1; the mixed layer depth would probably be more informative for deep-divers because it provides information on the depth of the thermocline which is frequently used by diving animals and we will explore this in future studies. Here we used euphotic depth because it is a variable available in SEAPODYM but mixed layer depth would probably have been more relevant. We discuss this on lines 515-521.

Line 461: repetition!

ANSWER: Text removed.

Line 467: for animals that display a coastal pattern, like bowhead whales, bathymetry is indeed important. this is a general rule for all coastal animals while pelagic ones will describe more affinities for oceanographic structures like eddies, fronts. a similar relationship was found in narwhals which strongly use glaciers and fjords:
https://link.springer.com/article/10.1007/s00300-018-2345-y

ANSWER: We agree with reviewer 1 and it is probably true for sperm whales but according to literature, beaked whales seem particularly linked to bathymetry and physiographic structures like canyons and seamounts.

Line 471: I strongly disagree! the maps were not similar at all between the ENV and SEAPODYM models. You can say that for beaked whales they were quite the same for the ENV and COMBINED models, but this is it

ANSWER: We have changed the text on lines 532-548 and better interpreted our results, we agree, it was poorly worded.

Line 471: I would say totally different and you need to discuss that because it raises serious concern regarding your model performance and reliability. the choice of the algorithm might not be adequate. Machine learning algorithm offer a wide range of algo depicting complex relationships and might be appropriate and easy to implement on your data. Here, are few exmaples of recent studies involving machine learning in SDM:
Mannocci et al 2021
Chambault et al 2021, Ecol Evol
https://www.sciencedirect.com/science/article/abs/pii/S0304380018304010
https://www.koreascience.or.kr/article/JAKO201216636292901.page
https://link.springer.com/chapter/10.1007/978-1-4419-7390-0_8

ANSWER: We thank reviewer 1 for raising this point. As previously said, machine learning algorithms are justified if the main objective is to predict the distribution of species without necessarily explaining this distribution (Gobeyn et al., 2019) but here our objective was primarily to determine the explanatory performance of models that use different types of explanatory variables. It therefore seemed more relevant to use statistical models rather than machine learning. In addition, surface environmental variables are commonly used in generalized additive models to model the distribution of deep-divers and we wanted to assess whether the addition of prey distribution variables provided additional information to explain their distribution. Therefore, a comparable modelling approach was required. In our future studies, we will consider machine learning algorithms to predict the distribution of species.

Page 20:

ANSWER: All the conclusion has been modified, we hope it will meet the reviewer's expectations.

Reviewer 2 #

Virgili et al. have identified an important gap in our understanding of the link between potential habitat for deep-diving marine predators and the distribution of those predators- namely the influence of prey on the distribution of predators as an intermediate explanatory step between the environment and predators. The most important finding of this work is that the SEAPODYM model of pelagic fish distribution does not explain the distribution of deep-diving predators better than environmental data (except for some weak evidence for sperm whales).
While the science in the paper is basically sound (though see comment below regarding model derivation), the framing needs a bit of work before it should be published. That is, what is the driving hypothesis of this paper? There seem to be two possible frameworks:
1) The current hypothesis is that SEAPODYM variables better explain the distribution of beaked and sperm whales. This was found not to be the case (that is, this is a negative result). If this framework is preferred, it should be stated in the abstract, introduction and conclusion very clearly that SEAPODYM was ruled out as a good framework for describing the distribution of these deep divers

ANSWER: We thank the reviewer 2 for pointing out that the framework was not well enough defined, we fully agree. We have therefore modified the abstract (lines 46-48 " SEAPODYM outputs alone did not seem ideal for modelling prey of deep-diving cetaceans and maybe SEAPODYM was ruled out as a good framework for describing the species distribution, especially for beaked whales."), the introduction (lines 104-109 "We hypothesised that SEAPODYM variables would better explain the distribution of beaked whales and sperm whales because they are more proximal variables and because they characterise deep layers in which deep-divers feed. We also expected a better explanatory power of the model that combined the two types of variables because it considered static variables known to influence the beaked and sperm whale distribution, together with prey distribution data, presumably better suited to describe the species distributions.") and the whole conclusion (lines 577-593) to clarify the framework.

2) Given that, as stated in the discussion lines 396-424, SEAPODYM variables attempt to predict pelagic fish concentrations shallower than 1000m (that is, not the preferred prey of the study predators), a better test of SEAPODYM would be to choose a different predator to examine. Perhaps tuna or some other publicly available dataset (see e.g. https://portal.atn.ioos.us/) could be used to test the model more appropriately?

ANSWER: As stated in the discussion (lines 460-465), deep-divers can feed on prey larger than 20 cm at depths greater than 1,000 m and we agree we reviewer 2 that SEAPODYM variables would probably better explain the distribution of predator species such as tuna (which has been done). However, as stated in the introduction (lines 90-92 "Deep-divers are species of interest because they are sensitive to underwater noise pollution and an accurate knowledge of their distribution is crucial to mitigate the impact of human activities.") and discussion (lines 374-378 "The models were fitted to sightings of deep-diving cetaceans (beaked and sperm whales) because their habitat use is still poorly known and they face important anthropogenic threats including activities producing high intensity noise (e.g. military sonars or seismic guns), which makes them species of major interest."), it is important to better understand the distribution of deep-divers as their distribution is poorly known and they are species sensitive to underwater noise pollution (e.g. active military sonars) so we need to identify new methods that could allow a better understanding of their habitats. The method with the SEAPODYM variables does not seem to be ideal for this according to our study but it allows us to progress in the process.

Besides framing, the other major issue with the manuscript in its current form is that it

Powered by Editorial Manager® and ProduXion Manager® from Aries Systems Corporation

does not appear that the SEAPODYM model has fundamentally different information than the ENVIRONMENTAL model, thus it is not surprising that it does not improve the explanatory power of the environmental model. That is, in the paragraph on line 160, the SEAPODYM model appears to derive from SST, bathymetric and surface current data, which is the same information that parameterizes the ENVIRONMENTAL model. Is not one then just a transformation of the other? What information does the SEAPODYM model have the that the environmental model does not. There may be something in how data was integrated over time, but it was not clear from the current manuscript how these models are substantially different. In the revision, please clarify how these models provide different information, given that they do not actually include information about prey in the SEAPODYM model.

ANSWER: We fully agree with reviewer 2, the description of the SEAPODYM variables was not complete enough and it was difficult to understand how these variables provide different information from environmental variables. We have therefore modified the section on SEAPODYM variables (lines 187-240) and we hope that the difference is clearer this time. SEAPODYM is a numerical model used to compute the spatial distribution of the biomass and the production of the micronekton and the zooplankton, in different layers of the water column. Energy transfers from the primary production to the groups of micronekton are parameterised in the model and a system of advection–diffusion–reaction equations, that take into account the vertical migrations of organisms, are used to model recruitment, ageing, mortality, and passive transport with horizontal currents. SEAPODYM makes it possible to obtain, in each cell of a grid, an estimate of the total quantity of organisms of each functional group present in each layer and of the productivity of the functional group in each layer.

Minor comments:

Abstract: please include a stronger summary in the abstract describing the main results (that SEAPODYM is not a good predictor of deep-diving cetacean habitat).

ANSWER: The part of the abstract concerning the results has been completely modified to better define the results we have obtained (lines 42-49 “For beaked whales, combining variables did not improve the model performance, no SEAPDOYM variables were selected in the best model, possibly because they prey on organisms less well predicted by SEAPODYM. For sperm whales, by combining variables, we slightly increased the sperm whale model performance (higher explained deviance and lower root mean squared error). SEAPODYM outputs alone did not seem ideal for modelling prey of deep-diving cetaceans and maybe SEAPODYM was ruled out as a good framework for describing the species distribution, especially for beaked whales. However, SEAPODYM gives new insights about potential prey fields targeted by deep-divers at large spatial scales.”).

Line 75- also see croll et al. Croll, D. A., Marinovic, B., Benson, S., Chavez, F. P., Black, N., Ternullo, R., & Tershy, B. R. (2005). From wind to whales: trophic links in a coastal upwelling system. Marine Ecology Progress Series, 289, 117-130.

ANSWER: Reference included line 75.

Line 98- not clear what the difference between seapodym and seapodym model are?

ANSWER: ‘SEAPODYM model’ refers to the generalised additive model fitted to SEAPODYM variables while ‘SEAPODYM’ refers to the model of Lehodey et al. (2010) that simulated the prey distribution data. We have clarified this on lines 100-102 in the hope that it will be clearer.

Line 166- Why a 29-day sampling window? It seems like this may be appropriate for some variables but for others it would smooth over important information. A sensitivity analysis of choice of sampling window should be considered

ANSWER: We agree with reviewer 2, the 29-day sampling was not appropriate for SEAPODYM variables because the link between species distribution and prey distribution is probably more direct that the one with environmental variables. Consequently, all models were re-fitted with a 7-day temporal resolution for the

Powered by Editorial Manager® and ProduXion Manager® from Aries Systems Corporation

SEAPODYM variables which was the resolution provided by the data provider.

General point of clarification- the spatial aspect of the modeling seems clear, but it would be helpful to have more explicit discussions of how the temporal aspect of the modeling was done, particularly for predator abundances that shift over time. Was there repeat sampling done or is predator abundance from a single survey? The “gapping” described appears to refer mostly to missing spatial data.

ANSWER: We thank the reviewer for pointing out this lack of clarification in the discussion. A new paragraph has been added to discuss this (lines 408-519 “One of the problems faced when studying the distribution of deep-diving cetaceans through habitat modelling is the lack of sighting data, it is necessary to assemble datasets from different surveys to obtain sufficient data to fit habitat models. Sampling is therefore non-uniform, both spatially and temporally, with some areas, years or seasons being much more represented. For example, some years were only sampled in the northwest Atlantic area. Therefore, we chose to combine all the data to ensure we had enough data without considering a temporal effect in the models. However, temporality was taken into account when associating environmental conditions with effort segments, so when there was a sighting, it was associated with environmental conditions averaged over the week for the SEAPODYM variables and over the month for the environmental variables before the sighting, to take into account potential time-lags. When deep-diver data will be large enough to allow a more uniform temporal sampling, the temporal aspect will be taken into account in the models.”).

Reviewer 3 #

The main objective of this paper is to compare habitat models fit to different types of variables to for two deep diving cetaceans: sperm whales and beaked whales. The authors used generalized additive models (GAMs) to compare habitat models fit to 1) environmental variables, 2) simulated prey data from Spatial Ecosystem And POpulation DYnamics Model (SEAPODYM), and 3) combinations of the two variable types. The purpose of this study is to investigate if more direct measures of prey improve the fit of habitat models, when compared to prey proxies such as surface chlorophyll. The authors predict that more proximal variables, like prey biomass (simulated by SEAPODYM) will improve habitat models by reducing the time lag between phytoplankton biomass and the effect on higher trophic levels. The authors have presented and interesting and relevant study, however there are some issues with the methodology that must be addressed prior to publication. Major and minor concerns are detailed below.

Major Concerns

1. My first concern is within the ‘COMBINED’ habitat models for both species. The authors stated that they limited the computational burden within the combined model by only included a reduced number of variables; specifically, by only including variables selected within the final “ENVIRONMENTAL” and “SEAPODYM” models. This strikes me as a red flag and limitation. All relevant variables should be included in the ‘COMBINED’ models. If a variable is not significant in the “ENVIRONMENTAL” model, for example, this does not mean it will automatically not be significant in the “COMBINED” model. Specifically, an interaction between an environmental variable and a SEAPODYM variable could be significant in the “COMBINED” model, and this of course would not show up in the individual “ENVIRONMENTAL” or “SEAPODYM” models.

ANSWER: We understand the point raised by reviewer 3 and understand his concern. We have tried to fit the COMBINED models to all available variables but the computational burden was much too high because the area is very extended and the number of segments is very large (the computer crashed). However, we have revised the method to make it more robust and we hope this will meet the reviewer's expectations. Following Symonds & Moussalli (2011), we have determined the importance of each variable in the ENVIRONMENTAL and SEAPODYM models by summing the Akaike weights of the models in which the variable was selected and ranked all variables. We then included in the selection procedure of the COMBINED

models all variables whose percentages of Akaike weight were greater than 25%, as these were the most important variables in the ENVIRONMENTAL and SEAPODYM models. With this method, all possible relevant variables were included in the COMBINED model and we can see in S3.2 Fig (Supplementary material S3) that a large number of variables included in the selection of the COMBINED model were not important so we think that the method is appropriate to answer our question. The new methodology was included lines 263-271.

2. The authors do not reference/mention spatial auto-correlation or how it was accounted for within the models (e.g. include correlation structure in the model, bin the data at resolutions that are broader than the correlation).

ANSWER: We thank reviewer 3 for pointing this omission. Moran's and Geary's indexes have been calculated to ensure there was no spatial autocorrelation in the data using the 'spdep' R-package. We have added this specification lines 157-158.

3. Explained deviance and AIC are great for model comparison and selection, but are insufficient in terms of model evaluation. AIC and explained deviance are what you use to choose your model, but doesn't tell you anything about model performance. I would like to see an evaluation metric such as area under the curve (AUC) or true skill statistic (TSS). AUC indicates model performance, provide aggregate measures of performance across all possible classification thresholds, and allow performance of models to be compared. TSS measure the discriminatory ability of the SDMs (e.g. Becker et al. 2020).

ANSWER: Following reviewer's recommendations, we have included a new parameter for model evaluation in addition to explained deviance which is commonly used to assess model performance (Becker et al., 2017). We have added the Root Mean Squared Error (RMSE) which measures the prediction errors and the model accuracy (the lower, the better; Neill & Hashemi, 2018). We did not used TSS or AUC as proposed by reviewer 3 because these parameters are used to assess the model performance of presence-only or presence-absence models not density models, which are the models we used. In this case, RMSE was more appropriate so we used it and we hope it meet the reviewer's expectations.

4. A lesser concern is with the observations. As I am not a deep-diving whale expert, I am having trouble reconciling the use of surface observation for a study that focuses on fitting habitat models to sub-surface prey simulations. I am not contending that the use of surface observation is inappropriate, however I do think the methods would benefit from more explanation. For example, do we know how surface observation relates to deep dives for prey? Also including a couple references for studies that have used this type of observation for deep diving species' habitat models previously would be useful.

ANSWER: We understand the reviewer's concern, it may seem inappropriate to use prey simulations at depth to explain the distribution of surface sightings, it would be more appropriate to use tracking data. However, the accepted underlying assumption is that animals observed at the surface are present because they are mostly sensitive to the prey abundance and therefore a high prey biomass at depth could explain the presence of animals at the surface. Brodie et al. (2018) included two subsurface dynamic variables in species distribution models to describe the habitats of four pelagic species in the California Current System and increased the explanatory power and predictive performance of the models for most species. A more systematic use of depth variables could improve the tools available for the planning of human activities, especially for species that would be closely linked to processes at depth. We have added a part in the discussion about this, lines 383-393.

Minor comments

Line 57-59: "cetacean distributions are barely known" is an overgeneralization. If you are referring to deep-diving cetaceans, this needs to be made more explicit. Distributions of some cetaceans are actually quite well known and well modeled (e.g. Abrahms et al. 2019 – blue whales).

Powered by Editorial Manager® and ProduXion Manager® from Aries Systems Corporation

ANSWER: We agree with reviewer 3, we have specified “the deep-diving cetacean distributions” line 59.

Lines 291-294; 307, etc.: The way the authors refer to the “COMBINED” model for the beaked whale is confusing. The authors should state that the “COMBINED” model is identical to the “ENVIRONMENTAL” model at the beginning of the results section referencing beaked whales. Stating the comparison of AIC scores prior to making this point is confusing and makes it seem like these are different models (i.e. contain different variables). Similarly, within the Figure 3, it seems unnecessary to show identical maps for 3a and 3c. Instead, the authors should simply indicate that the “COMBINED” model is identical, and not in fact a separate model with separate results.

ANSWER: We have followed reviewer’s recommendation, we have simplified the text (lines 305-325) and we have removed the plot and the map referring to the beaked whale COMBINED model in figures 3 and 4.

Line 333-334, etc.: Remove line “All selected variables were poorly correlated” lines from the results section. This is inherent based on the methodology. As such, you would only need to report if variables were slightly correlated, e.g.

ANSWER: Done.

Line 418-424: I did find myself wondering the need to combine observation across such a large region. The authors offer an explanation in lines 418-424, but I don’t think this explanation is sufficient. Why not focus this study on one of the regions referenced? Would the authors expect prey data to be more relevant and improve fit at regionally specific scales? Is it possible that such a broad spatial area masks the impacts of prey data?

ANSWER: We thank reviewer 3 for raising this point. One of the problems faced when studying the distribution of deep-diving cetaceans through habitat modelling is the lack of sighting data, it is necessary to assemble datasets from different surveys and different areas to obtain sufficient data to fit habitat models. The combination of sighting data from different ecosystems (e.g. North-East and North-West Atlantic) may mask inter-regional differences in the relationships between cetacean densities and the environmental predictors and may mask the influence of prey data on species distribution. The objective here, was to compare models using environmental variables, prey distribution variables, and a combination of the two types of variables to determine the extent to which the models could be improved and not necessarily explain precisely the mechanisms influencing the distribution of beaked and sperm whales, which would be more consistent at the scale of a smaller region. We built basin-wide models and did not investigate region-specific models in order to leverage large sample sizes for investigating the explanatory power of SEAPODYM variables for habitat use of deep-divers. We acknowledge that pooling regions across an oceanic basin may introduce bias, but this study is a first investigation, and appreciate region-specific models would represent an obvious improvement (once enough data are available at the regional scale). We also think that in a global conservation context, it is important to study the broad scale cetacean distribution to obtain a more global prediction of this distribution and limit anthropogenic threats. We have added a paragraph to discuss this point lines 494-407. We thus agree with reviewer 3 it would be better to focus on one region, which is what we will intend to do in our further studies.

Lines 483-484: Generally, in this part of the discussion, the authors continue to refer to the “COMBINED” models (plural) limiting the uncertainty within the interpolation. However, the authors previously state that the “COMBINED” model is identical to the “ENVIRONMENTAL” model for beaked whales, meaning that these conclusions would not be true for the beaked whales. The authors need to specifically reference the sperm whale example when making these statements/conclusions.

ANSWER: We agree with reviewer 3, we have changed the text through the whole discussion.

References

Powered by Editorial Manager® and ProduXion Manager® from Aries Systems Corporation

	Becker E.A, Forney K.A, Thayre B.J, Debich A.J, Campbell G.S, Whitaker K, et al. (2017). Habitat-based density models for three cetacean species off Southern California illustrate pronounced seasonal differences. Frontiers in Marine Science, 4:121.<br>Brodie S, Jacox M.G, Bograd S.J, Welch H, Dewar H, Scales K.L, et al. (2018). Integrating dynamic subsurface habitat metrics into species distribution models. Frontiers in Marine Science, 5: 219.<br>Cañadas A, de Soto N.A, Aissi M, Arcangeli A, Azzolin M, B-Nagy A., et al. 2018. The challenge of habitat modelling for threatened low density species using heterogeneous data: The case of Cuvier's beaked whales in the Mediterranean. Ecological Indicators, 85: 128–136.<br>Gobeyn S, Mouton A.M, Cord A.F, Kaim A, Volk M, Goethals P.L. (2019). Evolutionary algorithms for species distribution modelling: A review in the context of machine learning. Ecological modelling, 392: 179-195.<br>Lambert C, Mannocci L, Lehodey P, Ridoux V. 2014. Predicting cetacean habitats from their energetic needs and the distribution of their prey in two contrasted tropical regions. PloS one, 9(8): e105958.<br>Lehodey P, Murtugudde R, Senina I. 2010. Bridging the gap from ocean models to population dynamics of large marine predators: a model of mid-trophic functional groups. Progress in Oceanography, 84(1): 69-84.<br>Neill S.P, & Hashemi M.R. (2018). Fundamentals of ocean renewable energy: generating electricity from the sea. Academic Press, pp 193-235.<br>Roberts J.J, Best B.D, Mannocci L, Fujioka E, Halpin P.N, Palka D.L, et al. 2016. Habitat-based cetacean density models for the U.S. Atlantic and Gulf of Mexico. Scientific Report, 6.<br>Symonds M.R, & Moussalli A. (2011). A brief guide to model selection, multimodel inference and model averaging in behavioural ecology using Akaike's information criterion. Behavioral Ecology and Sociobiology, 65(1): 13-21.<br>Virgili A, Authier M, Boisseau O, Cañadas A, Claridge D, Cole T, et al. 2019. Combining multiple visual surveys to model the habitat of deep-diving cetaceans at the basin scale. Global Ecology and Biogeography, 28(3): 300-314.
Additional Information:	
Question	Response
**Financial Disclosure**<br><br>Enter a financial disclosure statement that describes the sources of funding for the work included in this submission. Review the submission guidelines for detailed requirements. View published research articles from *PLOS ONE* for specific examples.<br><br>This statement is required for submission and **will appear in the published article** if the submission is accepted. Please make sure it is accurate.	AV's doctoral research grant was funded by the Direction Générale de l'Armement (DGA).<br>ML was funded by a Ramón y Cajal (RYC-2012-09897) postdoctoral contract of the Spanish Ministry of Economy, Industry and Competitiveness. This study is a contribution to the CHALLENGES (CTM2013- 47032-R) project of the Spanish Ministry of Economy, Industry and Competitiveness.<br>MA and EP are affiliated to ADERA a private business dedicated to providing support for the management of research project in public institutions in Region Nouvelle Aquitaine. In this study, ADERA solely provided support in the form of salaries for authors MA and EP, but did not have any additional role in the study design, data collection and analysis, decision to publish, or preparation of the manuscript. The specific roles of these authors are articulated in the 'author contributions' section.

**Unfunded studies**<br>Enter: *The author(s) received no specific funding for this work.*<br><br>**Funded studies**<br>Enter a statement with the following details:<br>• Initials of the authors who received each award<br>• Grant numbers awarded to each author<br>• The full name of each funder<br>• URL of each funder website<br>• Did the sponsors or funders play any role in the study design, data collection and analysis, decision to publish, or preparation of the manuscript?<br>• **NO** - Include this sentence at the end of your statement: *The funders had no role in study design, data collection and analysis, decision to publish, or preparation of the manuscript.*<br>• **YES** - Specify the role(s) played.<br><br>* typeset	
**Competing Interests**<br><br>Use the instructions below to enter a competing interest statement for this submission. On behalf of all authors, disclose any competing interests that could be perceived to bias this work—acknowledging all financial support and any other relevant financial or non-financial competing interests.<br><br>This statement is required for submission and **will appear in the published article** if the submission is accepted. Please make sure it is accurate and that any funding sources listed in your Funding Information later in the submission form are also declared in your Financial Disclosure statement.<br><br>View published research articles from *PLOS ONE* for specific examples.	MA and EP are employed by the commercial company ADERA which did not play any role in<br>this study beyond that of employer. This does not alter our adherence to PLOS ONE policies<br>on sharing data and materials.

**NO authors have competing interests**<br><br>Enter: *The authors have declared that no competing interests exist.*<br><br>**Authors with competing interests**<br><br>Enter competing interest details beginning with this statement:<br><br>*I have read the journal's policy and the authors of this manuscript have the following competing interests: [insert competing interests here]*<br><br>* typeset	
**Ethics Statement**<br><br>Enter an ethics statement for this submission. This statement is required if the study involved:<br><br>• Human participants<br>• Human specimens or tissue<br>• Vertebrate animals or cephalopods<br>• Vertebrate embryos or tissues<br>• Field research<br><br>Write "N/A" if the submission does not require an ethics statement.<br><br>General guidance is provided below. Consult the submission guidelines for detailed instructions. **Make sure that all information entered here is included in the Methods section of the manuscript.**	N/A

**Format for specific study types**<br><br>**Human Subject Research (involving human participants and/or tissue)**<br>• Give the name of the institutional review board or ethics committee that approved the study<br>• Include the approval number and/or a statement indicating approval of this research<br>• Indicate the form of consent obtained (written/oral) or the reason that consent was not obtained (e.g. the data were analyzed anonymously)<br><br>**Animal Research (involving vertebrate animals, embryos or tissues)**<br>• Provide the name of the Institutional Animal Care and Use Committee (IACUC) or other relevant ethics board that reviewed the study protocol, and indicate whether they approved this research or granted a formal waiver of ethical approval<br>• Include an approval number if one was obtained<br>• If the study involved *non-human primates*, add *additional details* about animal welfare and steps taken to ameliorate suffering<br>• If anesthesia, euthanasia, or any kind of animal sacrifice is part of the study, include briefly which substances and/or methods were applied<br><br>**Field Research**<br><br>Include the following details if this study involves the collection of plant, animal, or other materials from a natural setting:<br>• Field permit number<br>• Name of the institution or relevant body that granted permission	
**Data Availability**<br><br>Authors are required to make all data underlying the findings described fully available, without restriction, and from the time of publication. PLOS allows rare exceptions to address legal and ethical concerns. See the PLOS Data Policy and FAQ for detailed information.	Yes - all data are fully available without restriction

A Data Availability Statement describing where the data can be found is required at submission. Your answers to this question constitute the Data Availability Statement and **will be published in the article**, if accepted.<br><br>**Important:** Stating 'data available on request from the author' is not sufficient. If your data are only available upon request, select 'No' for the first question and explain your exceptional situation in the text box.<br><br>Do the authors confirm that all data underlying the findings described in their manuscript are fully available without restriction?	
**Describe where the data may be found in full sentences. If you are copying our sample text, replace any instances of XXX with the appropriate details.**<br><br>• If the data are **held or will be held in a public repository**, include URLs, accession numbers or DOIs. If this information will only be available after acceptance, indicate this by ticking the box below. For example: *All XXX files are available from the XXX database (accession number(s) XXX, XXX.).*<br>• If the data are all contained **within the manuscript and/or Supporting Information files**, enter the following: *All relevant data are within the manuscript and its Supporting Information files.*<br>• If neither of these applies but you are able to provide **details of access elsewhere**, with or without limitations, please do so. For example:<br><br>*Data cannot be shared publicly because of [XXX]. Data are available from the XXX Institutional Data Access / Ethics Committee (contact via XXX) for researchers who meet the criteria for access to confidential data.*<br><br>*The data underlying the results presented in the study are available from (include the name of the third party*	We confirm that authors had no special access to the underlying data and all interested researchers can obtain the data from http://seamap.env.duke.edu in the same way that the authors obtained data by contacting the data providers via the OBIS SEAMAP website.

and contact information or URL).<br>• This text is appropriate if the data are owned by a third party and authors do not have permission to share the data.<br><br>* typeset	
Additional data availability information:	

Powered by Editorial Manager® and ProduXion Manager® from Aries Systems Corporation

Manuscript

Click here to access/download;Manuscript;Virgili_et_al_PlosOne_Untracked

Towards a better characterisation of deep-diving whales' distributions by using prey distribution model outputs

Auriane Virgili [1]*, Laura Hedon [1], Matthieu Authier [1,2], Beatriz Calmettes [3], Diane Claridge [4], Tim Cole [5], Peter Corkeron [5], Ghislain Dorémus [1], Charlotte Dunn [4], Tim E. Dunn [6], Sophie Laran [1], Patrick Lehodey [3], Mark Lewis [5], Maite Louzao [7], Laura Mannocci [8], José Martínez-Cedeira [9], Pascal Monestiez [10,11], Debra Palka [5], Emeline Pettex [12,2], Jason J. Roberts [13], Leire Ruiz [14], Camilo Saavedra [15], M. Begoña Santos [15], Olivier Van Canneyt [1], José Antonio Vázquez Bonales [16], Vincent Ridoux [1,11]

[1] Observatoire PELAGIS, UMS 3462 CNRS - La Rochelle Université, 5 Allées de l'Océan 17000 La Rochelle, France

[2] ADERA, 162 avenue Albert Schweitzer, CS 60040, 33608 Pessac Cedex, 33000 Pessac, France

[3] Space Oceanography Division, CLS, 8-10 rue Hermes, Ramonville 31520, France

[4] Bahamas Marine Mammal Research Organisation, P.O. Box AB-20714, Marsh Harbour, Abaco, Bahamas

[5] Protected Species Branch, NOAA Fisheries Northeast Fisheries Science, 166 Water St Woods Hole Massachusetts, 02543 USA

[6] Joint Nature Conservation Committee, Inverdee House, Baxter Street, Aberdeen, AB11 9QA, UK

[7] AZTI, Marine Research, Basque Research and Technology Alliance (BRTA), Herrera Kaia, Portualdea z/g, 20110 Pasaia, Spain

[8] MARBEC, Univ Montpellier, CNRS, Ifremer, IRD, Sète, France

[9] CEMMA, Camiño do Ceán, n° 2, 36350, Nigrán, Pontevedra, Spain

[10] BioSP, INRA, 84914 Avignon, France

[11] Centre d'Etudes Biologiques de Chizé - La Rochelle, UMR 7372 CNRS - La Rochelle Université, Villiers-en-Bois, France

[12] Cohabys - ADERA, La Rochelle Université, 2 rue Olympe de Gouges, Bâtiment ILE, 17000 La Rochelle, France

[13] Marine Geospatial Ecology Laboratory, Duke University, Durham, North Carolina, USA

[14] AMBAR Elkartea organisation, Ondarreta Ibilbidea z/g, 48620 Plentzia, Bizkaia, Spain

[15] Instituto Español de Oceanografía, Centro Oceanográfico de Vigo, 36390 Vigo, Spain

[16] Alnilam Research and Conservation, Pradillos 29, 28491-Navacerrada, Madrid, Spain

** Corresponding author*

E-mail: auriane.virgili@univ-lr.fr (AV)

Abstract

In habitat modelling, environmental variables are assumed to be proxies of lower trophic levels distribution and by extension, of marine top predator distributions. More proximal variables, such as potential prey fields, could refine relationships between top predator distributions and their environment. *In situ* data on prey distributions are not available over large spatial scales but, a numerical model, the Spatial Ecosystem And POpulation DYnamics Model (SEAPODYM), provides simulations of the biomass and production of zooplankton and six functional groups of micronekton at the global scale. Here, we aimed at exploring if generalised additive models fitted to simulated prey distribution data better fit deep-diver densities (here beaked and sperm whales) than models fitted to environmental variables and testing whether the combination of environmental and prey distribution data would further improve model fit by comparing their explanatory power. For both taxa, we highlighted a preference for habitats associated with topographic features and thermal fronts but also for habitats where the euphotic zone is extended and with large lower mesopelagic prey. For beaked whales, combining variables did not improve the model performance, no SEAPDOYM variables were selected in the best model, possibly because they prey on organisms less well predicted by SEAPODYM. For sperm whales, by combining variables, we slightly increased the sperm whale model performance (higher explained deviance and lower root mean squared error). SEAPODYM outputs alone did not seem ideal for modelling prey of deep-diving cetaceans and maybe SEAPODYM was ruled out as a good framework for describing the species distribution, especially for beaked whales. However, SEAPODYM gives new insights about potential prey fields targeted by deep-divers at large spatial scales. Considering current SEAPODYM parameterisation, if predators feed on pelagic prey assemblages, the combined use of prey and environmental variables would represent an improvement through more accurate modelling of their densities.

Keywords: Beaked whales, Habitat-based density modelling, SEAPODYM, Sperm whales, prey fields

Introduction

Cetaceans are subject to many anthropogenic threats such as vessel collisions, pollutants, bycatch and underwater noise, leading to the decline of many populations [1-3]. To develop effective conservation strategies, describing and predicting their distribution is essential because cetacean distributions, particularly deep-diving cetacean distributions, are barely known due to missing data which is an important pitfall for conservation. Habitat modelling is a useful tool to predict species distributions [4]. In these models, species observations are related to proximal or distal variables that are assumed to influence their distributions. Proximal variables are biological variables to which the species is assumed to react more directly (*e.g.* prey distribution) than distal variables, which describe the physical environment [5]. Distal variables encompass two types of variables; physiographic variables which are static descriptors that relate to the bathymetry (*e.g.* depth, slope) and oceanographic variables which are dynamic predictors that describe the water masses (*e.g.* sea surface temperature - SST, eddy kinetic energy - EKE, surface chlorophyll *a* concentration). These variables, obtained from satellite imagery or numerical models, are more widely available than proximal data and are often used to describe and predict marine top predator distributions [6-8].

In the marine environment, the surface chlorophyll *a* concentration is commonly used as a proxy for the biomass of phytoplankton in cetacean habitat models [8-10], but the distribution and biomass of low- (phytoplankton and zooplankton) and mid- (micronekton) trophic levels are more proximal predictors of top predator distribution [7,11]. Surface chlorophyll *a* concentration data is widely used because it is derived from satellites and available at a global scale, but there is a time-lag between a change in phytoplankton biomass and its effects on upper trophic levels [7,9,11,12,13,14]. The use of more proximal variables, such as prey biomass, could reduce this lag because marine top predators are expected to be mostly sensitive to prey abundance and because such micronekton outputs are closer to trophic level of apex predator prey [11,15, 16,17,18]. However, the limited spatio-temporal extent of prey data available from *in situ* sampling is a major bottleneck for modelling predator

distributions from the distribution of their prey over large oceanic regions. Ecosystem models simulating the biomass and production of low- and mid-trophic levels provide a new numerical way to fill the prey data gap globally [19-22]. The Spatial Ecosystem And POpulation DYnamics Model (SEAPODYM) provides simulations of the global 3-dimensional distributions of zooplankton and six functional groups of the micronekton defined by their daily vertical migration patterns in three biological layers from 0 to *ca.* 1,000 m. SEAPDOYM was initially developed to model tuna populations for fishery applications [23,24], but its usage was extended to predict turtles, cetaceans and elephant seals habitat use [20,21,22,25,26].

In a previous study [27], we developed habitat models for deep-diving cetaceans (beaked whales *Ziphiidae*, sperm whales *Physeteriidae* and *Kogia* whales *Kogiidae*) using physiographic variables together with surface oceanographic variables. Deep-divers are species of interest because they are sensitive to underwater noise pollution [28,29] and an accurate knowledge of their distribution is crucial to mitigate the impact of human activities. As deep-diving cetaceans spend most of their time at depth and generally feed on meso- to bathypelagic preys (*e.g.* [30,31]), the use of surface variables may limit the ability to correctly infer their habitats. Consequently, using the same sighting survey data as in Virgili et al. [27], here we aimed to explore (1) if models fitted to simulated prey data explained the beaked and sperm whale distributions better than models fitted to environmental variables only and (2) if the combination of environmental and simulated prey data would further improve model fit. We compared the fit and explanatory power of three Generalised Additive Models (GAMs), fitted to beaked and sperm whales sighting data and (1) to environmental variables (hereafter 'ENVIRONMENTAL model'), (2) to prey distribution variables simulated from SEAPODYM (hereafter 'SEAPODYM model', differing from 'SEAPODYM' alone that refers to the model of Lehodey et al. [19] that simulated the prey distribution data) and (3) to a combination of both variable types (hereafter 'COMBINED model'). We then predicted beaked and sperm whale densities in the North Atlantic Ocean and compared the three models. We hypothesised that SEAPODYM variables would better explain the distribution of beaked whales and sperm whales because they are more proximal variables and

because they characterise deeper layers in which deep-divers feed. We also expected a better explanatory power of the model that combined the two types of variables because it considered static variables known to influence the beaked and sperm whale distribution (*e.g.* [32,33]), together with prey distribution data, presumably better suited to describe the species distributions.

Material and methods

Study area

The study area encompassed the North Atlantic Ocean from the Guiana Plateau to Iceland, *i.e.* approximately from 5-65°N, excluding semi-enclosed seas like the Gulf of Mexico, the Hudson Bay, the Baltic sea and the Mediterranean Sea (Fig 1a).

In the North Atlantic Ocean, the general thermohaline circulation is characterised by the formation of deep saline and cold-water masses flowing from the Labrador basin and Greenland Sea southward and warmer surface waters generally flowing northward but affected by a large gyre [34,35]. This subtropical gyre is delimited by the North Atlantic, Azores and Canary Currents in the east and the North Equatorial Current and Gulf Stream in the west. The latter is narrower and swifter than its eastern counterparts and follows the continental slope from Florida to the Grand Banks off Newfoundland. Large seasonal variations of the wind field (except in the subtropical area), high salinity and a general decreasing gradient of temperature from west to east (about 8°C difference) are characteristics of the North Atlantic Ocean central gyre [35]. Within this ocean basin, primary production is quite low in the tropical zone, and varies seasonally elsewhere. Maximum productivity is reported in winter in the subtropical zone; a spring bloom and oligotrophic summer conditions are found at mid-latitude; and productivity is maximum in summer in the subpolar zone [36].

Fig 1. Study area divided into sub-regions showing assembled survey effort (a), along with beaked whale (b) and sperm whale (c) sightings recorded during all surveys. The blue polygon delineates overall study area and other polygons delineate sub-regions. Surveys were carried out along transects following a line-transect methodology (survey details in S1 Table). Sightings were classified by group sizes with each point representing one group of individuals and point size representing the number of animals in a group. Base map from https://www.gebco.net/.

Data collection and collation

In this study, we used some of the effort and sighting data assembled in Virgili et al. ([27]; Fig 1). We only considered beaked and sperm whale sightings and effort data recorded in the North Atlantic Ocean. As deep-divers are difficult to detect during surveys and observations recorded during a single survey are insufficient to fit habitat models, we assembled visual shipboard and aerial surveys performed by nine independent organisations in the North Atlantic Ocean between 1998 and 2015 (details of the surveys in S1 Table). A single common dataset was created, aggregating all survey datasets standardised for units and formats. Effort data were linearized and divided into 5 km segments using ArcGIS 10.3 [37] and the Marine Geospatial Ecology Tools software [38].

Except for the JNCC-ESAS surveys that used a 300-m strip-transect methodology, cetacean sightings were recorded following line-transect methodologies that allow Effective Strip Width (ESW) to be estimated from the measurement of the perpendicular distances to the sightings [39]. For each taxon, we built a hierarchical model to estimate the ESW depending on observation conditions and survey types (for more details, see [27]).

To account for the difficulty to identify them at species level, beaked whale species were pooled into one group including Cuvier's beaked whales (*Ziphius cavirostris),* mesoplodonts (*Mesoplodon* spp.) and northern bottlenose whales *(Hyperoodon ampullatus)*.

A total of 481 sightings of beaked whales and 542 sightings of sperm whales, mainly distributed in the northeast and the northwest Atlantic Ocean (north of the 35°N latitude) were assembled for the present study (Figs 1b and c). Aggregated data represented about 1,101,200 km of on-effort transects (*i.e.* following a transect at a specified speed and altitude with a specified level of visual effort) of which 58% was carried out by plane and the rest by boat (Fig 1a, Table 1).

Moran's and Geary's indexes were calculated to ensure there was no spatial autocorrelation in the data using the 'spdep' R-package [40].

Table 1. Effort performed by platform type and Beaufort sea-state per sector in the North Atlantic Ocean. This table presents the total effort conducted in each sector broken down by platform type and Beaufort sea-state. Beaufort sea-state values reported with decimals in the surveys were rounded up. For the analyses, all segments with Beaufort sea-state > 4 were excluded. 'NE-ATL' means northeast Atlantic Ocean and 'NW-ATL' means northwest Atlantic Ocean.

Sectors	Total survey effort (km and %)	Total aerial effort (km)	Total shipboard effort (km)	Total effort by Beaufort sea-state class (km)				
				0-1	1-2	2-3	3-4	4-7
**NE-ATL**	521,000<br>47 %	70,400	450,600	76,700	118,500	135,700	190,000	200
**NW-ATL**	561,100<br>51 %	549,800	11,300	43,000	122,400	200,900	132,700	62,100
**TROPICS**	19,100<br>2 %	15,400	3,700	10,600	2,500	3,700	2,300	0
**STUDY AREA**	1,101,200	635,600<br>58 %	465,600<br>42 %	130 ,300<br>11 %	243,400<br>22 %	340,300<br>31 %	324,900<br>30 %	62,300<br>6 %

Data processing

Static and oceanographic variables

We considered static and oceanographic variables that can affect the distribution of beaked and sperm whales (Table 2). All variables were resampled at a 0.25° resolution to match the spatial resolution of the SEAPODYM variables (Table 2; S2 Figs.). Depth and slope were derived from GEBCO-08 30 arc-second database (https://www.gebco.net/; 30 arc-second is approximately equal to 0.008°). The surface of canyons and seamounts per 0.25° cell was calculated in ArcGIS 10.3 from the shapefile of canyons and seamounts provided by Harris et al. [41]. Oceanographic variables, relating to the movements of water masses were upscaled at a monthly resolution, *i.e.* averaged over the 29 days prior to each sampled day to avoid gaps in remote sensing oceanographic variables and to consider a potential time-lag between an environmental condition and its effect on higher trophic levels. The mean, standard error and gradient of SST were calculated from the GHRSST Level 4 CMC SST v.2.0 ([42], https://podaac.jpl.nasa.gov/dataset/CMC0.2deg-CMC-L4-GLOB-v2.0). Spatial gradients of SST were calculated as the difference between the minimum and maximum SST values found in the eight pixels surrounding any given pixel of the grid. The Aviso ¼° DT-MADT geostrophic currents dataset was used to compute mean and standard deviation of Sea Surface Height (SSH) and EKE (https://www.aviso.altimetry.fr/en/data/products/sea-surface-height-products/global/madt-h-

uv.html). Net primary production (NPP) was derived from SeaWIFS and Aqua using the Vertically Generalised Production Model (http://orca.science.oregonstate.edu/1080.by.21608day.hdf.vgpm.s.chl.a.sst.php) and was used as a proxy of prey availability.

SEAPODYM variables

SEAPODYM is a numerical model used to compute the spatial distribution of the biomass and the production of organisms, the micronekton and the zooplankton, in different layers of the water column [19,23,43]. For each functional group (zooplankton and micronekton) biomass (in $g.m^{-2}$) and production (in $g.m^{-2}.day^{-1}$) are simulated at a 0.25° resolution. Production is defined as the recruitment of a new cohort of organisms into a micronekton functional group when they reach 1 g body mass (fixed value). Energy transfers from the primary production to the groups of micronekton are parameterised in the model and a system of advection–diffusion–reaction equations, that take into account the vertical migrations of organisms, are used to model recruitment, ageing, mortality, and passive transport with horizontal currents [19,23,43]. In SEAPODYM, the zooplankton is defined as all non-migratory phytoplanktivorous organisms with a size between 200 µm and 2 cm that live in the epipelagic layer [44]. Micronekton encompasses active swimming organisms in the range of 1-20 g and 2-20 cm and includes fishes, crustaceans and cephalopods [44,45]. Depending on the vertical distribution of the organism biomass; three layers are defined according to the euphotic depth (*i.e.* the layer of sea water that receives enough sunlight for photosynthesis to occur; Fig 2; [45]), computed according to the VGPM model (Vertically Generalized Production Model) of Behrenfeld &Falkowski [46]. The epipelagic layer extends from the surface to 1.5*euphotic depth; the upper mesopelagic layer, between 1.5 and 4.5*euphotic depth and the lower mesopelagic layer which extends from 4.5 to 10.5*euphotic depth with a maximum set at 1,000 m [45]. Micronekton can undertake nychthemeral vertical migrations between these three layers. According to migration patterns, micronekton includes six functional groups: epipelagic, non-migrant upper mesopelagic, migrant upper

Table 2. Candidate environmental and SEAPODYM predictors used for the habitat-based density modelling. All variables were resampled at a 0.25° resolution. S: Sources. A: https://www.gebco.net/; 30 arc-second is approximately equal to 0.083°. B: Harris et al. [41]. C: Canada Meteorological Centre [42], https://podaac.jpl.nasa.gov/dataset/CMC0.2deg-CMC-L4-GLOB-v2.0. D: https://www.aviso.altimetry.fr/en/data/products/sea-surface-height-products/global/madt-h-uv.html. E: http://orca.science.oregonstate.edu/1080.by.2160.8day.hdf.vgpm.s.chl.a.sst.php. F: Lehodey et al. [19]. Although it is an oceanographic environmental variable, the euphotic depth was included in the SEAPODYM models because it is this variable that defines the three layers of SEAPODYM.1d: daily; 8d: eight days; 1w: weekly.

Variables used in the study with abbreviations and units	Original Resolution	S	Effects on pelagic ecosystems of potential interest to deep-divers
**Physiographic**			
Depth (m)	30 arc sec	A	Deep-divers feed on squids and fish in the deep-water column
Slope (°)	30 arc sec	A	Associated with currents, high slopes induce prey aggregation or enhanced primary production
Surface of canyons and seamounts in a 0.25° cell – S.can.seam (km²)	30 arc sec	B	Deep-divers are often associated with canyons and seamounts structures; the variable indicates the proportion of this habitat in each cell
**Oceanographic**			
Mean sea surface temperature – SST (°C)	0.2°, 1d	C	Variability over time and horizontal gradients of SST reveal front locations, potentially associated with prey aggregation or enhanced primary production
Mean gradient of SST – SSTgrad (°C)	0.2°, 1d	C	
Mean of sea surface height – SSH (m)	0.25°, 1d	D	High SSH is associated with high mesoscale activity and enhanced prey aggregation or primary production
Mean of eddy kinetic energy – EKE ($m^2.s^{-2}$)	0.25°, 1d	D	High EKE relates to the development of eddies and sediment resuspension inducing prey aggregation
Mean of net primary production – NPP ($mgC.m^{-2}.day^{-1}$)	9 km, 8d	E	Net primary production as a proxy of prey availability
**SEAPODYM variables**			
Euphotic depth – Euph. depth (m)	0.25°, 1w	F	Depth of the euphotic zone as proxy of prey availability
Epipelagic biomass and production – Epi. B. ($g.m^{-2}$) and Epi. P. ($g.m^{-2}.day^{-1}$)	0.25°, 1w	F	
Non-migrant upper mesopelagic biomass and production – U.meso. B. ($g.m^{-2}$) and U.meso. P. ($g.m^{-2}.day^{-1}$)	0.25°, 1w	F	
Migrant upper mesopelagic biomass and production – M.U.meso. B. ($g.m^{-2}$) and M.U.meso. P. ($g.m^{-2}.day^{-1}$)	0.25°, 1w	F	
Non-migrant lower mesopelagic biomass and production – L.meso. B. ($g.m^{-2}$) and L.meso. P. ($g.m^{-2}.day^{-1}$)	0.25°, 1w	F	All these variables relate to the distribution of potential direct or indirect preys of deep-divers.
Migrant lower mesopelagic biomass and production – M.L.meso. B. ($g.m^{-2}$) and M.L.meso. P. ($g.m^{-2}.day^{-1}$)	0.25°, 1w	F	
Highly migrant lower mesopelagic biomass and production – H.m.L.meso. B. ($g.m^{-2}$) and H.m.L.meso. P. ($g.m^{-2}.day^{-1}$)	0.25°, 1w	F	
Zooplankton biomass and production – Pk. B. ($g.m^{-2}$) and Pk. P. ($g.m^{-2}.day^{-1}$)	0.25°, 1w	F	

mesopelagic, non-migrant lower mesopelagic, migrant lower mesopelagic and highly migrant lower mesopelagic organisms (Fig 2; [19,23,45]). These migrations are induced by daylight variations and may be due to a strategy of predator avoidance: during daytime, they dive in deeper waters to avoid predation [47]. Migrant upper mesopelagic and highly migrant lower mesopelagic organisms migrate between the epipelagic layer at night and respectively the upper mesopelagic and lower mesopelagic layers during daytime while the migrant lower mesopelagic organisms migrate between the upper mesopelagic and the lower mesopelagic layers. SEAPODYM makes it possible to obtain, in each cell of a grid, an estimate of the total quantity of organisms of each functional group present in each layer and of the productivity of the functional group in each layer (the greater the production, the richer the environment). A positive relationship of predators with production variables would indicate a relationship with the distribution of smaller and more abundant preys of the functional group while a positive relationship with biomass variables would indicate a relationship with the distribution of larger preys.

Contrary to oceanographic environmental variables, SEAPODYM variables were available at a weekly resolution *i.e.* averaged over the 7 days prior to each sampled day, and not upscaled at a monthly resolution to observe a potentially more direct link between prey and predator distribution. Due to the absence of the lower mesopelagic layer on the continental shelf, a zero, and not an NA (for not available), was assigned to the effort segments on the continental shelf to avoid deleting the segments during model fitting.

Fig 2. Vertical repartition of the functional groups of micronekton in SEAPODYM in the water column. The three depth layers, epi, upper meso and lower mesopelagic layers are defined in SEAPODYM as multiples of EZ (depth of the euphotic zone set maximum at 1,000 m); fishes represent the micronekton functional groups; the red dots represent the zooplankton and the yellow arrows represent the daily vertical migrations of the functional groups between layers.

Habitat-based density modelling

First, we modelled the distribution of beaked whales and sperm whales based on two habitat models: one with physiographic and oceanographic variables ('ENVIRONMENTAL model') and one with

SEAPODYM variables (‘SEAPODYM model’). We fitted GAMs [48,49] with a Tweedie distribution to account for over-dispersion in the cetacean count data [50] with the ‘mgcv’ R-package (R-3.3.1. version; [51]). GAMs extend Generalised Linear Models to allow for smooth, nonlinear functions of predictor variables to be determined by observed data rather than by strict parametric relationships [48,49]. The mean number of individuals per segment was linked to the additive predictors with a log-function with a maximum of four number of knots to attenuate the scope for over-fitting [49]. An offset equal to segment length multiplied by twice the ESW or twice the 300 m-strip for JNCC-ESAS surveys was included ([52]; refer to Virgili et al. [27] for the ESW estimation; ESWs were calculated for each combination of platform - plane or boat, class of Beaufort sea-state and class of observation height). We removed combinations of variables with Pearson correlation coefficients higher than |0.7|and tested all models with combinations of one to four variables to avoid excessive complexity [53]. Environmental variables were poorly correlated with each other and fairly poorly correlated with the SEAPODYM variables (S3.1 Fig.). However, some SEAPODYM variables were correlated with each other, notably the biomass and production variables in the same layer. The Akaike information criterion (AIC, the lower the better; [54] and Akaike weight (‘qpcR’ R-package; [55]) were used for model selection.

Then we built a model, for beaked whales and sperm whales, which combined environmental and SEAPODYM variables (‘COMBINED model’). To limit the computational burden, mostly induced by the extent of the study area, we reduced the number of variables implemented in the variable selection procedure of the COMBINED model. Following Symonds & Moussalli [56], we determined the importance of each variable in the ENVIRONMENTAL and SEAPODYM models by summing the Akaike weights of the models in which the variable was selected and ranked all variables (S3.2 Fig.). We then included in the selection procedure of the COMBINED models all variables whose percentages of Akaike weight were greater than 25%, as these were the most important variables in the ENVIRONMENTAL and SEAPODYM models. As for the ENVIRONMENTAL and SEAPODYM models, all models with combinations of one to four variables were tested and the best COMBINED model with

the lowest AIC and the highest Akaike weight was selected. By design, we expected a better fit of the COMBINED model because we only used the most important variables selected in the first procedure.

Predictions of relative densities were then provided at 0.25° resolution over the entire study region. There were not enough data to fit a model by month or by season (the number of sightings in winter was too low) so we fitted ENVIRONMENTAL, SEAPODYM and COMBINED models to all data of beaked whales and sperm whales but we predicted relative densities for each month of the period and averaged values to obtain climatological prediction maps for the 1998-2015 period. These predictive maps provided the expected distribution of beaked and sperm whales according to static and monthly oceanographic conditions, or prey distributions or a combination of both. To provide uncertainty maps over the 1998-2015 period, averaged variances around the overall predictions were computed as the mean of the standard errors of the monthly predictions; high variances indicate high errors associated with density estimates. Here we only considered uncertainty associated with the densities predicted with the generalised additive model but we ignored other sources of uncertainty such as those associated with the estimation of the SEAPODYM variables.

To model distributions of beaked whales and sperm whales in the North Atlantic Ocean, we gathered survey data from a large region collected over a long period. The cumulative effort was not homogeneously distributed and showed extensive geographical gaps. Consequently, predicting across the entire Atlantic basin would require extensive geographical extrapolation. Therefore, we conducted a gap analysis on environmental space coverage to identify areas where habitat models could produce reliable predictions outside survey blocks, *i.e.* geographical extrapolation, whilst remaining within the ranges of surveyed conditions for the combinations of covariates selected by the models, *i.e*. areas of environmental interpolation ([57-59], as in Virgili et al. [27]. We estimated the convex hull defined by the environmental data used to fit habitat models *i.e.* the smallest convex envelop that contains all the points. We assessed whether a prediction from a set of environmental covariates fell inside or outside this convex hull with the "WhatIf" R package [60-62]. A combination of climatological predictor values that fall inside the convex hull corresponds to an interpolation. We produced maps delineating the

extent of interpolation areas and overlaid them on the density prediction maps to highlight areas with greater reliability and showing that no prediction was made outside interpolation areas.

Finally, the model fit of each model was assessed thanks to the percentage of explained deviance [49,63], the Root Mean Squared Error (RMSE) which measures the prediction errors and the model accuracy (the lower, the better; [64]; 'qpcR' R-package [55]) and a visual inspection of predicted and observed distributions [65]. This allowed a comparison of the three models in order to assess which combination of variables better explained the distribution of beaked and sperm whales.

Results

Beaked whales

The COMBINED model was identical to the ENVIRONMENTAL model as no SEAPODYM variable was selected (Fig 3a; S3.1 Table). The percentage of explained deviance varied between the models (Fig 3; S3.1 Table). The explained deviances of the ENVIRONMENTAL/COMBINED models were identical (39.8%) and higher than the explained deviance of the SEAPODYM model (27.5%). The AIC and the RMSE were the lowest for the ENVIRONMENTAL/COMBINED models (ΔAIC = 335 with the SEAPOYM model and 0.49 vs 0.54 for the RMSEs; Fig 3).

In the best ENVIRONMENTAL/COMBINED model (Fig 3; S3.1 Table), the highest beaked whale relative densities were found at depths *ca.* 2,000 m, spatial gradients of SST *ca.* 2 °C, slope *ca.* 1.5° and SST higher than 20°C in the 2.5-97.5% percentile intervals (Fig 3a). The highest relative densities were predicted on the western side of the North Atlantic Ocean near the Gulf Stream, along continental slopes and along the Mid-Atlantic Ridge (Fig 4a).

In the best SEAPODYM model (Fig 3; S3.1 Table), the highest beaked whale densities were found at biomass of lower mesopelagic organisms *ca.* 1.5 $g.m^{-2}$, low biomass of migrant upper mesopelagic organisms lower than 2 $g.m^{-2}$ per unit surface area, low production of migrant lower mesopelagic organisms (lower than 0.02 $g.m^{-2}.day^{-1}$) and deep euphotic depth (*ca.* 65 m) in the 2.5-97.5% percentile

intervals (Fig 3b). This resulted in a fairly homogenous prediction of beaked whales in oceanic waters beyond the continental slope (Fig 4b).

Interpolation areas varied between the three models with the highest percentage of interpolation observed for the ENVIRONMENTAL/COMBINED models (85%) and the lowest for the SEAPODYM model (74%; Fig 4). In all cases, variances associated with the predictions were small (S4 Fig.).

Fig 3. The functional relationships between beaked (BW) and sperm (SW) whales and the selected variables of the ENVIRONMENTAL (a, c), SEAPODYM (b, d) and COMBINED (a, e) models. Solid lines represent the estimated smooth functions and the blue shaded regions show the approximate 95% confidence intervals. The relative density of individuals (individuals per 750 km²) is shown on the y-axis, where a zero indicates no effect of the covariate. Red vertical lines indicate the 2.5th and 97.5th percentile intervals corresponding to the core data. (D*: explained deviance; AIC: Akaike criterion; RMSE: Root Mean Squared Error; SSTgrad: gradients of Sea Surface Temperature; L.meso. B.: lower mesopelagic biomass; M.L.meso.P.: migrant lower mesopelagic production; M.U.meso.B.: migrant upper mesopelagic biomass; Euph. depth: euphotic depth; SSH: sea surface height; Epi. B.: epipelagic biomass; Epi. P.: epipelagic production).

Sperm whale

The percentage of explained deviance varied between the three models (Fig 3; S3.2 Table). The explained deviance of the ENVIRONMENTAL model (31.1%) was close to that of the COMBINED model (31.9%) and higher than that of the SEAPODYM model (19.3%). The AICs were identical in the ENVIRONMENTAL and COMBINED model and lower than the AIC of the SEAPODYM model (ΔAIC = 188 and 186 with the SEAPOYM model; Fig 3). The RMSE was the lowest for the COMBINED model (0.41) compare to the RMSEs of the ENVIRONMENTAL (0.48) and the SEAPODYM (0.54) models.

In the best ENVIRONMENTAL model (Fig 3; S3.2 Table), the highest sperm whale densities were found at depths *ca.* 2,000 m, SSH higher than |0.1 m|, spatial gradients of SST higher than 2°C and slope *ca.* 1° in the 2.5-97.5% percentile intervals (Fig 3c). The highest densities were predicted on the western side of the North Atlantic Ocean, along continental slopes and along the Mid-Atlantic Ridge (Fig 4c).

In the best SEAPODYM model (Fig 3; S3.2 Table), the highest densities were found at biomass of lower mesopelagic organisms *ca.* 1.5 $g.m^{-2}$, low biomass of epipelagic organisms (lower than 2 $g.m^{-2}$), production of epipelagic organisms higher than 0.2 $g.m^{-2}.day^{-1}$ and deep euphotic depth higher than

60 m in the 2.5-97.5% percentile intervals (Fig 3d). This resulted in fairly homogenous predicted densities in oceanic waters (Fig 4d).

In the best COMBINED model (Fig 3; S3.2 Table), the highest densities were found at depths *ca.* 2,000 m, production of epipelagic organisms higher than 0.2 $g.m^{-2}.day^{-1}$, gradients of SST higher than 2°C and SSH higher than |0.1 m| in the 2.5-97.5% percentile intervals (Fig 3e). This resulted in similar patterns as for the ENVIRONMENTAL model but with more homogenous densities (Fig 4e).

Interpolation areas varied between the three models and were less extended for the SEAPODYM (82%) compared to the ENVIRONMENTAL and COMBINED models (84%; Fig 4). In all cases, variances associated with the predictions were small (S4 Fig.).

Fig 4. The predicted relative densities of beaked (BW) and sperm (SW) whales obtained from the ENVIRONMENTAL (a, c), SEAPODYM (b, d) and COMBINED (a, e) models. Black areas on prediction maps represent zones where we did not extrapolate the predictions. Percentages represent the proportion of the study area defined as interpolation (Inter.) with the gap analysis. Base map from https://www.gebco.net/.

Discussion

Indirect proxies of prey distribution such as SST or SSH are routinely used in habitat models to describe marine top predator distributions because prey data are generally unavailable. The relationships estimated between these proxies and predator occurrence or abundance are therefore indirect. The integration of higher trophic levels in habitat models could improve the model performances and the ecological explanation of the relationships, as shown in Lambert et al. [21] or Putra & Mustika [66]. In this study, we incorporated potentially more direct variables into habitat-based density models by using simulated distributions of functional groups of prey. The models were fitted to sightings of deep-diving cetaceans (beaked and sperm whales) because their habitat use is still poorly known and they face important anthropogenic threats including activities producing high intensity noise (*e.g.,* military sonars or seismic guns [29,67]), which makes them species of major interest. For species that spend most of their time at depth, like deep-divers, information on potential

prey in deep layers could improve current habitat models because they are mostly based on surface variables and not on variables that characterise deep layers [27,32,33,68].

Methodological considerations

It may seem inappropriate to use prey simulations at depth to explain the distribution of surface sightings, it would be more appropriate to use tracking data [22]. However, the accepted underlying assumption (probably stronger than it is in reality) is that animals observed at the surface are present because they are mostly sensitive to the prey abundance [11,15-18] and therefore a high prey biomass at depth could explain the presence of animals at the surface. Very few studies use subsurface or depth variables to explain the distribution of predators but this is increasingly developing. Brodie et al. [69] included two subsurface dynamic variables (bulk buoyancy frequency and isothermal layer depth) in species distribution models to describe the habitats of four pelagic species in the California Current System and increased the explanatory power and predictive performance of the models for most species. A more systematic use of depth variables could improve the tools available for the planning of human activities, especially for species that would be closely linked to processes at depth.

The combination of sighting data from different ecosystems (*e.g.* North-East and North-West Atlantic) may mask inter-regional differences in the relationships between cetacean densities and the environmental predictors, as shown by Mannocci et al. [70] and may mask the influence of prey data on species distribution. The objective here, was to compare models using environmental variables, prey distribution variables, and a combination of the two types of variables to determine the extent to which the models could be improved and not necessarily explain precisely the mechanisms influencing the distribution of beaked and sperm whales, which would be more consistent at the scale of a smaller region. We built basin-wide models and did not investigate region-specific models (*sensu* [70]) in order to leverage large sample sizes for investigating the explanatory power of SEAPODYM variables for habitat use of deep-divers. We acknowledge that pooling regions across an oceanic basin may introduce bias, but this study is a first investigation, and appreciate region-specific models would

represent an obvious improvement (once enough data are available at the regional scale). In a global conservation context, it is also important to study the broad scale cetacean distribution to obtain a more global prediction of this distribution.

One of the problems faced when studying the distribution of deep-diving cetaceans through habitat modelling is the lack of sighting data, it is necessary to assemble datasets from different surveys to obtain sufficient data to fit habitat models [27,32,68]. Sampling is therefore non-uniform, both spatially and temporally, with some areas, years or seasons being much more represented. For example, some years were only sampled in the northwest Atlantic area. Therefore, we chose to combine all the data to ensure we had enough data without considering a temporal effect in the models. However, temporality was taken into account when associating environmental conditions with effort segments, so when there was a sighting, it was associated with environmental conditions averaged over the week for the SEAPODYM variables and over the month for the environmental variables before the sighting, to take into account potential time-lags [11,12,13]. When deep-diver data will be large enough to allow a more uniform temporal sampling, the temporal aspect will be taken into account in the models.

We took care in using appropriate statistical tools for modelling the habitat of species with few sightings [71]. Virgili et al. [71] showed that GAMs with a Tweedie distribution generated reliable habitat modelling predictions for rarely sighted marine predators. We constrained the number of variables in the models to a maximum of 4 (ensuring that they were all significant) to avoid excessive complexity of models and difficulty in their interpretation [53]. Although this could limit the performance of our models, we were confident in the procedure parsimony. Indeed, the explained deviances obtained (between 20 and 40%) were high compared to other studies on cetaceans [63,68], suggestive of a good fit to the data, and for each model, only four or five variables were actually important, the others representing a very small percentage of the Akaike weights (S3.2 Fig.). As a result, four variable models were the best compromise between avoidance of overfitting, reduction of complexity and maximisation of interpretability.

Ability of SEAPODYM and environmental variables to model deep-diver distributions

The model comparison showed that for beaked whales, the explained deviance was higher and the RMSE was lower in the ENVIRONMENTAL model than in the SEAPODYM model, so environmental variables seemed to better explain their distribution than SEAPODYM variables; this could explain why no SEAPODYM variable was selected in the COMBINED model (most important variables for the beaked whale COMBINED model were only environmental variables; S3.2 Fig.). As for Torres et al. [72], it is possible that the integration of prey data did not improve the explanatory power of the models because the prey sampling was not appropriate or there was a mismatch between the distribution of prey and predators because they respond to oceanographic processes at different spatial and temporal scales. The link between predators and the environment may therefore be simpler to model than the link between predators and prey [72] or the spatial resolution of the SEAPODYM variables (0.25°) was quite coarse to depict specific associations between an animal and its environment. In contrast, for sperm whales, the ENVIRONMENTAL and COMBINED models were comparable but the explained deviance and the RMSE were slightly better for the COMBINED model so SEAPODYM variables seemed to provide additional information to that provided by the environmental variables alone. The production of epipelagic organisms appeared to be one of the five most important variables in the sperm whale COMBINED model (S3.2 Fig.). These results agreed with those of Hazen et al. [16] who showed that the performance of a model which included static (depth), oceanographic (salinity and temperature) and biological (deep scattering layer and number of available prey) variables was higher than that of the model which only included prey data. One hypothesis, suggested by Hazen et al. [16], would be that beaked whales do not necessarily track a particular type of prey but rather large-scale physiographic structures while sperm whales would be more pelagic predators, so the prey simulated by SEAPDOYM would correspond more closely to the prey targeted by sperm whales than by beaked

whales. Another hypothesis could be a temporal mismatch between SEAPODYM output and the growth rate of the actual prey of these species as discussed by Chambault et al. [73].

The prey functional groups that were simulated from SEAPODYM may not directly reflect the prey targeted by deep-divers. The three biological layers of SEAPODYM are defined between 0 and *ca.* 1,000 m with organisms ranging from 2 to 20 cm [44,45], but deep-divers can feed beyond 1,000 m on prey bigger than 20 cm, mostly beaked whales [31,74,75]. From stomach contents, Spitz et al. [31] showed that the diet of Cuvier's beaked whales consisted largely of cephalopods bigger than 20 cm, *Teuthowenia megalops* (21,2 ± 3,3 cm) and *Galiteuthis armata* (24,2 ± 2,4 cm) and the diet of sperm whales was mostly composed of cephalopods close to 20 cm, *Histioteuthis bonnellii* (17,8 ± 3,7 cm) in the north-east Atlantic Bay of Biscay. Thus, the functional groups of preys simulated in SEAPODYM may represent only a part of the prey targeted by deep-divers and the probable link could be mediated by deep-diver prey instead. The prey simulated in SEAPODYM may correspond more closely to the prey of the prey targeted by deep-divers. A similar phenomenon was observed in the study of Putra & Mustika [66] which showed that the zooplankton biomass was a strong predictor of the distribution of manta rays (*Mobula birostris*) because they feed directly on zooplankton whereas it was not a strong predictor for dolphins (*Stenella* spp.) because they are second- and third-level consumers. Nevertheless, the study shed light on dolphin foraging habits. It is likely the same situation in our study, the prey targeted by deep-divers may be larger than those simulated in SEAPODYM [31], therefore the simulated trophic levels may be lower than those actually targeted by deep-divers. This may explain why epipelagic organisms were retained in the sperm whale models, many bathypelagic squids and fishes are vertically migrating predators feeding at night in the epipelagic layer. In addition, the functional groups simulated by SEAPODYM encompass multiple organisms (fishes, cephalopods, crustaceans; [44,45]), so deep-diver preys, which are mainly cephalopods, might be poorly represented in the functional groups in terms of biomass and production, even in upper and lower mesopelagic layers, explaining why they were not selected in the COMBINED models.

Although it would be more consistent to fit models at a regional scale, we highlighted interesting relationships with the environment and/or the simulated prey at a large scale. Relationships obtained in the ENVIRONMENTAL models suggested a preference for habitats associated with topographic features and thermal fronts for both species, as previously shown [27,32,33]. Comparatively, SEAPODYM variables reveal areas of prey concentration whereas environmental variables are only indirect proxies of the prey distribution. Putra & Mustika [66] suggested that chlorophyll *a* concentrations are not spatially correlated to high zooplankton biomasses because of the time lag between phytoplankton development and meso-zooplankton growth, therefore dolphin (*Stenella* spp.) densities would be higher where zooplankton biomasses would be higher but where chlorophyll *a* concentrations would be lower. SEAPODYM variables could thus give a better idea of the foraging areas used by the species [19,23,45]. We found that beaked whales were closely related to quite high biomass of lower mesopelagic organisms, low biomass of migrant upper mesopelagic organisms, low production of migrant lower mesopelagic organisms and deep euphotic depth. This would suggest a preference for habitats where the euphotic zone is extended and with large lower mesopelagic prey but not a preference for large upper mesopelagic organisms, which could partly correspond to the prey targeted by beaked whales [31]. Sperm whales were closely related to quite high biomass of lower mesopelagic organisms, low biomass of epipelagic organisms, high production of epipelagic organisms and deep euphotic depth. As for beaked whales, this would suggest a preference for habitats where the euphotic zone is extended, with large lower mesopelagic prey but also abundant small epipelagic prey *i.e.* a productive habitat at the surface which allow the development of large prey at depth. This is partly consistent with the diet of sperm whales which seems more flexible than the diet of beaked whales [31]. The positive relationship with epipelagic production and the negative relationship with epipelagic biomass would indicate habitats with abundant small epipelagic prey and not large epipelagic prey, which would be consistent with the previously mentioned hypothesis that this would be the prey targeted by sperm whale prey. The variables selected in the COMBINED models (or ENVIRONMENTAL model for beaked whale) would suggest that beaked whales were more associated

with physiographic features around 2000 m deep so they might forage on organisms living close to the seabed that may be poorly modelled by SEAPODYM. By contrast, sperm whales would be less constrained by the presence of the slope, and would therefore prey on organisms that are truly pelagic [76], and therefore possibly better modelled by SEAPODYM. The addition of other depth variables such as the bottom temperature could help clarify the relationship with depth [77]. Other variables could also be considered in the models such as SSH fronts, characteristics of the eddies, however the objective here was to compare SEAPDOYM variables to environmental surface variables commonly used and known to influence the deep-diver distribution [27,32, 33, 68].

The euphotic depth, which is the maximum depth of the light zone suitable for phytoplankton photosynthesis, was selected for both beaked whales and sperm whales in the SEAPODYM models with increasing relationships. The euphotic depth was a variable available in SEAPODYM but the mixed layer depth would probably be more informative for deep-divers because it provides information on the depth of the thermocline which is frequently used by diving animals [77], it would therefore be interesting to explore the differences in the relationship between these two types of variables for deep-divers.

For both beaked and sperm whales, depth was selected in the models. There is a great interest in including depth in the models because it is a well-measured variable, *i.e.* with low associated measurement error, unlike remote sensed or modelled variables which may help improving the explanatory power of the models. Pendleton et al. [78] observed similar results for bowhead whales (*Balaena mysticetus*). They compared a model using only environmental and biological variables such as sea ice thickness, sea temperature, diatoms, flagellates, copepods and zooplankton from the Biology Ice Ocean Modeling and Assimilation System (BIOMAS) to a model using these variables plus bathymetry and showed that the best model included bathymetry and BIOMAS variables. The importance of bathymetry suggests there is probably a close relationship between bathymetry and the biophysical processes that determine the distribution of these cetaceans.

Overall, no beaked whales and sperm whales were predicted on the continental shelf because no sightings were recorded, which confirms the non-suitability of this habitat for these species [32,33]. The bathymetry greatly influenced the beaked and sperm whale distributions in the ENVIRONMENTAL and COMBINED models, with the highest densities predicted near slope discontinuities where the bottom is steep and where prey aggregate [79,80]. A west-east density gradient was also observed for both species, which is consistent with the studies of Roberts et al. [32] and Rogan et al. [33]. Sperm whales, appeared to be less linked to these structures than beaked whales and their distribution was slightly more homogeneous. With SEAPODYM variables, predicted densities were lower and prediction maps were smoother (a common scale was applied to facilitate the comparison). Beaked and sperm whales seemed homogeneously distributed in oceanic waters, which was not very consistent with the sightings and highlighted the poorer performance of the models (higher RMSEs). As previously showed in Lambert et al. [21], these smoother predictions may be due to the large resolution of the variables. The spatial resolution of the SEAPODYM variables is currently set to 0.25° but is planned to be refined to 0.125° and a finer spatial resolution may reduce this smooth effect. However, by adding environmental variables, particularly depth, in the sperm whale COMBINED model, the model performance was increased (higher explained deviance and lower RMSE) and the prediction was more spatially detailed with highest densities predicted in the western Atlantic.

We performed a gap analysis to highlight geographical extrapolation areas in which environmental and SEAPODYM variables were out ranges of surveyed conditions. The centre of the study area, near the Mid-Atlantic Ridge, was not surveyed but variable conditions remained within the ranges of surveyed conditions, allowing to predict distribution at a large scale. The gap analysis revealed large gaps in environmental space coverage across the study area, especially in the western part of the Atlantic Ocean for the ENVIRONMENTAL models and in tropical waters for the SEAPODYM models. This suggests that sampling effort was not sufficient in deeper and steeper areas and more intensive sampling effort performed in these areas could help better describe the habitat used by deep-divers. The interpolation areas varied between the different models, from 74/82% for the

SEAPODYM models to 84%/85% for the ENVIRONMENTAL and COMBINED models, which means that 18 and 26% of the area represented an environmental extrapolation with the SEAPODYM models, compared to only 16% with the COMBINED models for sperm whales and 15% in the ENVIRONMENTAL model for beaked whales. The uncertainties were thus greater with the SEAPDYM models and it appeared riskier to perform models at a large scale with SEAPODYM variables only, than with environmental variables. For sperm whales, by combining both types of variables, this risk was slightly reduced compared to the SEAPODYM model and areas of concentration seemed more spatially detailed and better characterised with the COMBINED model, as revealed by a lower RMSE. To meet sperm whale conservation objectives, it seems more valuable to use models combining environmental variables and prey distribution data. Habitat models are regularly used to identify areas where species concentrate in order to set up protection areas [81-83]. They are also useful for predicting species distribution in non-sampled or poorly documented areas [84-85], provided that areas of interpolation and extrapolation are considered [27,57,58]. Considering key habitats of species, particularly foraging habitats, in the design of marine protected areas thanks to the combination of environmental and prey variables (with further development to include prey of all types of predators) would represent an improvement in the tools available for species conservation, and consideration could be given to reducing the overlap between impacting human activities and these key habitats [86,87].

Conclusion

Prey distribution data are not widely available to model the distribution of marine top predators. However, there are models, still not widely used, such as SEAPODYM, which simulate the distribution of functional groups of prey. SEAPODYM outputs alone did not seem ideal for modelling prey of deep-diving cetaceans and maybe SEAPODYM was ruled out as a good framework for describing the distribution of beaked and sperm whales. However, by combining SEAPODYM and environmental variables, we slightly increased the sperm whale model performance and were able to well define the

areas of concentration. In contrast, for beaked whales, combining environmental and SEAPODYM variables did not improved the model performance, no SEAPDOYM variables were selected in the best model, possibly because they prey on organisms more associated to seabed features and consequently less well predicted by SEAPODYM that focus on pelagic prey assemblages. This study was a first investigation and we mostly highlighted the importance of the physiographic variables to understand mechanisms that influence the distribution of deep-diving cetaceans. To improve the results, it would be interesting to refine spatial resolution of the variables, to fit region-specific models or to apply this approach to species whose functional groups of prey would be better represented in SEAPODYM such as baleen whales or small delphinids. The development of SEAPODYM could make SEAPODYM an essential data source to help improving knowledge on species and to be included in management procedures that aimed at improving the conservation of emblematic species such as cetaceans.

Acknowledgments

We are grateful to the many observers who participated in the surveys and collected all the data but also the ships' captains, crews and pilots. We thank Phil Hammond and his team for providing SCANS and CODA survey data. THUNNUS survey was carried out thanks to the collaboration of the General Directorate of Fisheries and Maritime Affairs, Government of Galicia. We thank the *Direction Générale de l'Armement* (DGA), including Odile Gérard and Carole Nahum, for their support during this study. We warmly thank Charlotte Lambert for her help, support and comments. We thank Philippine Chambault and the two other anonymous referees, for their very helpful comments that led to a clearer and much improved manuscript.

Data accessibility

All sighting and effort data used in this study are available in the OBIS SEAMAP database: http://seamap.env.duke.edu/. All data providers can be contacted via the OBIS SEAMAP website.

References

[1] Schipper J, Chanson J.S, Chiozza F, Cox N.A, Hoffmann M, Katariya V, et al. 2008. The Status of the World's Land and Marine Mammals: Diversity, Threat, and Knowledge. Science, 322: 225–230.

[2] Avila I.C, Kaschner K, Dormann C.F. 2018. Current global risks to marine mammals: Taking stock of the threats. Biological Conservation, 221: 44-58.

[3] Panti C, Baini M, Lusher A, Hernandez-Milan G, Rebolledo E.L.B, Unger B, et al. 2019. Marine litter: one of the major threats for marine mammals. Outcomes from the European Cetacean Society workshop. Environmental pollution, 247: 72-79.

[4] Elith J, & Leathwick J.R. 2009. Species distribution models: ecological explanation and prediction across space and time. Annual review of ecology, evolution, and systematics, 40: 677-697.

[5] Guisan A, & Zimmermann N.E. 2000. Predictive habitat distribution models in ecology. Ecological modelling, 135(2-3): 147-186.

[6] Yen P.P, Sydeman W.J, Hyrenbach K.D. 2004. Marine bird and cetacean associations with bathymetric habitats and shallow-water topographies: implications for trophic transfer and conservation. Journal of Marine systems, 50(1-2): 79-99.

[7] Redfern J.V, Ferguson M.C, Becker E.A, Hyrenbach K.D, Good C, Barlow J, et al. 2006. Techniques for cetacean − habitat modeling. Marine Ecology Progress Series, 310: 271–295.

[8] Lambert C, Laran S, David L, Dorémus G, Pettex E, Van Canneyt O, Ridoux V. 2017. How does ocean seasonality drive habitat preferences of highly mobile top predators? Part I: the north-western Mediterranean Sea. Deep Sea Research Part II: Topical Studies in Oceanography, 141: 115-132.

[9] Jaquet N, & Whitehead H. 1996. Scale-dependent correlation of sperm whale distribution with environmental features and productivity in the South Pacific. Marine ecology progress series: 1-9.

[10] Ferguson M.C, Barlow J, Reilly S.B, Gerrodette T. 2006. Predicting Cuvier's (*Ziphius cavirostris*) and Mesoplodon beaked whale population density from habitat characteristics in the eastern tropical Pacific Ocean. Journal of Cetacean Research and Management, 7: 287–299.

[11] Grémillet D, Lewis S, Drapeau L, van Der Lingen C. D, Huggett J. A., Coetzee, J. C, et al. 2008. Spatial match–mismatch in the Benguela upwelling zone: should we expect chlorophyll and sea-surface temperature to predict marine predator distributions? Journal of Applied Ecology, 45(2): 610-621.

[12] Austin D, Bowen W.D, McMillan J.I, Iverson S.J. 2006. Linking Movement, Diving, and Habitat to Foraging Success in a Large Marine Predator. Ecology, 87: 3095–3108.

[13] Cotté C, Guinet C, Taupier-Letage I, Mate B, Petiau E. 2009. Scale-dependent habitat use by a large free-ranging predator, the Mediterranean fin whale. Deep Sea Research Part I: Oceanographic Research Papers, 56: 801–811.

[14] Croll D.A, Marinovic B, Benson S, Chavez F.P, Black N, Ternullo R, et al. (2005). From wind to whales: trophic links in a coastal upwelling system. Marine Ecology Progress Series, 289: 117-130.

[15] Österblom H, Olsson O, Blenckner T, Furness R.W. 2008. Junk-food in marine ecosystems. Oikos, 117: 967–977.

[16] Hazen E.L, Nowacek D.P, Laurent L.S, Halpin P.N, Moretti D.J. 2011. The relationship among oceanography, prey fields, and beaked whale foraging habitat in the Tongue of the Ocean. PloS one, 6(4): e19269.

[17] Louzao M, Valeiras J, García-Barcelona S, González-Quirós R, Nogueira E, Iglesias M, et al. 2019. Marine megafauna niche coexistence and hotspot areas in a temperate ecosystem. Continental Shelf Research, 186: 77-87.

[18] Rockwood R.C, Elliott M.L, Saenz B, Nur, N, Jahncke J. 2020. Modeling predator and prey hotspots: Management implications of baleen whale co-occurrence with krill in Central California. PloS one, 15(7): e0235603.

[19] Lehodey P, Murtugudde R, Senina I. 2010. Bridging the gap from ocean models to population dynamics of large marine predators: a model of mid-trophic functional groups. Progress in Oceanography, 84(1): 69-84.

[20] Abecassis M, Senina I, Lehodey P, Gaspar P, Parker D, Balazs G, et al. 2013. A model of loggerhead sea turtle (*Caretta caretta*) habitat and movement in the oceanic North Pacific. PLoS One, 8(9): e73274.

[21] Lambert C, Mannocci L, Lehodey P, Ridoux V. 2014. Predicting cetacean habitats from their energetic needs and the distribution of their prey in two contrasted tropical regions. PloS one, 9(8): e105958.

[22] Green D.B, Bestley S, Trebilco R, Corney S.P, Lehodey P, McMahon C.R, et al. 2020. Modelled mid-trophic pelagic prey fields improve understanding of marine predator foraging behaviour. Ecography, 43:1-13.

[23] Lehodey P, Senina I, Murtugudde R. 2008. A spatial ecosystem and populations dynamics model (SEAPODYM) – Modeling of tuna and tuna-like populations. Progress in Oceanography, 78(4): 304-318.

[24] Briand K, Molony B, Lehodey P. 2011. A study on the variability of albacore (*Thunnus alalunga*) longline catch rates in the southwest Pacific Ocean. Fisheries Oceanography, 20(6): 517-529.

[25] Pérez-Jorge S, Tobeña M, Prieto R, Vandeperre F, Calmettes B, Lehodey P, et al. 2020. Environmental drivers of large-scale movements of baleen whales in the mid-North Atlantic Ocean. Diversity and Distributions, 26: 683–698.

[26] Chambault P, de Thoisy B, Heerah K, Conchon A, Barrioz S, Dos Reis V, et al. (2016). The influence of oceanographic features on the foraging behavior of the olive ridley sea turtle *Lepidochelys olivacea* along the Guiana coast. Progress in Oceanography, 142: 58-71.

[27] Virgili A, Authier M, Boisseau O, Cañadas A, Claridge D, Cole T, et al. 2019. Combining multiple visual surveys to model the habitat of deep-diving cetaceans at the basin scale. Global Ecology and Biogeography, 28(3): 300-314.

[28] Fernández A, Edwards J.F, Rodríguez F, Espinosa de los Monteros A, Herráez P, Castro, P., el al. (2005). "Gas and fat embolic syndrome" involving a mass stranding of beaked whales (family *Ziphiidae*) exposed to anthropogenic sonar signals. Veterinary Pathology, 42: 446–457.

[29] D'Amico A, Gisiner R.C, Ketten D.R, Hammock J.A, Johnson C, Tyack P.L, et al. (2009). Beaked whale strandings and naval exercises. Aquatic Mammals, 35: 452–472.

[30] Perrin W.F, Würsig B, Thewissen J.G.M. (Eds.). 2009. Encyclopedia of marine mammals. Academic Press.

[31] Spitz J, Cherel Y, Bertin S, Kiszka J, Dewez A, Ridoux V. 2011. Prey preferences among the community of deep-diving odontocetes from the Bay of Biscay, Northeast Atlantic. Deep Sea Research I, 58: 273–282.

[32] Roberts J.J, Best B.D, Mannocci L, Fujioka E, Halpin P.N, Palka D.L, et al. 2016. Habitat-based cetacean density models for the U.S. Atlantic and Gulf of Mexico. Scientific Report, 6.

[33] Rogan E, Cañadas A, Macleod K, Santos M.B, Mikkelsen B, Uriarte A, et al. 2017. Distribution abundance and habitat use of deep diving cetaceans in the North-East Atlantic. Deep Sea Research Part II: Topical Studies in Oceanography, 141: 8–19.

[34] Levin L.A, & Gooday A. 2003. The Deep Atlantic Ocean. Ecosystems of the deep oceans. (Tyler PA, Ed.). pp. 111-178. Amsterdam; New York: Elsevier.

[35] Tomczak M, & Godfrey J.S. 2003. Regional oceanography: an introduction. Elsevier.

[36] Campbell J.W, & Aarup T. 1992. New production in the North Atlantic derived from seasonal patterns of surface chlorophyll. Deep Sea Research Part A. Oceanographic Research Papers, 39: 1669–1694.

[37] ESRI. 2016. ArcGIS – A complete integrated system. Redlands, CA: Environmental Systems Research Institute Inc. Retrieved from http://esri.com/arcgis.

[38] Roberts J.J, Best B.D, Dunn D.C, Treml E.A, Halpin P.N. 2010. Marine geospatial ecology tools: An integrated framework for ecological geoprocessing with ArcGIS, Python, R, MATLAB, and C++. Environmental Modelling and Software, 25: 1197–1207.

[39] Buckland S.T, Rexstad E.A, Marques T.A, Oedekoven C.S. 2015. Distance sampling: methods and applications. Springer.

[40] Bivand R.S. & Wong D.W.S (2018) Comparing implementations of global and local indicators of spatial association TEST, 27(3), 716-748. Retrieved from https://doi.org/10.1007/s11749-018-0599-x.

[41] Harris P.T, Macmillan-Lawler M, Rupp J, Baker E.K. 2014. Geomorphology of the oceans. Marine Geology, 352: 4–24.

[42] Canada Meteorological Center. 2012. GHRSST level 4 CMC0.2deg global foundation sea surface temperature analysis (GDS version 2). Ver. 2.0. PO.DAAC. Pasadena, CA.

[43] Lehodey P, Andre J.M, Bertignac M, Hampton J, Stoens A, Menkès C, et al. 1998. Predicting skipjack tuna forage distributions in the equatorial Pacific using a coupled dynamical bio-geochemical model. Fisheries Oceanography, 7(3-4): 317-325.

[44] Conchon A. 2016. Modeling marine zooplankton and micronekcton (Doctoral dissertation, La Rochelle University).

[45] Lehodey P, Conchon A, Senina I, Domokos R, Calmettes B, Jouanno J, et al. 2014. Optimization of a micronekton model with acoustic data. ICES Journal of Marine Science, 72(5): 1399-1412.

[46] Behrenfeld M.J, & Falkowski P.G. 1997. A consumer's guide to phytoplankton primary productivity models. Limnology and Oceanography, 42: 1479–1491.

[47] Hays G.C. 2003. A review of the adaptive significance and ecosystem consequences of zooplankton diel vertical migrations. In Migrations and Dispersal of Marine Organisms (pp. 163-170). Springer, Dordrecht.

[48] Hastie T & Tibshirani R. 1986. Generalized Additive Models. Statistical Science, 3: 297-313.

[49] Wood S.N. 2006. On confidence intervals for generalized additive models based on penalized regression splines. Australian & New Zealand Journal of Statistics, 48: 445–464.

[50] Foster S.D, & Bravington M.V. 2013. A Poisson-Gamma model for analysis of ecological non-negative continuous data. Environmental and ecological statistics, 20: 533–552.

[51] Wood S. 2013. mgcv: Mixed GAM Computation Vehicle with GCV/AIC/REML smoothness estimation. Retrieved 7 July 2014, from http://cran.r-project.org/web/packages/mgcv/index.html.

[52] Hedley S.L, & Buckland S.T. 2004. Spatial models for line transect sampling. Journal of Agricultural, Biological, and Environmental Statistics, 9(2): 181–199.

[53] Mannocci L, Catalogna M, Dorémus G, Laran S, Lehodey P, Massart W, et al. 2014. Predicting cetacean and seabird habitats across a productivity gradient in the South Pacific gyre. Progress in Oceanography, 120: 383–398.

[54] Burnham K.P, & Anderson D.R. 2002. *Model selection and multimodel inference*, 2nd edn. Springer, New York.

[55] Spiess A. 2014. qpcR: Modelling and analysis of real-time PCR data. R package version 1.4-0. Retrieved from https://CRAN.R-project.org/package=qpcR.

[56] Symonds M.R, & Moussalli A. (2011). A brief guide to model selection, multimodel inference and model averaging in behavioural ecology using Akaike's information criterion. Behavioral Ecology and Sociobiology, 65(1): 13-21.

[57] Jennings M.D. 2000. Gap analysis: Concepts, methods, and recent results. Landscape Ecology, 15: 5–20.

[58] Mannocci L, Roberts J.J, Halpin P.N, Authier M, Boisseau O, Bradai M.N, et al. 2018. Assessing cetacean surveys throughout the Mediterranean Sea: A gap analysis in environmental space. Scientific Reports, 8(1): 3126.

[59] Bouchet P.J, Miller D.L, Roberts J.J, Mannocci L, Harris C.M, Thomas L. 2019. From here and now to there and then: Practical recommendations for extrapolating cetacean density surface models to novel conditions. Technical report 2019-01 v1.0, Centre for Research into Ecological & Environmental Modelling (CREEM), University of St Andrews, 59 p.

[60] Stoll H, King G, Zeng L. 2014. WhatIf: Software for evaluating counterfactuals. R package version 1.5-6. Retrieved from https:// cran.r-project.org/web/packages/WhatIf/index.html.

[61] Authier M, Saraux C, Péron C. 2016. Variable selection and accurate predictions in habitat modelling: A shrinkage approach. Ecography, 40: 549–560.

[62] King G, & Zeng L. 2007. When can history be our guide? The pitfalls of counterfactual inference. International Studies Quarterly, 51: 183–210.

[63] Becker E.A, Forney K.A, Thayre B.J, Debich A.J, Campbell G.S, Whitaker K, et al. (2017). Habitat-based density models for three cetacean species off Southern California illustrate pronounced seasonal differences. Frontiers in Marine Science, 4:121.

[64] Neill S.P, & Hashemi M.R. (2018). *Fundamentals of ocean renewable energy: generating electricity from the sea*. Academic Press, pp 193-235.

[65] Becker E.A, Carretta J.V, Forney K.A, Barlow J, Brodie S, Hoopes R, et al. (2020). Performance evaluation of cetacean species distribution models developed using generalized additive models and boosted regression trees. Ecology and evolution, 10(12): 5759-5784.

[66] Putra M.I.H, & Mustika P.L.K. 2020. Incorporating in situ prey distribution into foraging habitat modelling for marine megafauna in the Solor waters of the Savu Sea, Indonesia. Aquatic Conservation: marine and freshwater ecosystems, 30(12): 2384-2401.

[67] Stone C.J, & Tasker M.L (2006). The effects of seismic airguns on cetaceans in UK waters. Journal of Cetacean Research and Management, 8: 255–263.

[68] Cañadas A, de Soto N.A, Aissi M, Arcangeli A, Azzolin M, B-Nagy A., et al. 2018. The challenge of habitat modelling for threatened low density species using heterogeneous data: The case of Cuvier's beaked whales in the Mediterranean. Ecological Indicators, 85: 128–136.

[69] Brodie S, Jacox M.G, Bograd S.J, Welch H, Dewar H, Scales K.L, et al. (2018). Integrating dynamic subsurface habitat metrics into species distribution models. Frontiers in Marine Science, 5: 219.

[70] Mannocci L, Roberts J.J, Pedersen E.J, Halpin P.N. 2020. Geographical differences in habitat relationships of cetaceans across an ocean basin. Ecography, 43:1-10

[71] Virgili A, Authier M, Monestiez P, Ridoux V. (2018). How many sightings to model rare marine species distributions. PLoS ONE, 13(3): e0193231.

[72] Torres L.G, Read A.J, Halpin P. 2008. Fine-scale habitat modeling of a top marine predator: Do prey data improve predictive capacity. Ecological Applications, 18(7): 1702-1717.

[73] Chambault P, Roquet F, Benhamou S, Baudena A, Pauthenet E, de Thoisy B, et al. (2017). The Gulf Stream frontal system: A key oceanographic feature in the habitat selection of the leatherback turtle? Deep Sea Research Part I: Oceanographic Research Papers, 123: 35-47.

[74] Watwood S.L, Miller P.J, Johnson M, Madsen P.T, Tyack P.L. 2006. Deep-diving foraging behaviour of sperm whales (*Physeter macrocephalus*). Journal of Animal Ecology, 75(3): 814-825.

[75] Madsen P.T, de Soto N.A, Tyack P.L., Johnson M. 2014. Beaked whales. Current Biology, 24(16): 728–730.

[76] Irvine L, Palacios D.M, Urbán J, Mate B. (2017). Sperm whale dive behavior characteristics derived from intermediate-duration archival tag data. Ecology and evolution, 7(19): 7822-7837.

[77] Chambault P, Fossette S, Heide-Jørgensen M.P, Jouannet D, Vély M. (2021). Predicting seasonal movements and distribution of the sperm whale using machine learning algorithms. Ecology and evolution, 11(3): 1432-1445.

[78] Pendleton D.E, Holmes E.E, Redfern J, Zhang J. 2020. Using modelled prey to predict the distribution of a highly mobile marine mammal. Diversity and Distributions, 26(11): 1612-1626.

[79] Bost C.A, Cotté C, Bailleul F, Cherel Y, Charrassin J.B, Guinet C, et al. 2009. The importance of oceanographic fronts to marine birds and mammals of the southern oceans. Journal of Marine Systems, 78: 363–376.

[80] Woodson C.B. & Litvin S.Y. 2015. Ocean fronts drive marine fishery production and biogeochemical cycling. Proceedings of the National Academy of Sciences, 112(6): 1710–1715.

[81] Cañadas A, Sagarminaga R, De Stephanis R, Urquiola E, Hammond P.S. 2005. Habitat preference modelling as a conservation tool: Proposals for marine protected areas for cetaceans in southern Spanish waters. Aquatic Conservation: Marine and Freshwater Ecosystems, 15(5): 495–521.

[82] McShea W.J. 2014. What are the roles of species distribution models in conservation planning? Environmental Conservation, 41: 93–96.

[83] Hazen E.L, Palacios D.M, Forney K.A, Howell E.A, Becker E, Hoover A.L, et al. 2017. WhaleWatch: A dynamic management tool for predicting blue whale density in the California Current. Journal of Applied Ecology, 54(5): 1415–1428.

[84] Fleishman E, Nally R.M, Fay J.P, Murphy D.D. 2001. Modeling and predicting species occurrence using broad-scale environmental variables: An example with butterflies of the Great Basin. Conservation Biology, 15(6): 1674–1685.

[85] Lumaret J.P, & Jay-Robert P. 2002. Modelling the species richness distribution of French dung beetles (*Coleoptera, Scarabaeidae*) and delimiting the predictive capacity of different groups of explanatory variables. Global Ecology and Biogeography, 11(4): 265–277.

[86] Thaxter C.B, Lascelles B, Sugar K, Cook A.S, Roos S, Bolton M, et al. 2012. Seabird foraging ranges as a preliminary tool for identifying candidate Marine Protected Areas. Biological Conservation, 156: 53–61.

[87] Young H.S, Maxwell S.M, Conners M.G, Shaffer S.A. 2015. Pelagic marine protected areas protect foraging habitat for multiple breeding seabirds in the central Pacific. Biological Conservation, 181: 226–235.

Supporting information

S1 Table. Details of surveys used in the analyses. Total effort represents the total length of transects of each survey (without removing the transects with a Beaufort sea-state > 4). NE-ATL: Northeast Atlantic Ocean; NW-ATL: Northwest Atlantic Ocean.

S2 File. Average conditions of the static, oceanographic and SEAPODYM variables over the entire period (from 1998 to 2015). Base map from https://www.gebco.net/.

S3 File. Correlations between environmental and SEAPODYM variables and model outputs for beaked and sperm whales.

S4 Fig. Uncertainty maps representing the standard error associated with the predicted relative density of beaked (BW) and sperm (SW) whales. Black areas represent extrapolation where we did not extrapolate the predictions. Base map from https://www.gebco.net/.

Figure 1

Click here to access/download;Figure;Figure1.tif

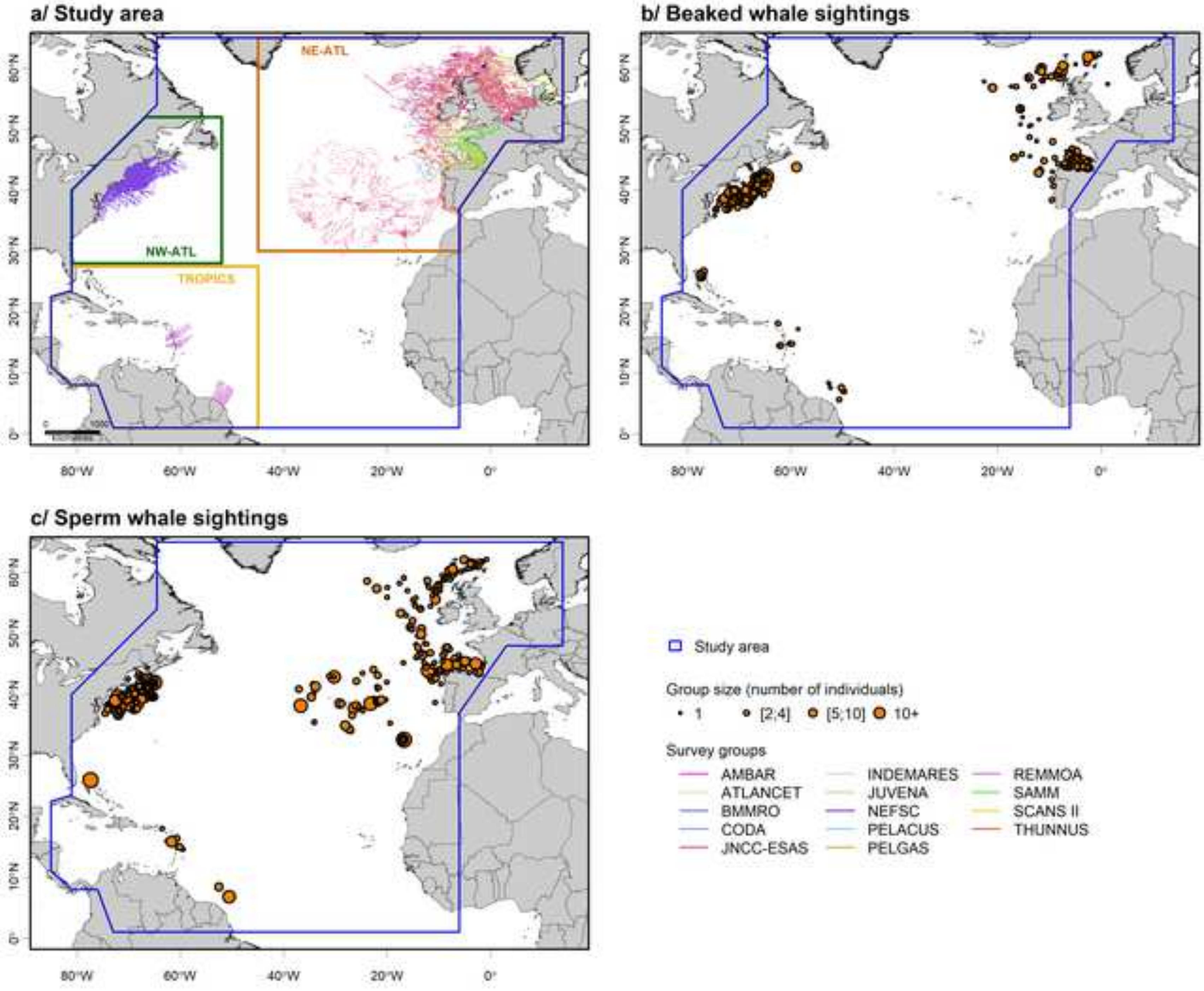

Figure 2

Click here to access/download;Figure;Figure2.tif

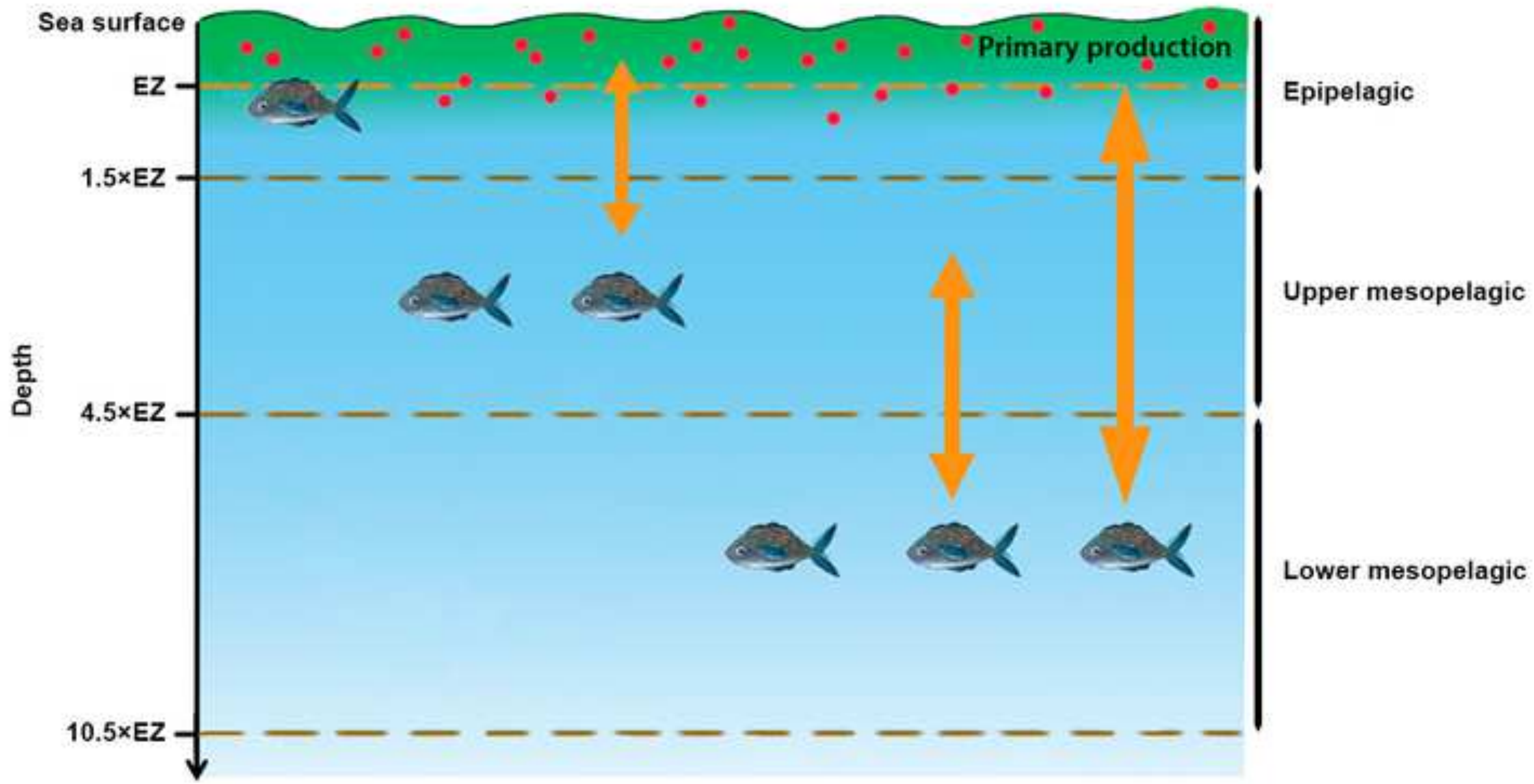

Figure 3

Click here to access/download;Figure;Figure3.tif

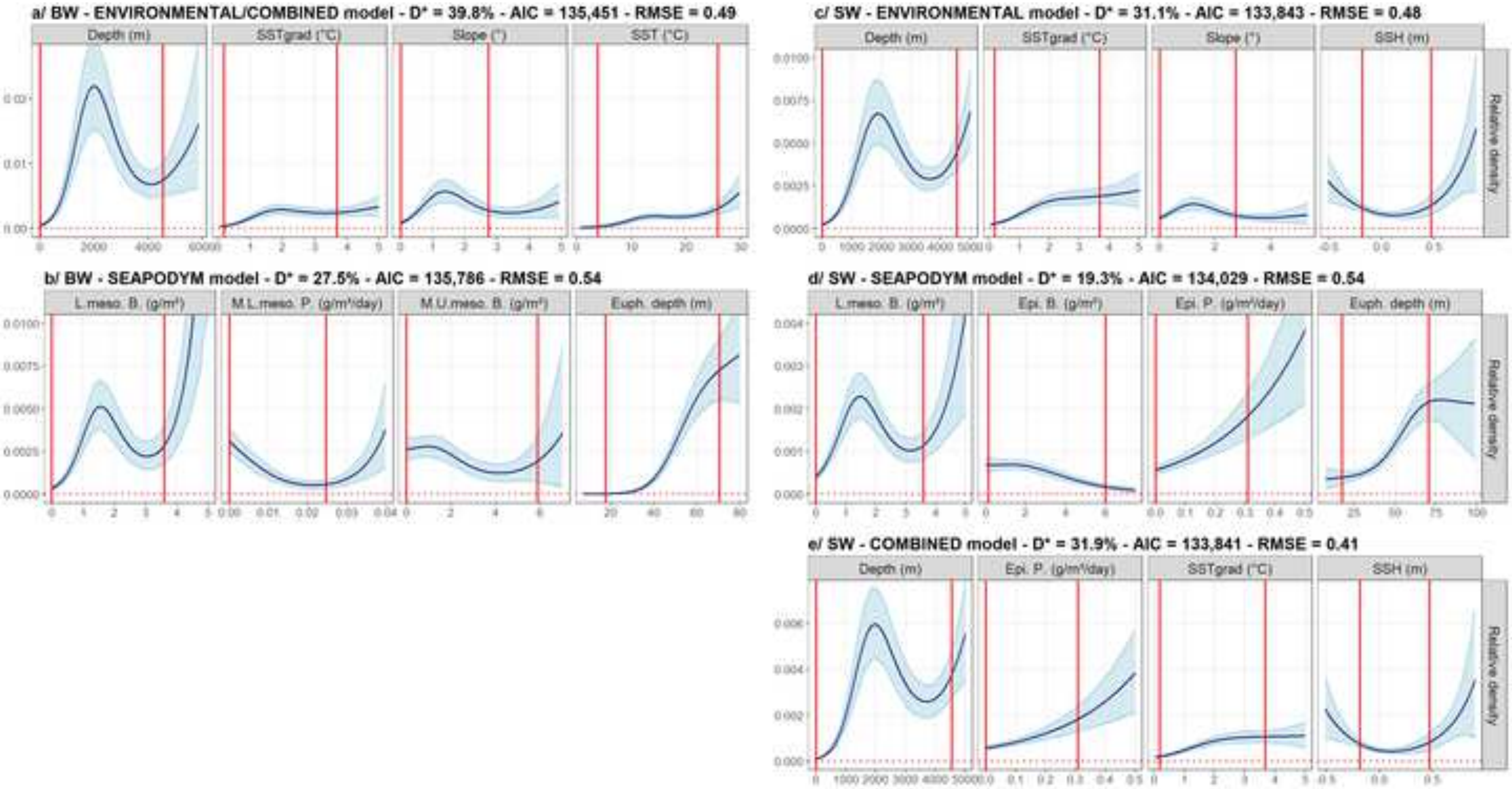

Figure 4

Click here to access/download;Figure;Figure4.tif

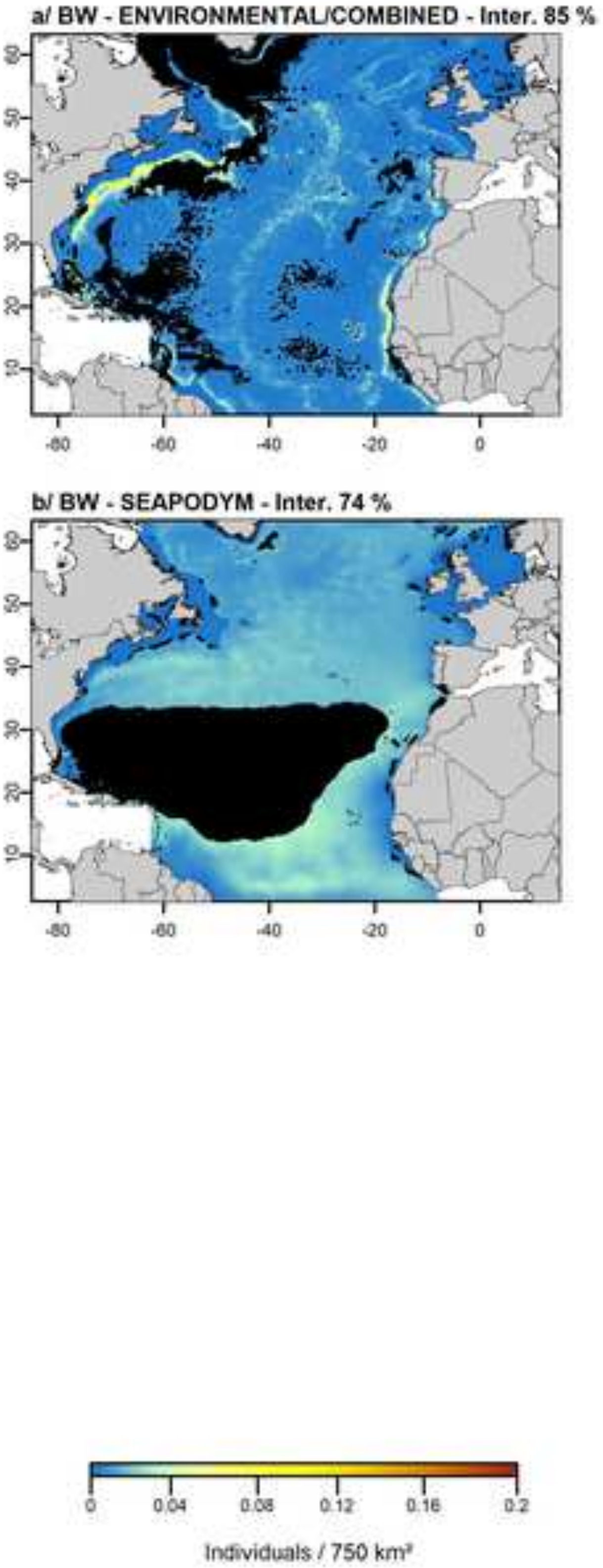

Supporting Information 1

Click here to access/download
Supporting Information
AppendixS1_Details_surveys.pdf

Click here to access/download
Supporting Information
AppendixS2_AveragedConditions.pdf

Click here to access/download
Supporting Information
AppendixS3_Models.pdf

Supporting Information 4

Click here to access/download
Supporting Information
AppendixS4.tif

Towards a better characterisation of deep-diving whales' distributions by using prey distribution model outputs

Auriane Virgili [1]*, Laura Hedon [1], Matthieu Authier [1,2], Beatriz Calmettes [3], Diane Claridge [4], Tim Cole [5], Peter Corkeron [5], Ghislain Dorémus [1], Charlotte Dunn [4], Tim E. Dunn [6], Sophie Laran [1], Patrick Lehodey [3], Mark Lewis [5], Maite Louzao [7], Laura Mannocci [8], José Martínez-Cedeira [9], Pascal Monestiez [10,11], Debra Palka [5], Emeline Pettex [12,2], Jason J. Roberts [13], Leire Ruiz [14], Camilo Saavedra [15], M. Begoña Santos [15], Olivier Van Canneyt [1], José Antonio Vázquez Bonales [16], Vincent Ridoux [1,11]

[1] Observatoire PELAGIS, UMS 3462 CNRS - La Rochelle Université, 5 Allées de l'Océan 17000 La Rochelle, France

[2] ADERA, 162 avenue Albert Schweitzer, CS 60040, 33608 Pessac Cedex, 33000 Pessac, France

[3] Space Oceanography Division, CLS, 8-10 rue Hermes, Ramonville 31520, France

[4] Bahamas Marine Mammal Research Organisation, P.O. Box AB-20714, Marsh Harbour, Abaco, Bahamas

[5] Protected Species Branch, NOAA Fisheries Northeast Fisheries Science, 166 Water St Woods Hole Massachusetts, 02543 USA

[6] Joint Nature Conservation Committee, Inverdee House, Baxter Street, Aberdeen, AB11 9QA, UK

[7] AZTI, Marine Research, Basque Research and Technology Alliance (BRTA), Herrera Kaia, Portualdea z/g, 20110 Pasaia, Spain

[8] MARBEC, Univ Montpellier, CNRS, Ifremer, IRD, Sète, France

[9] CEMMA, Camiño do Ceán, n° 2, 36350, Nigrán, Pontevedra, Spain

[10] BioSP, INRA, 84914 Avignon, France

[11] Centre d'Etudes Biologiques de Chizé - La Rochelle, UMR 7372 CNRS - La Rochelle Université, Villiers-en-Bois, France

[12] Cohabys - ADERA, La Rochelle Université, 2 rue Olympe de Gouges, Bâtiment ILE, 17000 La Rochelle, France

[13] Marine Geospatial Ecology Laboratory, Duke University, Durham, North Carolina, USA

[14] AMBAR Elkartea organisation, Ondarreta Ibilbidea z/g, 48620 Plentzia, Bizkaia, Spain

[15] Instituto Español de Oceanografía, Centro Oceanográfico de Vigo, 36390 Vigo, Spain

[16] Alnilam Research and Conservation, Pradillos 29, 28491-Navacerrada, Madrid, Spain

** Corresponding author*

E-mail: auriane.virgili@univ-lr.fr (AV)

Abstract

In habitat modelling, environmental variables are assumed to be proxies of lower trophic levels distribution and by extension, of marine top predator distributions. More proximal variables, such as potential prey fields, could refine relationships between top predator distributions and their environment. *In situ* data on prey distributions are not available over large spatial scales but, a numerical model, the Spatial Ecosystem And POpulation DYnamics Model (SEAPODYM), provides simulations of the biomass and production of zooplankton and six functional groups of micronekton at the global scale. Here, we aimed at exploring if generalised additive models fitted to simulated prey distribution data better fit deep-diver densities (here beaked and sperm whales) than models fitted to environmental variables and testing whether the combination of environmental and prey distribution data would further improve model fit by comparing their explanatory power. For both taxa, we highlighted a preference for habitats associated with topographic features and thermal fronts but also for habitats where the euphotic zone is extended and with large lower mesopelagic prey. For beaked whales, combining variables did not improve the model performance, no SEAPDOYM variables were selected in the best model, possibly because they prey on organisms less well predicted by SEAPODYM. For sperm whales, by combining variables, we slightly increased the sperm whale model performance (higher explained deviance and lower root mean squared error). SEAPODYM outputs alone did not seem ideal for modelling prey of deep-diving cetaceans and maybe SEAPODYM was ruled out as a good framework for describing the species distribution, especially for beaked whales. However, SEAPODYM gives new insights about potential prey fields targeted by deep-divers at large spatial scales. Considering current SEAPODYM parameterisation, if predators feed on pelagic prey assemblages, the combined use of prey and environmental variables would represent an improvement through more accurate modelling of their densities.

Keywords: Beaked whales, Habitat-based density modelling, SEAPODYM, Sperm whales, prey fields

Introduction

Cetaceans are subject to many anthropogenic threats such as vessel collisions, pollutants, bycatch and underwater noise, leading to the decline of many populations [1-3]. To develop effective conservation strategies, describing and predicting their distribution is essential because cetacean distributions, particularly deep-diving cetacean distributions, are barely known due to missing data which is an important pitfall for conservation. Habitat modelling is a useful tool to predict species distributions [4]. In these models, species observations are related to proximal or distal variables that are assumed to influence their distributions. Proximal variables are biological variables to which the species is assumed to react more directly (*e.g.* prey distribution) than distal variables, which describe the physical environment [5]. Distal variables encompass two types of variables; physiographic variables which are static descriptors that relate to the bathymetry (*e.g.* depth, slope) and oceanographic variables which are dynamic ~~descriptors~~ predictors that describe the water masses (*e.g.* sea surface temperature - SST, eddy kinetic energy - EKE, surface chlorophyll *a* concentration). These variables, obtained from satellite imagery or numerical models, are more widely available than proximal data and are often used to describe and predict marine top predator distributions [6-8].

In the marine environment, the surface chlorophyll *a* concentration is commonly used as a proxy for the biomass of phytoplankton in cetacean habitat models [8-10], but the distribution and biomass of low- (phytoplankton and zooplankton) and mid- (micronekton) trophic levels are more proximal predictors of top predator distribution [7,11]. Surface chlorophyll *a* concentration data is widely used because it is derived from satellites and available at a global scale, but there is a time-lag between a change in phytoplankton biomass and its effects on upper trophic levels [7,9,11,12,13,14]. The use of more proximal variables, such as prey biomass, could reduce this lag because marine top predators are expected to be mostly sensitive to prey abundance and because such micronekton outputs are closer to trophic level of apex predator prey [11,15, 16,17,18]. However, the limited spatio-temporal extent of prey data available from *in situ* sampling is a major bottleneck for modelling predator

distributions from the distribution of their prey over large oceanic regions. Ecosystem models simulating the biomass and production of low- and mid-trophic levels provide a new numerical way to fill the prey data gap globally [19-22]. The Spatial Ecosystem And POpulation DYnamics Model (SEAPODYM) provides simulations of the global 3-dimensional distributions of zooplankton and six functional groups of the micronekton defined by their daily vertical migration patterns in three biological layers from 0 to *ca.* 1,000 m. SEAPDOYM was initially developed to model tuna populations for fishery applications [23,24], but its usage was extended to predict turtles, cetaceans and elephant seals habitat use [20,21,22,25,26].

In a previous study [27], we developed habitat models for deep-diving cetaceans (beaked whales *Ziphiidae*, sperm whales *Physeteriidae* and *kogia* whales *Kogiidae*) using physiographic variables together with surface oceanographic variables. Deep-divers are species of interest because they are sensitive to underwater noise pollution [28,29] and an accurate knowledge of their distribution is crucial to mitigate the impact of human activities. As deep-diving cetaceans spend most of their time at depth and generally feed on ~~mesopelagic~~ meso- to bathypelagic preys (*e.g.* [30,31]), the use of surface variables may limit the ability to correctly infer their habitats. Consequently, using the same sighting survey data as in Virgili et al. [27], here we aimed to explore (1) if models fitted to simulated prey data explained the beaked and sperm whale distributions better than models fitted to environmental variables only and (2) if the combination of environmental and simulated prey data would further improve model fit. We compared the fit and explanatory power of three Generalised Additive Models (GAMs), fitted to beaked and sperm whales sighting data, that used (1) static variables ~~such as depth and slope and oceanographic variables such as SST and EKE~~ (hereafter 'ENVIRONMENTAL model'), (2) simulated prey distribution variables from SEAPODYM (hereafter 'SEAPODYM model', differing from 'SEAPODYM' that refers to the model that simulated prey distribution data) and (3) a combination of both variable types (hereafter 'COMBINED model'). We then predicted beaked and sperm whale densities in the North Atlantic Ocean and compared the three models. We hypothesise that SEAPODYM variables would better explain the distribution of beaked whales and sperm whales

because they are more proximal variables and because they characterise deeper layers in which deep-divers feed. We also expected a better explanatory power of the model that combined the two types of variables because it considered static variables known to influence the beaked and sperm whale distribution (*e.g.* [32,33]), together with prey distribution data, presumably better suited to describe the species distributions.

Material and methods

Study area

The study area encompassed the North Atlantic Ocean from the Guiana Plateau to Iceland, *i.e.* approximately from 5-65°N, excluding semi-enclosed seas like the Gulf of Mexico, the Hudson Bay, the Baltic sea and the Mediterranean Sea (Fig 1a).

In the North Atlantic Ocean, the general thermohaline circulation is characterised by the formation of deep saline and cold-water masses flowing from the Labrador basin and Greenland Sea southward and warmer surface waters generally flowing northward but affected by a large gyre [34,35]. This subtropical gyre is delimited by the North Atlantic, Azores and Canary Currents in the east and the North Equatorial Current and Gulf Stream in the west. The latter is narrower and swifter than its eastern counterparts and follows the continental slope from Florida to the Grand Banks off Newfoundland. Large seasonal variations of the wind field (except in the subtropical area), high salinity and a general decreasing gradient of temperature from west to east (about 8°C difference) are characteristics of the North Atlantic Ocean central gyre [35]. Within this ocean basin, primary production is quite low in the tropical zone, and varies seasonally elsewhere. Maximum productivity is reported in winter in the subtropical zone; a spring bloom and oligotrophic summer conditions are found at mid-latitude; and productivity is maximum in summer in the subpolar zone [36].

Fig 1. Study area divided into sub-regions showing assembled survey effort (a), along with beaked whale (b) and sperm whale (c) sightings recorded during all surveys. The blue polygon delineates overall study area and other polygons delineate sub-regions. Surveys were carried out along transects following a line-transect methodology (survey details in S1 Table). Sightings were classified by group sizes with each point representing

one group of individuals and point size representing the number of animals in a group. Base map from https://www.gebco.net/.

Data collection and collation

In this study, we used some of the effort and sighting data assembled in Virgili et al. ([27]; Fig 1). We only considered beaked and sperm whale sightings and effort data recorded in the North Atlantic Ocean. As deep-divers are difficult to detect during surveys and observations recorded during a single survey are insufficient to fit habitat models, ~~W~~we assembled visual shipboard and aerial surveys performed by nine independent organisations in the North Atlantic Ocean between 1998 and 2015 (details of the surveys in S1 Table). A single common dataset was created, aggregating all survey datasets standardised for units and formats. Effort data were linearized and divided into 5 km segments using ArcGIS 10.3 [37] and the Marine Geospatial Ecology Tools software [38].

Except for the JNCC-ESAS surveys that used a 300-m strip-transect methodology, cetacean sightings were recorded following line-transect methodologies that allow Effective Strip Width (ESW) to be estimated from the measurement of the perpendicular distances to the sightings [39]. For each taxon, we built a hierarchical model to estimate the ESW depending on observation conditions and survey types (for more details, see [27]).

To account for the difficulty to identify them at species level, beaked whale species were pooled into one group including Cuvier's beaked whales (*Ziphius cavirostris),* mesoplodonts (*Mesoplodon* spp.) and northern bottlenose whales *(Hyperoodon ampullatus)*.

A total of 481 sightings of beaked whales and 542 sightings of sperm whales, mainly distributed in the northeast and the northwest Atlantic Ocean (north of the 35°N latitude) were assembled for the present study (Figs 1b and c). Aggregated data represented about 1,101,200 km of on-effort transects (*i.e.* following a transect at a specified speed and altitude with a specified level of visual effort) of which 58% was carried out by plane and the rest by boat (Fig 1a, Table 1).

Moran's and Geary's indexes were calculated to ensure there was no spatial autocorrelation in the data using the 'spdep' R-package [40].

Table 1. Effort performed by platform type and Beaufort sea-state per sector in the North Atlantic Ocean. This table presents the total effort conducted in each sector broken down by platform type and Beaufort sea-state. Beaufort sea-state values reported with decimals in the surveys were rounded up. For the analyses, all segments with Beaufort sea-state > 4 were excluded. 'NE-ATL' means northeast Atlantic Ocean and 'NW-ATL' means northwest Atlantic Ocean.

Sectors	Total survey effort (km and %)	Total aerial effort (km)	Total shipboard effort (km)	Total effort by Beaufort sea-state class (km)				
				0-1	1-2	2-3	3-4	4-7
**NE-ATL**	521,000<br>47 %	70,400	450,600	76,700	118,500	135,700	190,000	200
**NW-ATL**	561,100<br>51 %	549,800	11,300	43,000	122,400	200,900	132,700	62,100
**TROPICS**	19,100<br>2 %	15,400	3,700	10,600	2,500	3,700	2,300	0
**STUDY AREA**	1,101,200	635,600<br>58 %	465,600<br>42 %	130 ,300<br>11 %	243,400<br>22 %	340,300<br>31 %	324,900<br>30 %	62,300<br>6 %

Data processing

Static and oceanographic variables

We considered static and ~~dynamic~~ oceanographic variables that can affect the distribution of beaked and sperm whales (Table 2). All variables were resampled at a 0.25° resolution to match the spatial resolution of the SEAPODYM variables (Table 2; S2 Figs.). Depth and slope were derived from GEBCO-08 30 arc-second database (https://www.gebco.net/; 30 arc-second is approximately equal to 0.008°). The surface of canyons and seamounts per 0.25° cell was calculated in ArcGIS 10.3 from the shapefile of canyons and seamounts provided by Harris et al. [41]. ~~Dynamic~~ Oceanographic variables, ~~which relate~~ relating to the movements of water masses~~,~~ were upscaled at a monthly resolution, *i.e.* averaged over the 29 days prior to each sampled day to avoid gaps in remote sensing oceanographic variables and to consider a potential time-lag between an environmental condition and its effect on higher trophic levels. The mean, standard error and gradient of SST were calculated from the GHRSST Level 4 CMC SST v.2.0 ([42], https://podaac.jpl.nasa.gov/dataset/CMC0.2deg-CMC-L4-GLOB-v2.0). Spatial gradients of SST were calculated as the difference between the minimum and maximum SST values found in the eight pixels surrounding any given pixel of the grid. The Aviso ¼° DT-MADT geostrophic currents dataset was used to compute mean and standard deviation of Sea Surface Height

(SSH) and EKE (https://www.aviso.altimetry.fr/en/data/products/sea-surface-height-products/global/madt-h-uv.html). Net primary production (NPP) was derived from SeaWIFS and Aqua using the Vertically Generalised Production Model (http://orca.science.oregonstate.edu/1080.by.21608day.hdf.vgpm.s.chl.a.sst.php) and was used as a proxy of prey availability.

SEAPODYM variables

SEAPODYM is a numerical model used to compute the spatial distribution of the biomass and the production of organisms, the micronekton and the zooplankton, in different layers of the water column [19,23,43]. For each functional group (zooplankton and micronekton) biomass (in $g.m^{-2}$) and production (in $g.m^{-2}.day^{-1}$) are simulated at a 0.25° resolution. Production is defined as the recruitment of a new cohort of organisms into a micronekton functional group when they reach 1 g body mass (fixed value). Energy transfers from the primary production to the groups of micronekton are parameterised in the model and a system of advection–diffusion–reaction equations, that take into account the vertical migrations of organisms, are used to model recruitment, ageing, mortality, and passive transport with horizontal currents~~driven by sea temperature, currents, net primary production and chlorophyll *a* concentration~~ [19,23,43]. ~~It simulates the development in time and space of micronekton and, more recently, zooplankton [18,22,38,39].~~ In SEAPODYM, the zooplankton is defined as all non-migratory phytoplanktivorous organisms with a size between 200 µm and 2 cm that live in the epipelagic layer [44]. Micronekton encompasses active swimming organisms in the range of 1-20 g and 2-20 cm and includes fishes, crustaceans and cephalopods [44,45]. Depending on the vertical distribution of the organism biomass; three layers are defined according to the euphotic depth (*i.e.* the layer of sea water that receives enough sunlight for photosynthesis to occur; Fig 2; [45]), computed according to the VGPM model (Vertically Generalized Production Model) of Behrenfeld &Falkowski [46]. The epipelagic layer extends from the surface to 1.5*euphotic depth; the upper mesopelagic layer, between 1.5 and 4.5*euphotic depth and the ~~bathypelagic~~ lower mesopelagic layer which

extends from 4.5 to 10.5*euphotic depth with a maximum set at 1,000 m [45]. Micronekton can undertake nychthemeral vertical migrations between these three layers. According to migration patterns, micronekton includes six functional groups: epipelagic, non-migrant upper mesopelagic, migrant upper mesopelagic, non-migrant ~~bathypelagic~~lower mesopelagic, migrant ~~bathypelagic~~ lower mesopelagic and highly migrant ~~bathypelagic~~ lower mesopelagic organisms (Fig 2; [19,23,45]). These migrations are induced by daylight variations and may be due to a strategy of predator avoidance: during daytime, they dive in deeper waters to avoid predation [47]. Migrant upper mesopelagic and highly migrant ~~bathypelagic~~ lower mesopelagic organisms migrate between the epipelagic layer at night and respectively the upper mesopelagic and ~~bathypelagic~~ lower mesopelagic layers during daytime while the migrant ~~bathypelagic~~ lower mesopelagic organisms migrate between the upper mesopelagic and the ~~bathypelagic~~ lower mesopelagic layers. ~~For each functional group (zooplankton and micronekton) biomass (in g.m^{-2}) and production (in g.m^{-2}.day^{-1}) are simulated at a 0.25° resolution. Production is defined as the recruitment of a new cohort of organisms into a micronekton functional group when they reach 1 g body mass (fixed value).~~ SEAPODYM makes it possible to obtain, in each cell ~~of a grid~~, an estimate of the total quantity of organisms of each functional group present in each layer and of the productivity of the functional group in each layer (the greater the production, the richer the environment). A positive relationship of predators with production variables would indicate a relationship with the distribution of smaller and more abundant preys of the functional group while a positive relationship with biomass variables would indicate a relationship with the distribution of larger preys.

Table 2. Candidate environmental and SEAPODYM predictors used for the habitat-based density modelling. All variables were resampled at a 0.25° resolution. A: https://www.gebco.net/; 30 arc-second is approximately equal to 0.0083°. B: Harris et al. [41]. C: Canada Meteorological Centre [42], https://podaac.jpl.nasa.gov/dataset/CMC0.2deg-CMC-L4-GLOB-v2.0. D: https://www.aviso.altimetry.fr/en/data/products/sea-surface-height-products/global/madt-h-uv.html. E: http://orca.science.oregonstate.edu/1080.by.2160.8day.hdf.vgpm.s.chl.a.sst.php. F: Lehodey et al. [19]. Although it is an oceanographic environmental variable, the euphotic depth was included in the SEAPODYM models because it is this variable that defines the three layers of SEAPODYM.1d: daily; 8d: eight days; 1w: weekly.

Variables used in the study with abbreviations and units	Original Resolution	Sources	Effects on pelagic ecosystems of potential interest to deep-divers

**Physiographic**			
Depth (m)	30 arc sec	A	Deep-divers feed on squids and fish in the deep-water column
Slope (°)	30 arc sec	A	Associated with currents, high slopes induce prey aggregation or enhanced primary production
Surface of canyons and seamounts in a 0.25° cell – S.can.seam (km^2)	30 arc sec	B	Deep-divers are often associated with canyons and seamounts structures; the variable indicates the proportion of this habitat in each cell
**Oceanographic**			
Mean sea surface temperature – SST (°C)	0.2°, 1d	C	Variability over time and horizontal gradients of SST reveal front locations, potentially associated with prey aggregation or enhanced primary production
Mean gradient of SST – SSTgrad (°C)	0.2°, 1d	C	
Mean of sea surface height – SSH (m)	0.25°, 1d	D	High SSH is associated with high mesoscale activity and enhanced prey aggregation or primary production
Mean of eddy kinetic energy – EKE ($m^2.s^{-2}$)	0.25°, 1d	D	High EKE relates to the development of eddies and sediment resuspension inducing prey aggregation
Mean of net primary production – NPP ($mgC.m^{-2}.day^{-1}$)	9 km, 8d	E	Net primary production as a proxy of prey availability
**SEAPODYM variables**			
Euphotic depth – Euph. depth (m)	0.25°, 1w	F	Depth of the euphotic zone as proxy of prey availability
Epipelagic biomass and production – Epi. B. ($g.m^{-2}$) and Epi. P. ($g.m^{-2}.day^{-1}$)	0.25°, 1w	F	
Non-migrant ~~Mesopelagic~~ upper mesopelagic biomass and production – U.~~M~~meso. B. ($g.m^{-2}$) and U.m~~M~~eso. P. ($g.m^{-2}.day^{-1}$)	0.25°, 1w	F	
Migrant upper mesopelagic biomass and production – M.U.meso. B. ($g.m^{-2}$) and M.U.meso. P. ($g.m^{-2}.day^{-1}$)	0.25°, 1w	F	
Non-migrant ~~Bathypelagic~~lower mesopelagic biomass and production – L.meso.~~Bathy~~. B. ($g.m^{-2}$) and L.~~Bathy~~meso. P. ($g.m^{-2}.day^{-1}$)	0.25°, 1w	F	All these variables relate to the distribution of potential direct or indirect preys of deep-divers.
Migrant ~~Bathypelagic~~ lower mesopelagic biomass and production – M.L.meso~~bathy~~. B. ($g.m^{-2}$) and M.L.meso~~bathy~~. P. ($g.m^{-2}.day^{-1}$)	0.25°, 1w	F	
Highly migrant ~~bathypelagic~~ lower mesopelagic biomass and production – H.m.L.meso~~bathy~~. B. ($g.m^{-2}$) and H.m.L.meso~~bathy~~. P. ($g.m^{-2}.day^{-1}$)	0.25°, 1w	F	
Zooplankton biomass and production – Pk. B. ($g.m^{-2}$) and Pk. P. ($g.m^{-2}.day^{-1}$)	0.25°, 1w	F	

~~As for static and~~Contrary to oceanographic environmental variables, SEAPODYM variables were ~~upscaled~~ available at a ~~monthly~~ weekly resolution ~~,~~ *i.e.* averaged over the ~~29~~ 7 days prior to each sampled day, and not upscaled at a monthly resolution to observe a potentially more direct link between prey and predator distribution. Due to the absence of the ~~bathypelagic~~ lower mesopelagic layer on the continental shelf, a zero, and not an NA (for not available), was assigned to the effort segments on the continental shelf to avoid deleting the segments during model fitting.

Fig 2. Vertical repartition of the functional groups of micronekton in SEAPODYM in the water column. The three depth layers, epi, upper meso and ~~bathypelagic~~ lower mesopelagic layers ~~,~~ are defined in SEAPODYM as multiples of~~are the layers defined in SEAPODYM;~~ EZ (depth of the euphotic zone set maximum at 1,000 m); fishes represent the micronekton functional groups; the red dots represent the zooplankton and the yellow arrows represent the daily vertical migrations of the functional groups between layers.

Habitat-based density modelling

First, we modelled the distribution of beaked whales and sperm whales based on two habitat models: one with physiographic and oceanographic variables ('ENVIRONMENTAL model') and one with SEAPODYM variables ('SEAPODYM model'). We fitted GAMs [48,49] with a Tweedie distribution to account for over-dispersion in the cetacean count data [50] with the 'mgcv' R-package (R-3.3.1. version; [51]). GAMs extend Generalised Linear Models to allow for smooth, nonlinear functions of predictor variables to be determined by observed data rather than by strict parametric relationships [48,49]. The mean number of individuals per segment was linked to the additive predictors with a log-function with a maximum of four number of knots to attenuate the scope for over-fitting [49]. An offset equal to segment length multiplied by twice the ESW or twice the 300 m-strip for JNCC-ESAS surveys was included ([52]; refer to Virgili et al. [27] for the ESW estimation; ESWs were calculated for each combination of platform - plane or boat, class of Beaufort sea-state and class of observation height). We removed combinations of variables with Pearson correlation coefficients higher than |0.7| and tested all models with combinations of one to four variables to avoid excessive complexity [53]. Environmental variables were poorly correlated with each other and fairly poorly correlated with the SEAPODYM variables (S3.1 Fig.). However, some SEAPODYM variables were correlated with each other,

notably the biomass and production variables in the same layer. ~~Model selection was done with t~~The Akaike information criterion (AIC, the lower the better; [54] and Akaike weight (‘qpcR’ R-package; [55]) were used for model selection.

Then we built a model, for beaked whales and sperm whales, which combined environmental and SEAPODYM variables (‘COMBINED model’). To limit the computational burden, mostly induced by the extent of the study area, we reduced the number of variables implemented in the variable selection procedure of the COMBINED model. Following Symonds & Moussalli [56], we determined the importance of each variable in the ENVIRONMENTAL and SEAPODYM models by summing the Akaike weights of the models in which the variable was selected and ranked all variables (S3.2 Fig.). We then included in the selection procedure of the COMBINED models all variables whose percentages of Akaike weight were greater than 25%, as these were the most important variables in the ENVIRONMENTAL and SEAPODYM models.~~Instead of choosing among the 23 available variables (8 environmental variables and 15 SEAPODYM variables), we selected only the variables that were selected in the five best ENVIRONMENTAL and SEAPODYM models (ordered by AIC) *i.e.* 7 environmental variables and 7 SEAPODYM variables.~~ As for the ENVIRONMENTAL and SEAPODYM models, all models with combinations of one to four variables ~~(among the 14 available variables)~~ were tested and the best COMBINED model with the lowest AIC and the highest Akaike weight was selected. By design, we expected a better fit of the COMBINED model because we only used the ~~best~~ most important variables selected in the first procedure.

Predictions of relative densities were then provided at 0.25° resolution over the entire study region. There were not enough data to fit a model by month or by season (the number of sightings in winter was too low) so we fitted ENVIRONMENTAL, SEAPODYM and COMBINED models to all data of beaked whales and sperm whales but we predicted relative densities for each month of the period and averaged values to obtain climatological prediction maps for the 1998-2015 period. These predictive maps provided the expected distribution of beaked and sperm whales according to static and monthly oceanographic conditions, or prey distributions or a combination of both. To provide uncertainty maps

over the 1998-2015 period, averaged variances around the overall predictions were computed as the mean of the standard errors of the monthly predictions; high variances indicate high errors associated with density estimates. Here we only considered uncertainty associated with the densities predicted with the generalised additive model but we ignored other sources of uncertainty such as those associated with the estimation of the SEAPODYM variables.

To model distributions of beaked whales and sperm whales in the North Atlantic Ocean, we gathered survey data from a large region collected over a long period. The cumulative effort was not homogeneously distributed and showed extensive geographical gaps. Consequently, predicting across the entire Atlantic basin would require extensive geographical extrapolation. Therefore, we conducted a gap analysis on environmental space coverage to identify areas where habitat models could produce reliable predictions outside survey blocks, *i.e.* geographical extrapolation, whilst remaining within the ranges of surveyed conditions for the combinations of covariates selected by the models, *i.e*. areas of environmental interpolation ([57-59], as in Virgili et al. [27]. We estimated the convex hull defined by the environmental data used to fit habitat models *i.e.* the smallest convex envelop that contains all the points. We assessed whether a prediction from a set of environmental covariates fell inside or outside this convex hull with the “WhatIf” R package [60-62]. A combination of climatological predictor values that fall inside the convex hull corresponds to an interpolation. We produced maps delineating the extent of interpolation areas and overlaid them on the density prediction maps to highlight areas with greater reliability and showing that no prediction was made outside interpolation areas.

Finally, the model fit of each model was assessed thanks to the percentage of explained deviance [49,63], and the Root Mean Squared Error (RMSE) which measures the prediction errors and the model accuracy (the lower, the better; [64]; ‘qpcR’ R-package [55]) and a visual inspection of predicted and observed distributions [65]AIC. This allowed a comparison of the three models in order to assess which combination of variables better explained the distribution of beaked and sperm whales.

Results

Beaked whales

The COMBINED model was identical to the ENVIRONMENTAL model as no SEAPODYM variable was selected (Fi[illegible]; S3.1 Table). The percentage of explained deviance varied between the ~~three~~ models (Fig 3; S3.1 Table). The explained deviances of the ENVIRONMENTAL~~ and ~~/COMBINED models were identical (39.8%) and higher than the explained deviance of the SEAPODYM model (27.5%). The AIC~~s~~ and the RMSE were the lowest for the ENVIRONMENTAL/~~ and ~~COMBINED models (ΔAIC = 335 ~~with~~ the SEAPOYM model and 0.49 vs 0.54 for the RMSEs; Fig 3).

In the best ENVIRONMENTAL/COMBINED model (Fig 3; S3.1 Table), the highest beaked whale relative densities were found at depths *ca.* 2,000 m, spatial gradients of SST *ca.* 2 °C, slope *ca.* 1.5° and ~~surface of canyons and seamounts~~SST ~~*ca.* 200 km²~~higher than 2[illegible] in the~~ 5–95~~2.5-[illegible]% percentile intervals (Fig 3a). ~~All selected variables were poorly correlated (S3Fig.).~~ The highest relative densities were predicted on the western side of the North Atlantic Ocean near the Gulf Stream, along continental slopes and along the Mid-Atlantic Ridge (Fig 4a).

In the best SEAPODYM model (Fig 3; S3.1 Table), ~~all selected variables were poorly correlated (S3 Fig.),~~ the highest beaked whale densities were found at ~~low~~ biomass~~es~~ of lower meso~~bathy~~pelagic organisms ~~(~~*ca.* ~~3~~ 1.5 g.m^{-2}~~)~~, low biomass of~~,~~ ~~epipelagic~~ migrant upper mesopelagic organisms ~~(ca~~lower than~~.~~ 2 g.m^{-2}~~)~~ per unit surface area, ~~and~~ low production of migrant lower mesopelagic organisms ~~mesopelagic~~ (~~*ca.*~~ lower than 0.~~01~~ 02 g.m^{-2}.day^{-1}) ~~organisms per unit surface area~~ and deep euphotic depth (*ca.* 65 m) in the ~~5–95~~2.5-97.5% ~~percentile intervals~~ (Fig 3b). This resulted in a fairly homogenous prediction of beaked whales in oceanic waters beyond the continental slope (Fig 4b).

~~The COMBINED model pooled variables of the ENVIRONMENTAL and the SEAPODYM models and was identical to the environmental model as no SEAPODYM variable was selected (Fig 3c; S3.1 Table). This resulted in the same distribution patterns as for the ENVIRONMENTAL model (Fig 4c).~~

Interpolation areas varied between the three models with the highest percentage of interpolation observed for the ENVIRONMENTAL/~~ and ~~COMBINED models (85%) and the lowest for the SEAPODYM model (74%; Fig 4). In all cases, variances associated with the predictions were small (S4 Fig.).

Fig 3. The functional relationships between beaked (BW) and sperm (SW) whales and the selected variables of the ENVIRONMENTAL (a, ~~d~~c), SEAPODYM (b, ~~e~~d) and COMBINED (~~c~~a, ~~f~~e) models. Solid lines represent the estimated smooth functions and the blue shaded regions show the approximate 95% confidence intervals. The relative density of individuals (individuals per 750 km^2) is shown on the y-axis, where a zero indicates no effect of the covariate. Red vertical lines indicate the 2.5^{th} and 97.5^{th} percentile intervals corresponding to the core data. (D*: explained deviance; AIC: Akaike criterion; RMSE: Root Mean Squared Error; SSTgrad: gradients of ~~sea~~ Sea ~~surface~~ Surface ~~temperature~~Temperature; ~~S.can.seam: surface of the cell occupied by canyons and seamounts; Euph. depth: euphotic depth; Bathy~~L.meso. B.: ~~bathypelagic~~ lower mesopelagic biomass; M.L.meso.P: migrant lower mesopelagic production; M.U.meso.B.: migrant upper mesopelagic biomass; Euph. depth: euphotic depth; SSH: sea surface height; Epi. B.: epipelagic biomass; Epi. P.: epipelagic production). ~~Meso. P.: mesopelagic production; EKE: eddy kinetic energy; Epi. P.: epipelagic production).~~

Sperm whale

The percentage of explained deviance varied between the three models (Fig 3; S3.2 Table). The explained deviance of the ENVIRONMENTAL model (31.1%) was ~~similar~~ close to that of the COMBINED model (31.9%) and higher than that of the SEAPODYM model (19.3%). The AICs were identical in the ENVIRONMENTAL and COMBINED model and lower than the AIC of the SEAPODYM model (ΔAIC = 188 and 186 with the SEAPOYM model; Fig 3). The RMSE was the lowest for the COMBINED model (0.41) compare to the RMSEs of the ENVIRONMENTAL (0.48) and the SEAPODYM (0.54) models.

In the best ENVIRONMENTAL model (Fig 3; S3.2 Table), the highest sperm whale densities were found at depths *ca.* 2,000 m, ~~high EKE (log(EKE) > -5 $m^2.s^{-2}$~~SSH higher than |0.1 m|~~)~~, spatial gradients of SST higher than 2°C and slope *ca.* 1° in the ~~5–95~~2.5-97.5% ~~percentile intervals~~ (Fig ~~3d~~3c). ~~All selected variables were poorly correlated (S3 Fig.).~~ The highest densities were predicted on the western side of the North Atlantic Ocean~~ near the Gulf Stream~~, along continental slopes and along the Mid-Atlantic Ridge (Fig ~~4d~~4c).

In the best SEAPODYM model (Fig 3; S3.2 Table), ~~all selected variables were poorly correlated (S3 Fig.),~~ the highest densities were found at ~~low~~ biomass~~ses~~ of lower meso~~bathy~~pelagic organisms ~~(~~(*ca.* ~~3~~ 1.5 $g.m^{-2}$~~)~~, low biomass of ~~and~~ epipelagic organisms (~~*ca.* 0.01~~lower than 2 $g.m^{-2}$)~~ organisms~~, production

of epipelagic organisms higher than 0.2 g.m^{-2}.day^{-1} and deep euphotic depth higher than 60 m in the ~~5–95~~2.5-97.5~~% percentile intervals~~ (Fig ~~3e~~3d). This resulted in fairly homogenous predicted densities in oceanic waters ~~with the highest densities predicted in the tropical waters and near the north-western African coasts~~ (Fig ~~4e~~4d).

In the best COMBINED model (Fig 3; S3.2 Table), ~~all selected variables were poorly correlated (S3 Fig.),~~ the highest densities were found at depths *ca.* 2,000 m, ~~low biomass (*ca.* 0.01 g.m^{-2}) of epipelagic organisms,~~ production of epipelagic organisms higher than 0.2 g.m^{-2}.day^{-1}, ~~and~~ gradients of SST ~~ca.~~ higher than 2°C and SSH higher than |0.1 m| in the ~~5–95~~2.5-97.5% percentile intervals (Fig ~~3f~~3e). This resulted in similar patterns as for the ENVIRONMENTAL model but with more homogenous densities ~~Fig~~ ~~4f~~4e).

Interpolation areas varied between the three models and were less extended for the SEAPODYM (82%) compared to the ENVIRONMENTAL and COMBINED models (84%; Fig 4). In all cases, variances associated with the predictions were small (S4 Fig.).

Fig 4. The predicted relative densities of beaked (BW) and sperm (SW) whales obtained from the ENVIRONMENTAL (a, ~~d~~c), SEAPODYM (b, ~~e~~d) and COMBINED (~~c~~a, ~~f~~e) models. Black areas on prediction maps represent zones where we did not extrapolate the predictions. Percentages represent the proportion of the study area defined as interpolation (Inter.) with the gap analysis. Base map from https://www.gebco.net/.

Discussion

Indirect proxies of prey distribution such as SST or SSH are routinely used in habitat models to describe marine top predator distributions because prey data are generally unavailable. The relationships estimated between these proxies and predator occurrence or abundance are therefore indirect. The integration of higher trophic levels in habitat models could improve the model performances and the ecological explanation of the relationships, as shown in Lambert et al. [21] or Putra & Mustika [66]. In this study, we incorporated potentially more direct variables into habitat-based density models by using simulated distributions of functional groups of prey. ~~The objective was first to compare models using environmental variables to models using simulated prey distribution~~

data as variables in order to evaluate the explanatory power of both types of models. Second, to combine the two types of variables in a single model to determine whether their combination would improve model fit to the data. The models were fitted to sightings of deep-diving cetaceans (beaked and sperm whales) because their habitat use is still poorly known and they face important anthropogenic threats including activities producing high intensity noise (*e.g.,* military sonars or seismic guns [29,67]), which makes them species of major interest. For species that spend most of their time at depth, like deep-divers, information on potential prey in deep layers could improve current habitat models because they are mostly based on surface variables and not on variables that characterise deep layers [27,32,33,68].

Methodological considerations

It may seem inappropriate to use prey simulations at depth to explain the distribution of surface sightings, it would be more appropriate to use tracking data [22]. However, the accepted underlying assumption (probably stronger than it is in reality) is that animals observed at the surface are present because they are mostly sensitive to the prey abundance [11,15-18] and therefore a high prey biomass at depth could explain the presence of animals at the surface. Very few studies use subsurface or depth variables to explain the distribution of predators but this is increasingly developing. Brodie et al. [69] included two subsurface dynamic variables (bulk buoyancy frequency and isothermal layer depth) in species distribution models to describe the habitats of four pelagic species in the California Current System and increased the explanatory power and predictive performance of the models for most species. A more systematic use of depth variables could improve the tools available for the planning of human activities, especially for species that would be closely linked to processes at depth.

The combination of sighting data from different ecosystems (*e.g.* North-East and North-West Atlantic) may also mask inter-regional differences in the relationships between cetacean densities and the environmental predictors, as shown by Mannocci et al. [70] and may mask the influence of prey data on species distribution. The objective here, was to compare models using environmental

variables, prey distribution variables, and a combination of the two types of variables to determine the extent to which the models could be improved and not necessarily explain precisely the mechanisms influencing the distribution of beaked and sperm whales, which would be more consistent at the scale of a smaller region. We built basin-wide models and did not investigate region-specific models (*sensu* [70]) in order to leverage large sample sizes for investigating the explanatory power of SEAPODYM variables for habitat use of deep-divers. We acknowledge that pooling regions across an oceanic basin may introduce bias, but this study is a first investigation, and appreciate region-specific models would represent an obvious improvement (once enough data are available at the regional scale). In a global conservation context, it is also important to study the broad scale cetacean distribution to obtain a more global prediction of this distribution.

One of the problems faced when studying the distribution of deep-diving cetaceans through habitat modelling is the lack of sighting data, it is necessary to assemble datasets from different surveys to obtain sufficient data to fit habitat models [27,32,68]. Sampling is therefore non-uniform, both spatially and temporally, with some areas, years or seasons being much more represented. For example, some years were only sampled in the northwest Atlantic area. Therefore, we chose to combine all the data to ensure we had enough data without considering a temporal effect in the models. However, temporality was taken into account when associating environmental conditions with effort segments, so when there was a sighting, it was associated with environmental conditions averaged over the week for the SEAPODYM variables and over the month for the environmental variables before the sighting, to take into account potential time-lags [11,12,13]. When deep-diver data will be large enough to allow a more uniform temporal sampling, the temporal aspect will be taken into account in the models.

We took care in using appropriate statistical tools for modelling the habitat of species with few sightings [71]. Virgili et al. [71] showed that GAMs with a Tweedie distribution generated reliable habitat modelling predictions for rarely sighted marine predators. We constrained the number of variables in the models to a maximum of 4 (ensuring that they were all significant) to avoid excessive

complexity of models and difficulty in their interpretation [53]. Although this could limit the performance of our models, we were confident in the procedure parsimony. Indeed, the explained deviances obtained (between 20 and 40%) were high compared to other studies on cetaceans [63,68], suggestive of a good fit to the data, and for each model, only four or five variables were actually important, the others representing a very small percentage of the Akaike weights (S3.2 Fig.). As a result, four variable models were the best compromise between avoidance of overfitting, reduction of complexity and maximisation of interpretability.

Ability of SEAPODYM and environmental variables to model deep-diver distributions

The model comparison showed that for beaked whales, the explained deviance was higher and the ~~AIC~~ RMSE was lower in the ENVIRONMENTAL model than in the SEAPODYM model, so environmental variables seemed to better explain their distribution than SEAPODYM variables; this could explain why no SEAPODYM variable was selected in the COMBINED model (most important variables for the beaked whale COMBINED model were only environmental variables; S3.2 Fig.). As for Torres et al. [72], it is possible that the integration of prey data did not improve the explanatory power of the models because the prey sampling was not appropriate or there was a mismatch between the distribution of prey and predators because they respond to oceanographic processes at different spatial and temporal scales. The link between predators and the environment may therefore be simpler to model than the link between predators and prey [72] or the spatial resolution of the SEAPODYM variables (0.25°) is quite coarse to depict specific associations between an animal and its environment. In contrast, for sperm whales, the ENVIRONMENTAL and COMBINED models were comparable but the explained deviance and the ~~AIC~~RMSE were slightly better for the COMBINED model so SEAPODYM variables seemed to provide additional information to that provided by the environmental variables alone. The production of epipelagic organisms appeared to be one of the five most important variables in the sperm whale COMBINED model (S3.2 Fig.). These results agreed with

those of Hazen et al. [16] who showed that the performance of a model which included static (depth), oceanographic (salinity and temperature) and biological (deep scattering layer and number of available prey) variables was higher than that of the model which only included prey data. One hypothesis, suggested by Hazen et al. [16], would be that beaked whales do not necessarily track a particular type of prey but rather large-scale physiographic structures while sperm whales would be more pelagic predators, so the prey simulated by SEAPDOYM would correspond more closely to the prey targeted by sperm whales than by beaked whales. Another hypothesis could be a temporal mismatch between SEAPODYM output and the growth rate of the actual prey of these species as discussed by Chambault et al. [73].

~~However, t~~The prey functional groups that were simulated from SEAPODYM may not directly reflect the prey targeted by deep-divers. The three biological layers of SEAPODYM are defined between 0 and *ca.* 1,000 m with organisms ranging from 2 to 20 cm [44,45], but deep-divers can feed beyond 1,000 m on prey bigger than 20 cm, mostly beaked whales [31,74,75]. From stomach contents, Spitz et al. [31] showed that the diet of Cuvier's beaked whales consisted largely of cephalopods bigger than 20 cm, *Teuthowenia megalops* (21,2 ± 3,3 cm) and *Galiteuthis armata* (24,2 ± 2,4 cm) and the diet of sperm whales was mostly composed of cephalopods close to 20 cm, *Histioteuthis bonnellii* (17,8 ± 3,7 cm) in the north-east Atlantic Bay of Biscay. Thus, the functional groups of preys simulated in SEAPODYM may represent only a part of the prey ~~not be directly~~ targeted by deep-divers ~~but~~ and the probable link could be mediated by deep-diver prey instead. The prey simulated in SEAPODYM ~~could~~ may correspond more closely to the prey of the prey targeted by deep-divers. A similar phenomenon was observed in the study of Putra & Mustika [66] which showed that the zooplankton biomass was a strong predictor of the distribution of manta rays (*Mobula birostris*) because they feed directly on zooplankton whereas it was not a strong predictor for dolphins (*Stenella* spp.) because they are second- and third-level consumers. Nevertheless, the study shed light on dolphin foraging habits. It is likely the same situation in our study, the prey targeted by deep-divers may be larger than those simulated in SEAPODYM [31], therefore the simulated trophic levels may be lower than those actually

targeted by deep-divers. This may explain why epipelagic organisms were retained in the sperm whale models, many bathypelagic squids and fishes are vertically migrating predators feeding at night in the epipelagic layer. In addition, the functional groups simulated by SEAPODYM encompass multiple organisms (fishes, cephalopods, crustaceans; [44,45]), so deep-diver preys, which are mainly cephalopods, might be poorly represented in the functional groups in terms of biomass and production, even in upper and lower mesopelagic ~~and bathypelagic~~ layers, explaining why they were not selected in the COMBINED models.

~~The combination of sighting data from different ecosystems (*e.g.* North-East and North-West Atlantic) may also mask inter-regional differences in the relationships between cetacean densities and the environmental predictors, as shown by Mannocci et al. [74]. However, the objective here, was to compare models using environmental variables, prey distribution variables, and a combination of the two types of variables to determine the extent to which the models could be improved and not necessarily explain precisely the mechanisms influencing the distribution of beaked and sperm whales, which would be more consistent at the scale of a smaller region.~~Although it would be more consistent to fit models at a regional scale, we highlighted interesting relationships with the environment and/or the simulated prey at a large scale. ~~The highest relative densities in the ENVIRONMENTAL models were associated with deep depths, strong spatial gradients of SST and gentle slopes for both beaked whales and sperm whales, and also with the presence of canyons and seamounts for beaked whales and with high EKE for sperm whales.~~ Relationships obtained in the ENVIRONMENTAL models ~~This~~ suggested~~s~~ a preference for habitats associated with topographic features and thermal fronts for both species, as previously shown [27,32,33]. Comparatively, SEAPODYM variables reveal areas of prey concentration whereas environmental variables are only indirect proxies of the prey distribution. Putra & Mustika [66] suggested that chlorophyll *a* concentrations are not spatially correlated to high zooplankton biomasses because of the time lag between phytoplankton development and meso-zooplankton growth, therefore dolphin (*Stenella* spp.) densities would be higher where zooplankton biomasses would be higher but where chlorophyll *a* concentrations would be lower. SEAPODYM variables could

thus give a better idea of the foraging areas used by the species [19,23,45]. We found that beaked whales were closely related to ~~low~~ quite high biomass~~ses~~ of lower meso~~bathy~~pelagic organisms, low biomass of ~~epipelagic and~~ migrant upper mesopelagic organisms, low production of migrant lower mesopelagic organisms and deep euphotic depth. This would suggest a preference for habitats where the euphotic zone is extended and with large lower mesopelagic prey but not a preference for large upper mesopelagic organisms, which could partly correspond to the prey targeted by beaked whales [31]. ~~, while s~~Sperm whales were closely related to ~~low~~ quite high biomass~~es~~ of lower meso~~bathy~~pelagic organisms, low biomass of ~~and~~ epipelagic organisms, high production of epipelagic organisms and deep euphotic depth. As for beaked whales, ~~T~~this would suggest~~s~~ a preference for habitats where the euphotic zone is extended, with large lower mesopelagic prey but also abundant small epipelagic prey *i.e.* a productive habitat at the surface which allow the development of large prey at depth. This is partly consistent with the diet of sperm whales which seems more flexible than the diet of beaked whales [31]. The positive relationship with epipelagic production and the negative relationship with epipelagic biomass would indicate habitats with abundant small epipelagic prey and not large epipelagic prey, which would be consistent with the previously mentioned hypothesis that this would be the prey targeted by sperm whale prey. ~~a preference for moderately productive offshore habitats~~. The variables selected i~~I~~n the COMBINED models (or ENVIRONMENTAL model for beaked whale) ~~, no SEAPODYM variable was selected for beaked whales, *i.e.* the model was identical to the environmental model, whereas for sperm whales, two SEAPODYM variables (biomass and production of epipelagic organisms) and two environmental variables (depth and gradients of SST) were selected, suggesting a preference for habitats associated with topographic features and thermal fronts for beaked whales and sperm whales but the distribution of sperm whales would also be influenced by the biomass and production of pelagic prey~~would suggest that ~~. If~~ beaked whales were more associated ~~to~~ with physiographic features around 2000 m deep so ~~,~~ they might forage on organisms living close to the seabed that may be poorly modelled by SEAPODYM. By contrast, sperm whales would be less constrained by the presence of the slope, and would therefore prey on organisms that are truly pelagic

[76], and therefore possibly better modelled by SEAPODYM. ~~-~~The addition of other depth variables such as the bottom temperature could help clarify the relationship with depth [77]. Other variables could also be considered in the models such as SSH fronts, characteristics of the eddies, however the objective here was to compare SEAPDOYM variables to environmental surface variables commonly used and known to influence the deep-diver distribution [27,32, 33, 68].

The euphotic depth, which is the maximum depth of the light zone suitable for phytoplankton photosynthesis, was selected for both beaked whales and sperm whales in the SEAPODYM models with ~~maximum densities predicted around 65 m for beaked whales while for sperm whales we obtained an~~ increasing relationships ~~with the euphotic depth. This suggests that beaked whales would be more likely to be found in continental slope areas with a moderate depth of the euphotic zone (50-70 m; see S2 Figs.), whereas sperm whales would be more dispersed offshore.~~ The euphotic depth was a variable available in SEAPODYM but the mixed layer depth would probably be more informative for deep-divers because it provides information on the depth of the thermocline which is frequently used by diving animals [76], it would therefore be interesting to explore the differences in the relationship between these two types of variables for deep-divers.

For both beaked and sperm whales, depth was selected in the ~~COMBINED~~ models. There is a great interest in including depth in the models because it is a well-measured variable, *i.e.* with low associated measurement error, unlike remote sensed or modelled variables which . ~~Physiographic variables such as depth, because measured with little error compared to other variables in the model,~~ may help ~~in~~ improving the explanatory power of the models. Pendleton et al. [78] observed similar results for bowhead whales (*Balaena mysticetus*). They compared a model using only environmental and biological variables such as sea ice thickness, sea temperature, diatoms, flagellates, copepods and zooplankton from the Biology Ice Ocean Modeling and Assimilation System (BIOMAS) to a model using these variables plus bathymetry and showed that the best model included bathymetry and BIOMAS variables. The importance of bathymetry suggests there is probably a close relationship between bathymetry and the biophysical processes that determine the distribution of these cetaceans.

Overall, no beaked whales and sperm whales were predicted on the continental shelf because no sightings were recorded, which confirms the non-suitability of this habitat for these species [32,33]. The bathymetry greatly influenced the beaked and sperm whale distributions in the ENVIRONMENTAL and COMBINED models, with the highest densities predicted near slope discontinuities where the bottom is steep and where prey aggregate [79,80]. A west-east density gradient was also observed for both species, which is consistent with the studies of Roberts et al. [32] and Rogan et al. [33]. Sperm whales, appeared to be less linked to these structures than beaked whales and their distribution was slightly more homogeneous. With SEAPODYM variables, predicted densities were lower and prediction maps were smoother (a common scale was applied to facilitate the comparison). Beaked and sperm whales seemed homogeneously distributed in oceanic waters, which was not very consistent with the sightings and highlighted the poorer performance of the models (higher RMSEs). As previously showed in Lambert et al. [21], these smoother predictions This may be due to the large resolution of the variables. .The spatial resolution of the SEAPODYM variables is currently set to 0.25° but is planned to be refined to 0.125° and a finer spatial resolution may reduce this smooth effect. However, by adding environmental variables, particularly depth, in the sperm whale COMBINED model, the model performance was increased (higher explained deviance and lower RMSE), and particularly depth, in the sperm whale COMBINED model, the prediction was more spatially detailed with highest densities predicted in the western Atlantic. .prediction maps of species relative densities were similar between the three models for beaked and sperm whales with highest densities predicted offshore along continental slopes where prey aggregate [64,65], but prediction maps were smoother with SEAPODYM variables than with environmental variables, as previously showed in Lambert et al. [20]. This may be due to the large resolution of the variables. The spatial resolution of the SEAPODYM variables is currently set to 0.25° but is planned to be refined to 0.125° and a finer spatial resolution may reduce this smooth effect. However, by adding environmental variables, particularly depth, in the sperm whale COMBINED model, the prediction was more spatially detailed.

We performed a gap analysis to highlight geographical extrapolation areas in which environmental and SEAPODYM variables were out ranges of surveyed conditions. The centre of the study area, near the Mid-Atlantic Ridge, was not surveyed but variable conditions remained within the ranges of surveyed conditions, allowing to predict distribution at a large scale. The gap analysis revealed large gaps in environmental space coverage across the study area, especially in the western part of the Atlantic Ocean for the ENVIRONMENTAL models and in tropical waters for the SEAPODYM models. This suggests that sampling effort was not sufficient in deeper and steeper areas and more intensive sampling effort performed in these areas could help better describe the habitat used by deep-divers. The interpolation areas varied between the different models, from 74/82% for the SEAPODYM models to 84%/85% for the ENVIRONMENTAL and COMBINED models, which means that 18 and 26% of the area represented an environmental extrapolation with the SEAPODYM models, compared to only 16% with the COMBINED models for sperm whales and 15% in the ENVIRONMENTAL model for beaked whales. The uncertainties were thus greater with the SEAPDYM models and it appeared riskier to perform models at a large scale with SEAPODYM variables only, than with environmental variables. For sperm whales~~However~~, by combining both types of variables, this risk was ~~greatly~~ slightly reduced compared to the SEAPODYM model~~s~~ ~~.~~ and ~~Thus,~~ areas of ~~species~~ concentration seemed more spatially detailed and better characterised with the COMBINED model~~s~~, as revealed by a lower RMSE. To meet ~~species~~ sperm whale conservation objectives, it seems more valuable to use models combining environmental variables and prey distribution data. Habitat models are regularly used to identify areas where species concentrate in order to set up protection areas [81-83]. They are also useful for predicting species distribution in non-sampled or poorly documented areas [84-85], provided that areas of interpolation and extrapolation are considered [27,57,58]. Considering key habitats of species, particularly foraging habitats, in the design of marine protected areas thanks to the combination of environmental and prey variables (with further development to include prey of all types of predators) would ~~thus~~ represent an improvement in the tools available for

species conservation, and consideration could be given to reducing the overlap between impacting human activities and these key habitats [86,87].

Conclusion

Prey distribution data are not widely available to model the distribution of marine top predators. However, there are models, still not widely used, such as SEAPODYM, which simulate the distribution of functional groups of prey. SEAPODYM outputs alone did not seem ideal for modelling prey of deep-diving cetaceans and maybe SEAPODYM was ruled out as a good framework for describing the distribution of beaked and sperm whales. However, by combining SEAPODYM and environmental variables, we slightly increased the sperm whale model performance and were able to well define the areas of concentration. In contrast, for beaked whales, combining environmental and SEAPODYM variables did not improved the model performance, no SEAPDOYM variables were selected in the best model, possibly because they prey on organisms more associated to seabed features and consequently less well predicted by SEAPODYM that focus on pelagic prey assemblages. This study was a first investigation and w~~We~~ mostly highlighted the importance of the physiographic variables to understand ~~static~~ mechanisms that influence the distribution of deep-diving cetaceans. To improve the results, it would be interesting to refine spatial resolution of the variables, to fit region-specific models or to apply this approach to species whose functional groups of prey would be better represented in SEAPODYM such as baleen whales or small delphinids. The development of SEAPODYM~~,~~ ~~in particular the increase in spatial resolution of the variables and the inclusion of measurement errors in SEAPODYM variables,~~ could make SEAPODYM an essential data source to help improving knowledge on species and to be included in management procedures that aimed at improving the conservation of emblematic species such as cetaceans.~~Our approach showed the possibility of improving the explanatory power of habitat-based density models by using a combination of physiographic and oceanographic variables and variables that estimate expected prey availability. The areas of sperm~~

whale concentrations seemed better explained by the model that combined both types of variables. This was not the case for beaked whales, possibly because they prey on organisms more associated to seabed features and consequently less well predicted by SEAPODYM that focus on pelagic prey assemblages. We highlighted the importance of the physiographic variables to understand static mechanisms that influence the distribution of deep-diving cetaceans. The use of more direct estimates of prey availability, rather than oceanographic proxies, could be important for predicting species distributions because the relationships between proxies and prey may change in a climate change context.

Although SEAPODYM outputs alone did not seem ideal for modelling prey of deep-diving cetaceans, we were able to better define the areas of concentration of these species. To improve these results, it would be interesting to fit region-specific models or to apply this approach to species whose functional groups of prey would be better represented in SEAPODYM such as baleen whales or small delphinids. The development of SEAPODYM, in particular the increase in spatial resolution of the variables and the inclusion of measurement errors in SEAPODYM variables, could make SEAPODYM an essential data source to help improving knowledge on species and to be included in management procedures that aimed at improving the conservation of emblematic species such as cetaceans.

Acknowledgments

We are grateful to the many observers who participated in the surveys and collected all the data but also the ships' captains, crews and pilots. We thank Phil Hammond and his team for providing SCANS and CODA survey data. THUNNUS survey was carried out thanks to the collaboration of the General Directorate of Fisheries and Maritime Affairs, Government of Galicia. We thank the *Direction Générale de l'Armement* (DGA), including Odile Gérard and Carole Nahum, for their support during this study. We thank the *Direction Générale de l'Armement* (DGA), including Odile Gérard and Carole Nahum, for funding Auriane Virgili's doctoral research grant. ML was funded by a Ramón y Cajal (RYC-

~~2012-09897) postdoctoral contract of the Spanish Ministry of Economy, Industry and Competitiveness. This study is a contribution to the CHALLENGES (CTM2013-47032-R) project of the Spanish Ministry of Economy, Industry and Competitiveness.~~ We warmly thank Charlotte Lambert for her help, support and comments. We thank Philippine Chambault and the two other anonymous referees, for their very helpful comments that led to a clearer and much improved manuscript.

Data accessibility

All sighting and effort data used in this study are available in the OBIS SEAMAP database: http://seamap.env.duke.edu/. All data providers can be contacted via the OBIS SEAMAP website.

References

[1] Schipper J, Chanson J.S, Chiozza F, Cox N.A, Hoffmann M, Katariya V, et al. 2008. The Status of the World's Land and Marine Mammals: Diversity, Threat, and Knowledge. Science, 322: 225–230.

[2] Avila I.C, Kaschner K, Dormann C.F. 2018. Current global risks to marine mammals: Taking stock of the threats. Biological Conservation, 221: 44-58.

[3] Panti C, Baini M, Lusher A, Hernandez-Milan G, Rebolledo E.L.B, Unger B, et al. 2019. Marine litter: one of the major threats for marine mammals. Outcomes from the European Cetacean Society workshop. Environmental pollution, 247: 72-79.

[4] Elith J, & Leathwick J.R. 2009. Species distribution models: ecological explanation and prediction across space and time. Annual review of ecology, evolution, and systematics, 40: 677-697.

[5] Guisan A, & Zimmermann N.E. 2000. Predictive habitat distribution models in ecology. Ecological modelling, 135(2-3): 147-186.

[6] Yen P.P, Sydeman W.J, Hyrenbach K.D. 2004. Marine bird and cetacean associations with bathymetric habitats and shallow-water topographies: implications for trophic transfer and conservation. Journal of Marine systems, 50(1-2): 79-99.

[7] Redfern J.V, Ferguson M.C, Becker E.A, Hyrenbach K.D, Good C, Barlow J, et al. 2006. Techniques for cetacean – habitat modeling. Marine Ecology Progress Series, 310: 271–295.

[8] Lambert C, Laran S, David L, Dorémus G, Pettex E, Van Canneyt O, Ridoux V. 2017. How does ocean seasonality drive habitat preferences of highly mobile top predators? Part I: the north-western Mediterranean Sea. Deep Sea Research Part II: Topical Studies in Oceanography, 141: 115-132.

[9] Jaquet N, & Whitehead H. 1996. Scale-dependent correlation of sperm whale distribution with environmental features and productivity in the South Pacific. Marine ecology progress series: 1-9.

[10] Ferguson M.C, Barlow J, Reilly S.B, Gerrodette T. 2006. Predicting Cuvier's (*Ziphius cavirostris*) and Mesoplodon beaked whale population density from habitat characteristics in the eastern tropical Pacific Ocean. Journal of Cetacean Research and Management, 7: 287–299.

[11] Grémillet D, Lewis S, Drapeau L, van Der Lingen C. D, Huggett J. A., Coetzee, J. C, et al. 2008. Spatial match–mismatch in the Benguela upwelling zone: should we expect chlorophyll and sea-surface temperature to predict marine predator distributions? Journal of Applied Ecology, 45(2): 610-621.

[12] Austin D, Bowen W.D, McMillan J.I, Iverson S.J. 2006. Linking Movement, Diving, and Habitat to Foraging Success in a Large Marine Predator. Ecology, 87: 3095–3108.

[13] Cotté C, Guinet C, Taupier-Letage I, Mate B, Petiau E. 2009. Scale-dependent habitat use by a large free-ranging predator, the Mediterranean fin whale. Deep Sea Research Part I: Oceanographic Research Papers, 56: 801–811.

[14] Croll D.A, Marinovic B, Benson S, Chavez F.P, Black N, Ternullo R, et al. (2005). From wind to whales: trophic links in a coastal upwelling system. Marine Ecology Progress Series, 289: 117-130.

[15] Österblom H, Olsson O, Blenckner T, Furness R.W. 2008. Junk-food in marine ecosystems. Oikos, 117: 967–977.

[16] Hazen E.L, Nowacek D.P, Laurent L.S, Halpin P.N, Moretti D.J. 2011. The relationship among oceanography, prey fields, and beaked whale foraging habitat in the Tongue of the Ocean. PloS one, 6(4): e19269.

[17] Louzao M, Valeiras J, García-Barcelona S, González-Quirós R, Nogueira E, Iglesias M, et al. 2019. Marine megafauna niche coexistence and hotspot areas in a temperate ecosystem. Continental Shelf Research, 186: 77-87.

[18] Rockwood R.C, Elliott M.L, Saenz B, Nur, N, Jahncke J. 2020. Modeling predator and prey hotspots: Management implications of baleen whale co-occurrence with krill in Central California. PloS one, 15(7): e0235603.

[19] Lehodey P, Murtugudde R, Senina I. 2010. Bridging the gap from ocean models to population dynamics of large marine predators: a model of mid-trophic functional groups. Progress in Oceanography, 84(1): 69-84.

[20] Abecassis M, Senina I, Lehodey P, Gaspar P, Parker D, Balazs G, et al. 2013. A model of loggerhead sea turtle (*Caretta caretta*) habitat and movement in the oceanic North Pacific. PLoS One, 8(9): e73274.

[21] Lambert C, Mannocci L, Lehodey P, Ridoux V. 2014. Predicting cetacean habitats from their energetic needs and the distribution of their prey in two contrasted tropical regions. PloS one, 9(8): e105958.

[22] Green D.B, Bestley S, Trebilco R, Corney S.P, Lehodey P, McMahon C.R, et al. 2020. Modelled mid-trophic pelagic prey fields improve understanding of marine predator foraging behaviour. Ecography, 43:1-13.

[23] Lehodey P, Senina I, Murtugudde R. 2008. A spatial ecosystem and populations dynamics model (SEAPODYM) – Modeling of tuna and tuna-like populations. Progress in Oceanography, 78(4): 304-318.

[24] Briand K, Molony B, Lehodey P. 2011. A study on the variability of albacore (*Thunnus alalunga*) longline catch rates in the southwest Pacific Ocean. Fisheries Oceanography, 20(6): 517-529.

[25] Pérez-Jorge S, Tobeña M, Prieto R, Vandeperre F, Calmettes B, Lehodey P, et al. 2020. Environmental drivers of large-scale movements of baleen whales in the mid-North Atlantic Ocean. Diversity and Distributions, 26: 683–698.

[26] Chambault P, de Thoisy B, Heerah K, Conchon A, Barrioz S, Dos Reis V, et al. (2016). The influence of oceanographic features on the foraging behavior of the olive ridley sea turtle *Lepidochelys olivacea* along the Guiana coast. Progress in Oceanography, 142: 58-71.

[27] Virgili A, Authier M, Boisseau O, Cañadas A, Claridge D, Cole T, et al. 2019. Combining multiple visual surveys to model the habitat of deep-diving cetaceans at the basin scale. Global Ecology and Biogeography, 28(3): 300-314.

[28] Fernández A, Edwards J.F, Rodríguez F, Espinosa de los Monteros A, Herráez P, Castro, P., el al. (2005). "Gas and fat embolic syndrome" involving a mass stranding of beaked whales (family *Ziphiidae*) exposed to anthropogenic sonar signals. Veterinary Pathology, 42: 446–457.

[29] D'Amico A, Gisiner R.C, Ketten D.R, Hammock J.A, Johnson C, Tyack P.L, et al. (2009). Beaked whale strandings and naval exercises. Aquatic Mammals, 35: 452–472.

[30] Perrin W.F, Würsig B, Thewissen J.G.M. (Eds.). 2009. Encyclopedia of marine mammals. Academic Press.

[31] Spitz J, Cherel Y, Bertin S, Kiszka J, Dewez A, Ridoux V. 2011. Prey preferences among the community of deep-diving odontocetes from the Bay of Biscay, Northeast Atlantic. Deep Sea Research I, 58: 273–282.

[32] Roberts J.J, Best B.D, Mannocci L, Fujioka E, Halpin P.N, Palka D.L, et al. 2016. Habitat-based cetacean density models for the U.S. Atlantic and Gulf of Mexico. Scientific Report, 6.

[33] Rogan E, Cañadas A, Macleod K, Santos M.B, Mikkelsen B, Uriarte A, et al. 2017. Distribution abundance and habitat use of deep diving cetaceans in the North-East Atlantic. Deep Sea Research Part II: Topical Studies in Oceanography, 141: 8–19.

[34] Levin L.A, & Gooday A. 2003. The Deep Atlantic Ocean. Ecosystems of the deep oceans. (Tyler PA, Ed.). pp. 111-178. Amsterdam; New York: Elsevier.

[35] Tomczak M, & Godfrey J.S. 2003. Regional oceanography: an introduction. Elsevier.

[36] Campbell J.W, & Aarup T. 1992. New production in the North Atlantic derived from seasonal patterns of surface chlorophyll. Deep Sea Research Part A. Oceanographic Research Papers, 39: 1669–1694.

[37] ESRI. 2016. ArcGIS – A complete integrated system. Redlands, CA: Environmental Systems Research Institute Inc. Retrieved from http://esri.com/arcgis.

[38] Roberts J.J, Best B.D, Dunn D.C, Treml E.A, Halpin P.N. 2010. Marine geospatial ecology tools: An integrated framework for ecological geoprocessing with ArcGIS, Python, R, MATLAB, and C++. Environmental Modelling and Software, 25: 1197–1207.

[39] Buckland S.T, Rexstad E.A, Marques T.A, Oedekoven C.S. 2015. Distance sampling: methods and applications. Springer.

[40] Bivand R.S. & Wong D.W.S (2018) Comparing implementations of global and local indicators of spatial association TEST, 27(3), 716-748. Retrieved from https://doi.org/10.1007/s11749-018-0599-x.

[41] Harris P.T, Macmillan-Lawler M, Rupp J, Baker E.K. 2014. Geomorphology of the oceans. Marine Geology, 352: 4–24.

[42] Canada Meteorological Center. 2012. GHRSST level 4 CMC0.2deg global foundation sea surface temperature analysis (GDS version 2). Ver. 2.0. PO.DAAC. Pasadena, CA.

[43] Lehodey P, Andre J.M, Bertignac M, Hampton J, Stoens A, Menkès C, et al. 1998. Predicting skipjack tuna forage distributions in the equatorial Pacific using a coupled dynamical bio-geochemical model. Fisheries Oceanography, 7(3-4): 317-325.

[44] Conchon A. 2016. Modeling marine zooplankton and micronekcton (Doctoral dissertation, La Rochelle University).

[45] Lehodey P, Conchon A, Senina I, Domokos R, Calmettes B, Jouanno J, et al. 2014. Optimization of a micronekton model with acoustic data. ICES Journal of Marine Science, 72(5): 1399-1412.

[46] Behrenfeld M.J, & Falkowski P.G. 1997. A consumer's guide to phytoplankton primary productivity models. Limnology and Oceanography, 42: 1479–1491.

[47] Hays G.C. 2003. A review of the adaptive significance and ecosystem consequences of zooplankton diel vertical migrations. In Migrations and Dispersal of Marine Organisms (pp. 163-170). Springer, Dordrecht.

[48] Hastie T & Tibshirani R. 1986. Generalized Additive Models. Statistical Science, 3: 297-313.

[49] Wood S.N. 2006. On confidence intervals for generalized additive models based on penalized regression splines. Australian & New Zealand Journal of Statistics, 48: 445–464.

[50] Foster S.D, & Bravington M.V. 2013. A Poisson-Gamma model for analysis of ecological non-negative continuous data. Environmental and ecological statistics, 20: 533–552.

[51] Wood S. 2013. mgcv: Mixed GAM Computation Vehicle with GCV/AIC/REML smoothness estimation. Retrieved 7 July 2014, from http://cran.r-project.org/web/packages/mgcv/index.html.

[52] Hedley S.L, & Buckland S.T. 2004. Spatial models for line transect sampling. Journal of Agricultural, Biological, and Environmental Statistics, 9(2): 181–199.

[53] Mannocci L, Catalogna M, Dorémus G, Laran S, Lehodey P, Massart W, et al. 2014. Predicting cetacean and seabird habitats across a productivity gradient in the South Pacific gyre. Progress in Oceanography, 120: 383–398.

[54] Burnham K.P, & Anderson D.R. 2002. *Model selection and multimodel inference*, 2nd edn. Springer, New York.

[55] Spiess A. 2014. qpcR: Modelling and analysis of real-time PCR data. R package version 1.4-0. Retrieved from https://CRAN.R-project.org/package=qpcR.

[56] Symonds M.R, & Moussalli A. (2011). A brief guide to model selection, multimodel inference and model averaging in behavioural ecology using Akaike's information criterion. Behavioral Ecology and Sociobiology, 65(1): 13-21.

[57] Jennings M.D. 2000. Gap analysis: Concepts, methods, and recent results. Landscape Ecology, 15: 5–20.

[58] Mannocci L, Roberts J.J, Halpin P.N, Authier M, Boisseau O, Bradai M.N, et al. 2018. Assessing cetacean surveys throughout the Mediterranean Sea: A gap analysis in environmental space. Scientific Reports, 8(1): 3126.

[59] Bouchet P.J, Miller D.L, Roberts J.J, Mannocci L, Harris C.M, Thomas L. 2019. From here and now to there and then: Practical recommendations for extrapolating cetacean density surface models to novel conditions. Technical report 2019-01 v1.0, Centre for Research into Ecological & Environmental Modelling (CREEM), University of St Andrews, 59 p.

[60] Stoll H, King G, Zeng L. 2014. WhatIf: Software for evaluating counterfactuals. R package version 1.5-6. Retrieved from https:// cran.r-project.org/web/packages/WhatIf/index.html.

[61] Authier M, Saraux C, Péron C. 2016. Variable selection and accurate predictions in habitat modelling: A shrinkage approach. Ecography, 40: 549–560.

[62] King G, & Zeng L. 2007. When can history be our guide? The pitfalls of counterfactual inference. International Studies Quarterly, 51: 183–210.

[63] Becker E.A, Forney K.A, Thayre B.J, Debich A.J, Campbell G.S, Whitaker K, et al. (2017). Habitat-based density models for three cetacean species off Southern California illustrate pronounced seasonal differences. Frontiers in Marine Science, 4:121.

[64] Neill S.P, & Hashemi M.R. (2018). *Fundamentals of ocean renewable energy: generating electricity from the sea*. Academic Press, pp 193-235.

[65] Becker E.A, Carretta J.V, Forney K.A, Barlow J, Brodie S, Hoopes R, et al. (2020). Performance evaluation of cetacean species distribution models developed using generalized additive models and boosted regression trees. Ecology and evolution, 10(12): 5759-5784.

[66] Putra M.I.H, & Mustika P.L.K. 2020. Incorporating in situ prey distribution into foraging habitat modelling for marine megafauna in the Solor waters of the Savu Sea, Indonesia. Aquatic Conservation: marine and freshwater ecosystems, 30(12): 2384-2401.

[67] Stone C.J, & Tasker M.L (2006). The effects of seismic airguns on cetaceans in UK waters. Journal of Cetacean Research and Management, 8 : 255–263.

[68] Cañadas A, de Soto N.A, Aissi M, Arcangeli A, Azzolin M, B-Nagy A., et al. 2018. The challenge of habitat modelling for threatened low density species using heterogeneous data: The case of Cuvier's beaked whales in the Mediterranean. Ecological Indicators, 85: 128–136.

[69] Brodie S, Jacox M.G, Bograd S.J, Welch H, Dewar H, Scales K.L, et al. (2018). Integrating dynamic subsurface habitat metrics into species distribution models. Frontiers in Marine Science, 5: 219.

[70] Mannocci L, Roberts J.J, Pedersen E.J, Halpin P.N. 2020. Geographical differences in habitat relationships of cetaceans across an ocean basin. Ecography, 43:1-10

[71] Virgili A, Authier M, Monestiez P, Ridoux V. (2018). How many sightings to model rare marine species distributions. PLoS ONE, 13(3): e0193231.

[72] Torres L.G, Read A.J, Halpin P. 2008. Fine-scale habitat modeling of a top marine predator: Do prey data improve predictive capacity. Ecological Applications, 18(7): 1702-1717.

[73] Chambault P, Roquet F, Benhamou S, Baudena A, Pauthenet E, de Thoisy B, et al. (2017). The Gulf Stream frontal system: A key oceanographic feature in the habitat selection of the leatherback turtle? Deep Sea Research Part I: Oceanographic Research Papers, 123: 35-47.

[74] Watwood S.L, Miller P.J, Johnson M, Madsen P.T, Tyack P.L. 2006. Deep-diving foraging behaviour of sperm whales (*Physeter macrocephalus*). Journal of Animal Ecology, 75(3): 814-825.

[75] Madsen P.T, de Soto N.A, Tyack P.L., Johnson M. 2014. Beaked whales. Current Biology, 24(16): 728–730.

[76] Irvine L, Palacios D.M, Urbán J, Mate B. (2017). Sperm whale dive behavior characteristics derived from intermediate-duration archival tag data. Ecology and evolution, 7(19): 7822-7837.

[77] Chambault P, Fossette S, Heide-Jørgensen M.P, Jouannet D, Vély M. (2021). Predicting seasonal movements and distribution of the sperm whale using machine learning algorithms. Ecology and evolution, 11(3): 1432-1445.

[78] Pendleton D.E, Holmes E.E, Redfern J, Zhang J. 2020. Using modelled prey to predict the distribution of a highly mobile marine mammal. Diversity and Distributions, 26(11): 1612-1626.

[79] Bost C.A, Cotté C, Bailleul F, Cherel Y, Charrassin J.B, Guinet C, et al. 2009. The importance of oceanographic fronts to marine birds and mammals of the southern oceans. Journal of Marine Systems, 78: 363–376.

[80] Woodson C.B. & Litvin S.Y. 2015. Ocean fronts drive marine fishery production and biogeochemical cycling. Proceedings of the National Academy of Sciences, 112(6): 1710–1715.

[81] Cañadas A, Sagarminaga R, De Stephanis R, Urquiola E, Hammond P.S. 2005. Habitat preference modelling as a conservation tool: Proposals for marine protected areas for cetaceans in southern Spanish waters. Aquatic Conservation: Marine and Freshwater Ecosystems, 15(5): 495–521.

[82] McShea W.J. 2014. What are the roles of species distribution models in conservation planning? Environmental Conservation, 41: 93–96.

[83] Hazen E.L, Palacios D.M, Forney K.A, Howell E.A, Becker E, Hoover A.L, et al. 2017. WhaleWatch: A dynamic management tool for predicting blue whale density in the California Current. Journal of Applied Ecology, 54(5): 1415–1428.

[84] Fleishman E, Nally R.M, Fay J.P, Murphy D.D. 2001. Modeling and predicting species occurrence using broad-scale environmental variables: An example with butterflies of the Great Basin. Conservation Biology, 15(6): 1674–1685.

[85] Lumaret J.P, & Jay-Robert P. 2002. Modelling the species richness distribution of French dung beetles (*Coleoptera, Scarabaeidae*) and delimiting the predictive capacity of different groups of explanatory variables. Global Ecology and Biogeography, 11(4): 265–277.

[86] Thaxter C.B, Lascelles B, Sugar K, Cook A.S, Roos S, Bolton M, et al. 2012. Seabird foraging ranges as a preliminary tool for identifying candidate Marine Protected Areas. Biological Conservation, 156: 53–61.

[87] Young H.S, Maxwell S.M, Conners M.G, Shaffer S.A. 2015. Pelagic marine protected areas protect foraging habitat for multiple breeding seabirds in the central Pacific. Biological Conservation, 181: 226–235.

Supporting information

S1 Table. Details of surveys used in the analyses. Total effort represents the total length of transects of each survey (without removing the transects with a Beaufort sea-state > 4). NE-ATL: Northeast Atlantic Ocean; NW-ATL: Northwest Atlantic Ocean.

S2 File. Average conditions of the static, oceanographic and SEAPODYM variables over the entire period (from 1998 to 2015). Base map from https://www.gebco.net/.

S3 File. Correlations between environmental and SEAPODYM variables and model outputs for beaked and sperm whales.

S4 Fig. Uncertainty maps representing the standard error associated with the predicted relative density of beaked (BW) and sperm (SW) whales. Black areas represent extrapolation where we did not extrapolate the predictions. Base map from https://www.gebco.net/.

Auriane Virgili
La Rochelle University, France
auriane.virgili@univ-lr.fr

Dear Editor,

Thank you for considering our article entitled “Towards a better characterisation of deep-diving whales’ distributions by using prey distribution model outputs”, for publication in the journal PLOS ONE. We have taken into account all comments from the editor and the three reviewers regarding the article.

We thank the reviewers for their careful reading of our manuscript and for pointing out elements in the abstract, methods and discussion that needed to be more developed. We did not change the models used in our study, as generalized additive models have repeatedly shown their effectiveness in modelling the distribution of cetaceans (*e.g.* Roberts et al., 2016; Becker et al., 2017; Cañadas et al., 2018; Virgili et al., 2019) but we have completed the methodology to make it more robust, we have changed the temporal resolution of the SEAPODYM variables so that the link between species distribution and these variables would be more direct, we have modified the process of variable selection for the COMBINED models, and we have added a new parameter for estimating the model performance. Many discussion points have been completed and added in order to meet the expectations of the reviewers.

We have fixed all typos and grammatical mistakes during the revision and ensured that the manuscript meets the PLOS ONE’s style requirements.

We hope that the revised manuscript addresses the different concerns raised by the editor and the reviewers, whom we thank for their careful reading and review of this manuscript. We feel the revised manuscript has improved as a result and hope it will be deemed acceptable for consideration of publication in PLOS ONE.

Sincerely,

On behalf of all co-authors,

Auriane Virgili

Concerning specific Editor and reviewer's comments:

Editor's comments:

1. Please ensure that your manuscript meets PLOS ONE's style requirements, including those for file naming.

ANSWER: We have checked all the requirements provided on the website and modified the manuscript to meet these requirements.

2. Thank you for stating the following in the Competing Interests section:

'The authors have declared that no competing interests exist.'

We note that one or more of the authors are employed by a commercial company: ADERA

a. Please provide an amended Funding Statement declaring this commercial affiliation, as well as a statement regarding the Role of Funders in your study. If the funding organization did not play a role in the study design, data collection and analysis, decision to publish, or preparation of the manuscript and only provided financial support in the form of authors' salaries and/or research materials, please review your statements relating to the author contributions, and ensure you have specifically and accurately indicated the role(s) that these authors had in your study. You can update author roles in the Author Contributions section of the online submission form.

Please also include the following statement within your amended Funding Statement.

"The funder provided support in the form of salaries for authors [insert relevant initials], but did not have any additional role in the study design, data collection and analysis, decision to publish, or preparation of the manuscript. The specific roles of these authors are articulated in the 'author contributions' section."

If your commercial affiliation did play a role in your study, please state and explain this role within your updated Funding Statement.

b. Please also provide an updated Competing Interests Statement declaring this commercial affiliation along with any other relevant declarations relating to employment, consultancy, patents, products in development, or marketed products, etc.

Within your Competing Interests Statement, please confirm that this commercial affiliation does not alter your adherence to all PLOS ONE policies on sharing data and materials by including the following statement: "This does not alter our adherence to PLOS ONE policies on sharing data and materials." (as detailed online in our guide for authors http://journals.plos.org/plosone/s/competing-interests). If this adherence statement is not accurate and there are restrictions on sharing of data and/or materials, please state these. Please note that we cannot proceed with consideration of your article until this information has been declared.

c. Please include both an updated Funding Statement and Competing Interests Statement in your cover letter. We will change the online submission form on your behalf.

Please know it is PLOS ONE policy for corresponding authors to declare, on behalf of all authors, all potential competing interests for the purposes of transparency. PLOS defines a competing interest as anything that interferes with, or could reasonably be perceived as interfering with, the full and objective presentation, peer review, editorial decision-making, or publication of research or non-research articles submitted to one of the journals. Competing interests can be financial or non-financial, professional, or personal. Competing interests can arise in relationship to an organization or another person. Please follow this link to our website for more details on competing interests: http://journals.plos.org/plosone/s/competing-interests.

ANSWER:

Funding statement

AV's doctoral research grant was funded by the *Direction Générale de l'Armement* (DGA).

ML was funded by a Ramón y Cajal (RYC-2012-09897) postdoctoral contract of the Spanish Ministry of Economy, Industry and Competitiveness. This study is a contribution to the CHALLENGES (CTM2013-47032-R) project of the Spanish Ministry of Economy, Industry and Competitiveness.

MA and EP are affiliated to ADERA a private business dedicated to providing support for the management of research project in public institutions in Region Nouvelle Aquitaine. In this study, ADERA solely provided support in the form of salaries for authors MA and EP, but did

not have any additional role in the study design, data collection and analysis, decision to publish, or preparation of the manuscript. The specific roles of these authors are articulated in the 'author contributions' section.

Competing interest statement

MA and EP are employed by the commercial company ADERA which did not play any role in this study beyond that of employer. This does not alter our adherence to PLOS ONE policies on sharing data and materials.

3. We note that you have indicated that data from this study are available upon request. PLOS only allows data to be available upon request if there are legal or ethical restrictions on sharing data publicly. For more information on unacceptable data access restrictions, please see http://journals.plos.org/plosone/s/data-availability#loc-unacceptable-data-access-restrictions.

ANSWER: We indicated the data from this study are available upon request because each of the co-authors of the manuscript owns part of the dataset we used and a request has been made to each of them to have access to the data so we cannot provide the dataset without their agreement. All sighting and effort data used in this study are available in the OBIS SEAMAP database: http://seamap.env.duke.edu/. Authors had no special access to the underlying data and all interested researchers can obtain the data from http://seamap.env.duke.edu in the same way that the authors obtained data by contacting the data providers via the OBIS SEAMAP website.

Email address of organisations/data providers:

AMBAR: ambarelkartea@gmail.com

AZTI: mlouzao@azti.es

BMMRO: dclaridge@bahamaswhales.org

CEMMA: cemmaorganizacion@gmail.com

IEO: Begona.SANTOS@efca.europa.eu

JNCC: tim.dunn@jncc.gov.uk

NEFSC: jason.roberts@duke.edu

PELAGIS: pelagis@univ-lr.fr

SEAPODYM: plehodey@groupcls.com

SMRU: psh2@st-andrews.ac.uk

4. We note that Appendix S2, S4 and Figures 1 and 4 in your submission contain map/satellite images which may be copyrighted.

ANSWER: All captions and figures were completed with the source of the base map (https://www.gebco.net/).

Reviewer 1 #

The article entitled "Towards a better characterisation 1 of deep-diving whales' distributions by using prey distribution model outputs" aimed at comparing 2 habitat modelling approaches to predict the distribution of deep divers using surface environmental variables vs. low and mid-trophic levels outputs. The authors showed that including SEAPODYM covariates does not improve the model performance and conversely lead to drastic different predictions, raising serious concern regarding the reliability of the model output and the algorithm used (classical additive model). Despite the efforts of the authors to provide a clear methodology, the results lack of clarity (numerous repetitions, the results are not sufficiently discussed in regards to other studies) and the bias / limitations of the modelling procedure are not well discussed. I suggested several improvements, including testing other algorithms from machine learning and adding other covariates into the model as well as additional literature. The authors have a dataset will a great potential, and I'm convinced once the methodology will be made more robust and the results clearer, this article will be of great contribution to the habitat modelling and marine mammals' communities!

ANSWER: We thank reviewer 1 for his careful reading of our manuscript and for pointing out methodological and redactional concerns. The methodology we used for the selection of variables in the models has been revised in the new version of the manuscript and a new metric has been used to evaluate the model performance in order to better meet the objectives of the study but we have not tested other algorithms as suggested. Indeed, generalized additive models have repeatedly shown their effectiveness to model cetacean distributions (*e.g.* Roberts et al., 2016; Becker et al., 2017; Cañadas et al., 2018; Virgili et al., 2019) and we believe they can also be applied in this study. Reviewer 1 proposes to use machine learning algorithms instead which is justified if the main objective is to predict the distribution of species without necessarily explaining this distribution (Gobeyn et al., 2019) but here our objective was

primarily to determine the explanatory performance of models that use different types of explanatory variables. It therefore seemed more relevant to use statistical models rather than machine learning. In addition, surface environmental variables are commonly used in generalized additive models to model the distribution of deep-divers and we wanted to assess whether the addition of prey distribution variables provided additional information to explain their distribution. Therefore, a comparable modelling approach was required. In our future studies, we will consider machine learning algorithms to predict the distribution of species. We have clarified the results and removed repetitions to meet reviewer's expectation (lines 304-366). We have more discussed bias and limitations of our study (lines 382-430, 549-557) and added more literature to discuss our study. We think the revised manuscript has improved as a result.

Specific comments:

Page 2: This result is surprising and not very intuitive. look forward reading some explanations about this; same. isn't it the opposite?; I am wondering how these prey field variables are not strongly autocorrelated, but I presume you have tested that?; more than a description of the most significant variables selected for each model, I would have expected some performance metrics (*e.g.* deviance explained) to compare the 3 models. I would have been much more informative and less descriptive to have such results in the abstract

ANSWER: We have changed the temporal resolution of the SEAPODYM variables (from 29 to 7 days) and fitted new models so the results have changed in this new version of the manuscript and we have reworked the abstract as a whole, which we hope will address the comments of reviewer 1.

Line 66: predictors? to avoid repetition

ANSWER: Changed

Line 77: also because such micronekton outputs are closer to trophic level of apex predators prey

ANSWER: Complement added in the text lines 77-78.

Line 86: you could also add this reference which shows the use of SEAPODYM to relate olive ridleys turtles to productive eddies: https://www.sciencedirect.com/science/article/abs/pii/S0079661115300094

ANSWER: Reference added line 87.

Lines 96-97: no need here to describe all the predictors you plan to use

ANSWER: Text deleted.

Line 129: the resolution of the figures is very low. I assume it is due to the requested format by the journal, but figures in vector format such as pdf would be needed to get a better result

ANSWER: Figures were provided in a format requested by the journal; the poor resolution is probably due to the creation of the pdf submission.

Line 159: dynamic or oceanographic? to be consistent pick one of these 2

ANSWER: We have changed dynamic into oceanographic in the whole text.

Line 172: you could have done the same procedure with SSH to detect ocean fronts and particularly eddies. SSH and SST fronts can be slightly different and might provide complementary variables. See Chambault et al 2017 DSR II for more information.

ANSWER: We thank the reviewer for this proposal, indeed, it is a variable that could be interesting. To our knowledge, this variable is not known to have an effect on the deep-diver distribution so we have chosen not to include it in the new version of the manuscript, we have limited ourselves to classically used variables but we will test this variable in our future studies.

Line 174: to be consistent I would suggest to use the same products as the ones assimilated into SEAPODYM. I might be wrong but I think SEAPODYM assimilates SST and currents from CMEMS directly (GLORYS product). If this is the case I would recommend to use products from CMEMS rather than many different sources of data

ANSWER: We do not believe that it makes sense to use the same data source for the environmental and SEAPODYM variables as this will necessarily lead to a correlation between the variables and therefore we would not be able to obtain a combination of the environmental and SEAPODYM variables in the COMBINED models.

Line 176: from AVISO there is also a useful product assembling all the eddies with their associated characteristics (radius, type, amplitude...): it is called the eddy Atlas. It could provide another predictor, especially since your whales seem to target the Gulf Stream

ANSWER: We thank reviewer 1 for this information but we do not think this type of variable at a very fine spatial resolution is very relevant for visual observation data, it would be more relevant for tracking data and therefore would not be applicable here.

Line 178: another dynamic variable that could be of great interest for such diving predators is the bottom temperature available on CMEMS. for these animals that dive to great depths for long periods it makes more sense to also look at vertical variables like bottom temp

ANSWER: We fully agree with reviewer 1 and the use of depth variables will be the subject of another publication. Here, the objective was to compare the SEAPODYM variables to environmental variables classically used in habitat models such as static variables and surface variables that was why we did not include bottom temperature in the models.

Line 242: you should also test the correlation using the vif function from the usdm package. the common way is to retain all variables with a vif coefficient < 4

ANSWER: As suggested by reviewer 1, we have tested the correlation with the VIF function, the results were almost identical as the correlation matrices calculated with this method and our method were identical within 0.001. In addition, our method allowed to test all combinations of variables without first selecting one variable among two correlated variables, contrary to the VIF function, which seemed more relevant, so we have kept our variable selection method.

Line 243: I think 4 is quite a low number of variables, and you therefore risk to strongly limit the performance of your model. In SDM, many studies commonly us more than 4 predictors,

so I suggest you don't limit the number of variables and simply remove the ones that are not significant

ANSWER: We understand reviewer's concern, we have constrained the number of variables in the models to a maximum of 4 (ensuring that they were all significant) to avoid excessive complexity of models and difficulty in their interpretation. Although this could limit the performance of our models, we were confident in the procedure parsimony. Indeed, the explained deviances obtained (between 20 and 40%) were high compared to other studies on cetaceans, suggestive of a good fit to the data, and for each model, only four or five variables were actually important, the others representing a very small percentage of the Akaike weights (S3.2 Fig. in Supplementary material 3). As a result, four variable models were the best compromise between avoidance of overfitting, reduction of complexity and maximisation of interpretability. We have added this paragraph lines 420-430 to discuss methodology we have employed.

Line 245: I am very surprised by this result! especially because you consider for each layer both the biomass and production. could you please test with the vif function and see if it is still not correlated?

ANSWER: We have checked the correlations between the variables with our method and the VIF function and we confirm these variables were not correlated (see S3.1 Fig. in Supplementary material 3).

Line 263: have you included the month and year as random effect or explanatory variable then?

ANSWER: We did not include month and year as random effect or explanatory variables in the models because as we discuss lines 408-519, "One of the problems faced when studying the distribution of deep-diving cetaceans through habitat modelling is the lack of sighting data, it is necessary to assemble datasets from different surveys to obtain sufficient data to fit habitat models. Sampling is therefore non-uniform, both spatially and temporally, with some areas, years or seasons being much more represented. For example, some years were only sampled in the northwest Atlantic area. Therefore, we chose to combine all the data to ensure we had enough data without considering a temporal effect in the models. However, temporality was taken into account when associating environmental conditions with effort segments, so when

there was a sighting, it was associated with environmental conditions averaged over the week for the SEAPODYM variables and over the month for the environmental variables before the sighting, to take into account potential time-lags. When deep-diver data will be large enough to allow a more uniform temporal sampling, the temporal aspect will be taken into account in the models.”.

Line 296: to be more logical and consistent with the text I suggest to convert the bathymetry in absolute values the values on both axes are not readable the Y axis should not be in log?

ANSWER: As suggested by reviewer 1, we have changed the scale of the bathymetry in Figure 3. The Y axis is a relative density in individuals/750 km² so it has not to be in log.

Line 302: I still have a big doubt about that. Thanks for sharing the correlation matrix though, I'll have a look at it, but I think running the vif is strongly recommended here

ANSWER: VIF also showed no correlation between the variables as shown in our correlation matrix in S3.1 Fig.

Line 302: when reading Fig 3b it is the opposite, and much more logical indeed! maybe I missed sth here but your graph shows a positive relationship btw bathy biomass and whale's density

ANSWER: Results have changed in the new version of the manuscript because the temporal resolution of SEAPODYM variables have changed so we have modified the text here.

Line 303: however, relationships with epi B and meso B seem very unclear. were these variables significant or you decided to retain 4 variables even if not significant? if you did so, I think this is not the proper way to do it as these 2 predictors seem to have a very little influence on your response variable

ANSWER: All variables selected in the final models were significant but may have less influence compared to other which may have a great influence, we do not believe the method should be questioned here.

Line 304: in your legend you mention 2.5 to 97.5 percentile. Please be consistent

ANSWER: Reviewer 1 is absolutely right, it is a mistake on our part, we have made the necessary changes in the text.

Line 306: I don't really see that on your Fig 4b, probably due to the legend min max values. I suggest you adjust the colorbar to the values of each model, otherwise it is very hard to see any favourable habitat here

ANSWER: We agree the colorbar may not be fully optimised but we chose to apply the same colorbar to facilitate the comparison between the different models, on the advice of many colleagues.

Line 319: I don't understand how your 97.5% percentile can be so tight towards the left-hand side for instance in b) bathy B? it means you have only very few data beyond 4?

ANSWER: We thank reviewer 1 for pointing out this mistake, we have modified the figure.

Line 336: weird because you had no data on such a habitat. likely due to the influence of the slope but it raises serious concern about your model performance to predict outside they are of the training dataset. this needs to be discussed

ANSWER: We understand reviewer's concern and we discuss this on lines 549-557 "We performed a gap analysis to highlight geographical extrapolation areas in which environmental and SEAPODYM variables were out ranges of surveyed conditions. The centre of the study area, near the Mid-Atlantic Ridge, was not surveyed but variable conditions remained within the ranges of surveyed conditions, allowing to predict distribution at a large scale. The gap analysis revealed large gaps in environmental space coverage across the study area, especially in the western part of the Atlantic Ocean for the ENVIRONMENTAL models and in tropical waters for the SEAPODYM models. This suggests that sampling effort was not sufficient in deeper and steeper areas and more intensive sampling effort performed in these areas could help better describe the habitat used by deep- divers."

Line 338: again I disagree with your interpretation here. if you consider the values between the 5 and 95% percentile, the highest densities are found for high values of Bathy B and Epi P, not the opposite

ANSWER: The results have changed here and so the text, we hope our new interpretations agree with reviewer's interpretations.

Line 342: the strong dissimilarities between the ENV and SEAPODYM predictions raise serious concerns regarding the reliability of your modelling approach

ANSWER: The dissimilarities between ENVIRONMENTAL and SEAPODYM predictions mainly raised the effect of the variables and this is likely due to the spatial resolution of the SEAPODYM variables as discussed lines 540-545 "Beaked and sperm whales seemed homogeneously distributed in oceanic waters, which was not very consistent with the sightings and highlighted the poorer performance of the models (higher RMSEs). As previously showed in Lambert et al. (2014), these smoother predictions may be due to the large resolution of the variables. The spatial resolution of the SEAPODYM variables is currently set to 0.25° but is planned to be refined to 0.125° and a finer spatial resolution may reduce this smooth effect.".

Lines 364-368: already said earlier, not necessary here

ANSWER: Text removed.

Lines 373-375: true but such approach could partly explain your relatively low deviances and such differences between your models for each species. For example, sperm whales in the Caribbean might adopt a totally different habitat selection compare to their conspecific in the North-East Atlantic, simply due to genetic differentiation or habitat available

ANSWER: We agree with reviewer 1, deep-divers may have different responses between north and northwest Atlantic and we discussed this on lines 394-407. However, explained deviances were not low compare to other studies that use visual observation data (Becker et al., 2017; Cañadas et al., 2018), which suggested a good fit to the data.

Line 385: another explanation could be related to the spatial resolution of your data. 0.25 degrees is quite coarse to depict specific associations between an animal and its environment. I have heard the SEAPODYM might be soon available daily at 0.08 degrees, so I think it is worth mentioning such limitation

ANSWER: We agree with reviewer 1 and we have added this on lines 443-444.

Line 387: 0.2% difference is very negligible to me so this is not a strong statement. For both species, the SEAPODYM model was less performant, so I don't see the point to include such mid-trophic level covariates

ANSWER: With the change in temporal resolution of the SEAPODYM variable, results have changed and the difference was less negligible but we agree with reviewer 1 that the SEAPODYM models were less performant than ENVIRONMENTAL and COMBINED models.

Line 389: it would be interesting to incorporate more variables into each model and not limit it to 4 to see if it changes the results. if your SEAPODYM covariates appear to be important but less important than the 4 first other variables according to you AIC, it could be the reason why you did not find any SEAPODYM variables in the combined model for beaked whales. not sure it'll change the story but it is worth trying it

ANSWER: We agree with the reviewer's comment but as shown in S3.2 Fig in Supplementary material 3, no SEAPODYM variables were important in the beaked whale COMBINED model and only production of epipelagic organisms was an important variable in the sperm whale COMBINED model so even if the number of variables was higher, SEAPODYM variables probably would not have been selected in the models or they would not have been significant.

Line 393: ok but the slope did not appear to be drive a lot beaked whale distribution neither... probably due to the coarse 0.25 ° resolution you used. A similar study predicting sperm whale distribution based on tracking data faced the same issue with the slope when incorporating it into the model: https://onlinelibrary.wiley.com/doi/full/10.1002/ece3.7154

ANSWER: We do not understand the reviewer's comment here because slope appeared to be an important variable for beaked whales as shown in Fig 3 or in S3.2 Fig in Supplementary material 3.

Line 395: there could also be a temporal mismatch (temporal lag) between SEAPODYM output and the growth rate of the actual prey of these species. This issue was discussed in Chambault et al 2017, DSR

ANSWER: We agree with reviewer's comment and we have added this point on lines 456-457.

Page 17-18:

ANSWER: As suggested by reviewer 1, we have removed repetitions on these two pages and the results have changed in the new version do it is difficult to answer to the specific comments written on these two pages.

Line 449: that's why I suggested to add the bottom temperature as another covariate. such variable was found to play a key role in sperm whale distribution around Mauritius: Chambault et al 2021, Ecol Evol

ANSWER: We fully agree and this will be done in another publication.

Line 451: in theory maybe, but your results show the opposite! also sperm whales can forage on the bottom of the ocean and not necessarily conduct pelagic dives so you need to be careful with your statement: https://onlinelibrary.wiley.com/doi/full/10.1002/ece3.3322

ANSWER: We do not understand why our results show the opposite, the slope was not selected in the COMBINED model (Fig S3) and was not considered as important as shown in S3.2 Fig in Supplementary material, sperm whales are likely to feed at the bottom as well, but perhaps less consistently than beaked whales, which performed deeper dives in average.

Line 453: more than the euphotic depth, you should look at the mixed layer depth as well. for deep divers the light indication given by the Zeu might not be very informative while the MLD

will provide information on the depth of the thermocline which is frequently used by diving animals. this variable can be easily found on Copernicus as well

ANSWER: We agree with reviewer 1; the mixed layer depth would probably be more informative for deep-divers because it provides information on the depth of the thermocline which is frequently used by diving animals and we will explore this in future studies. Here we used euphotic depth because it is a variable available in SEAPODYM but mixed layer depth would probably have been more relevant. We discuss this on lines 515-521.

Line 461: repetition!

ANSWER: Text removed.

Line 467: for animals that display a coastal pattern, like bowhead whales, bathymetry is indeed important. this is a general rule for all coastal animals while pelagic ones will describe more affinities for oceanographic structures like eddies, fronts. a similar relationship was found in narwhals which strongly use glaciers and fjords: https://link.springer.com/article/10.1007/s00300-018-2345-y

ANSWER: We agree with reviewer 1 and it is probably true for sperm whales but according to literature, beaked whales seem particularly linked to bathymetry and physiographic structures like canyons and seamounts.

Line 471: I strongly disagree! the maps were not similar at all between the ENV and SEAPODYM models. You can say that for beaked whales they were quite the same for the ENV and COMBINED models, but this is it

ANSWER: We have changed the text on lines 532-548 and better interpreted our results, we agree, it was poorly worded.

Line 471: I would say totally different and you need to discuss that because it raises serious concern regarding your model performance and reliability. the choice of the algorithm might not be adequate. Machine learning algorithm offer a wide range of algo depicting complex

relationships and might be appropriate and easy to implement on your data. Here, are few exmaples of recent studies involving machine learning in SDM:

Mannocci et al 2021

Chambault et al 2021, Ecol Evol

https://www.sciencedirect.com/science/article/abs/pii/S0304380018304010

https://www.koreascience.or.kr/article/JAKO201216636292901.page

https://link.springer.com/chapter/10.1007/978-1-4419-7390-0_8

ANSWER: We thank reviewer 1 for raising this point. As previously said, machine learning algorithms are justified if the main objective is to predict the distribution of species without necessarily explaining this distribution (Gobeyn et al., 2019) but here our objective was primarily to determine the explanatory performance of models that use different types of explanatory variables. It therefore seemed more relevant to use statistical models rather than machine learning. In addition, surface environmental variables are commonly used in generalized additive models to model the distribution of deep-divers and we wanted to assess whether the addition of prey distribution variables provided additional information to explain their distribution. Therefore, a comparable modelling approach was required. In our future studies, we will consider machine learning algorithms to predict the distribution of species.

Page 20:

ANSWER: All the conclusion has been modified, we hope it will meet the reviewer's expectations.

Reviewer 2 #

Virgili et al. have identified an important gap in our understanding of the link between potential habitat for deep-diving marine predators and the distribution of those predators- namely the influence of prey on the distribution of predators as an intermediate explanatory step between the environment and predators. The most important finding of this work is that the SEAPODYM model of pelagic fish distribution does not explain the distribution of deep-diving predators better than environmental data (except for some weak evidence for sperm whales).

While the science in the paper is basically sound (though see comment below regarding model derivation), the framing needs a bit of work before it should be published. That is, what is the driving hypothesis of this paper? There seem to be two possible frameworks:

1) The current hypothesis is that SEAPODYM variables better explain the distribution of beaked and sperm whales. This was found not to be the case (that is, this is a negative result). If this framework is preferred, it should be stated in the abstract, introduction and conclusion very clearly that SEAPODYM was ruled out as a good framework for describing the distribution of these deep divers

ANSWER: We thank the reviewer 2 for pointing out that the framework was not well enough defined, we fully agree. We have therefore modified the abstract (lines 46-48 " SEAPODYM outputs alone did not seem ideal for modelling prey of deep-diving cetaceans and maybe SEAPODYM was ruled out as a good framework for describing the species distribution, especially for beaked whales."), the introduction (lines 104-109 "We hypothesised that SEAPODYM variables would better explain the distribution of beaked whales and sperm whales because they are more proximal variables and because they characterise deep layers in which deep-divers feed. We also expected a better explanatory power of the model that combined the two types of variables because it considered static variables known to influence the beaked and sperm whale distribution, together with prey distribution data, presumably better suited to describe the species distributions.") and the whole conclusion (lines 577-593) to clarify the framework.

2) Given that, as stated in the discussion lines 396-424, SEAPODYM variables attempt to predict pelagic fish concentrations shallower than 1000m (that is, not the preferred prey of the study predators), a better test of SEAPODYM would be to choose a different predator to examine. Perhaps tuna or some other publicly available dataset (see e.g. https://portal.atn.ioos.us/) could be used to test the model more appropriately?

ANSWER: As stated in the discussion (lines 460-465), deep-divers can feed on prey larger than 20 cm at depths greater than 1,000 m and we agree we reviewer 2 that SEAPODYM variables would probably better explain the distribution of predator species such as tuna (which has been done). However, as stated in the introduction (lines 90-92 “Deep-divers are species of interest because they are sensitive to underwater noise pollution and an accurate knowledge of their distribution is crucial to mitigate the impact of human activities.”) and discussion (lines

374-378 “The models were fitted to sightings of deep-diving cetaceans (beaked and sperm whales) because their habitat use is still poorly known and they face important anthropogenic threats including activities producing high intensity noise (*e.g.* military sonars or seismic guns), which makes them species of major interest.”), it is important to better understand the distribution of deep-divers as their distribution is poorly known and they are species sensitive to underwater noise pollution (*e.g.* active military sonars) so we need to identify new methods that could allow a better understanding of their habitats. The method with the SEAPODYM variables does not seem to be ideal for this according to our study but it allows us to progress in the process.

Besides framing, the other major issue with the manuscript in its current form is that it does not appear that the SEAPODYM model has fundamentally different information than the ENVIRONMENTAL model, thus it is not surprising that it does not improve the explanatory power of the environmental model. That is, in the paragraph on line 160, the SEAPODYM model appears to derive from SST, bathymetric and surface current data, which is the same information that parameterizes the ENVIRONMENTAL model. Is not one then just a transformation of the other? What information does the SEAPODYM model have the that the environmental model does not. There may be something in how data was integrated over time, but it was not clear from the current manuscript how these models are substantially different. In the revision, please clarify how these models provide different information, given that they do not actually include information about prey in the SEAPODYM model.

ANSWER: We fully agree with reviewer 2, the description of the SEAPODYM variables was not complete enough and it was difficult to understand how these variables provide different information from environmental variables. We have therefore modified the section on SEAPODYM variables (lines 187-240) and we hope that the difference is clearer this time. SEAPODYM is a numerical model used to compute the spatial distribution of the biomass and the production of the micronekton and the zooplankton, in different layers of the water column. Energy transfers from the primary production to the groups of micronekton are parameterised in the model and a system of advection–diffusion–reaction equations, that take into account the vertical migrations of organisms, are used to model recruitment, ageing, mortality, and passive transport with horizontal currents. SEAPODYM makes it possible to obtain, in each cell of a grid, an estimate of the total quantity of organisms of each functional group present in each layer and of the productivity of the functional group in each layer.

Minor comments:

Abstract: please include a stronger summary in the abstract describing the main results (that SEAPODYM is not a good predictor of deep-diving cetacean habitat).

ANSWER: The part of the abstract concerning the results has been completely modified to better define the results we have obtained (lines 42-49 "For beaked whales, combining variables did not improve the model performance, no SEAPDOYM variables were selected in the best model, possibly because they prey on organisms less well predicted by SEAPODYM. For sperm whales, by combining variables, we slightly increased the sperm whale model performance (higher explained deviance and lower root mean squared error). SEAPODYM outputs alone did not seem ideal for modelling prey of deep-diving cetaceans and maybe SEAPODYM was ruled out as a good framework for describing the species distribution, especially for beaked whales. However, SEAPODYM gives new insights about potential prey fields targeted by deep-divers at large spatial scales.").

Line 75- also see croll et al. Croll, D. A., Marinovic, B., Benson, S., Chavez, F. P., Black, N., Ternullo, R., & Tershy, B. R. (2005). From wind to whales: trophic links in a coastal upwelling system. Marine Ecology Progress Series, 289, 117-130.

ANSWER: Reference included line 75.

Line 98- not clear what the difference between seapodym and seapodym model are?

ANSWER: 'SEAPODYM model' refers to the generalised additive model fitted to SEAPODYM variables while 'SEAPODYM' refers to the model of Lehodey et al. (2010) that simulated the prey distribution data. We have clarified this on lines 100-102 in the hope that it will be clearer.

Line 166- Why a 29-day sampling window? It seems like this may be appropriate for some variables but for others it would smooth over important information. A sensitivity analysis of choice of sampling window should be considered

ANSWER: We agree with reviewer 2, the 29-day sampling was not appropriate for SEAPODYM variables because the link between species distribution and prey distribution is probably more direct that the one with environmental variables. Consequently, all models were re-fitted with a 7-day temporal resolution for the SEAPODYM variables which was the resolution provided by the data provider.

General point of clarification- the spatial aspect of the modeling seems clear, but it would be helpful to have more explicit discussions of how the temporal aspect of the modeling was done, particularly for predator abundances that shift over time. Was there repeat sampling done or is predator abundance from a single survey? The "gapping" described appears to refer mostly to missing spatial data.

ANSWER: We thank the reviewer for pointing out this lack of clarification in the discussion. A new paragraph has been added to discuss this (lines 408-519 "One of the problems faced when studying the distribution of deep-diving cetaceans through habitat modelling is the lack of sighting data, it is necessary to assemble datasets from different surveys to obtain sufficient data to fit habitat models. Sampling is therefore non-uniform, both spatially and temporally, with some areas, years or seasons being much more represented. For example, some years were only sampled in the northwest Atlantic area. Therefore, we chose to combine all the data to ensure we had enough data without considering a temporal effect in the models. However, temporality was taken into account when associating environmental conditions with effort segments, so when there was a sighting, it was associated with environmental conditions averaged over the week for the SEAPODYM variables and over the month for the environmental variables before the sighting, to take into account potential time-lags. When deep-diver data will be large enough to allow a more uniform temporal sampling, the temporal aspect will be taken into account in the models.").

Reviewer 3 #

The main objective of this paper is to compare habitat models fit to different types of variables to for two deep diving cetaceans: sperm whales and beaked whales. The authors used generalized additive models (GAMs) to compare habitat models fit to 1) environmental variables, 2) simulated prey data from Spatial Ecosystem And POpulation DYnamics Model (SEAPODYM), and 3) combinations of the two variable types. The purpose of this study is to

investigate if more direct measures of prey improve the fit of habitat models, when compared to prey proxies such as surface chlorophyll. The authors predict that more proximal variables, like prey biomass (simulated by SEAPODYM) will improve habitat models by reducing the time lag between phytoplankton biomass and the effect on higher trophic levels. The authors have presented and interesting and relevant study, however there are some issues with the methodology that must be addressed prior to publication. Major and minor concerns are detailed below.

Major Concerns

1. My first concern is within the 'COMBINED' habitat models for both species. The authors stated that they limited the computational burden within the combined model by only included a reduced number of variables; specifically, by only including variables selected within the final "ENVIRONMENTAL" and "SEAPODYM" models. This strikes me as a red flag and limitation. All relevant variables should be included in the 'COMBINED' models. If a variable is not significant in the "ENVIRONMENTAL" model, for example, this does not mean it will automatically not be significant in the "COMBINED" model. Specifically, an interaction between an environmental variable and a SEAPODYM variable could be significant in the "COMBINED" model, and this of course would not show up in the individual "ENVIRONMENTAL" or "SEAPODYM" models.

ANSWER: We understand the point raised by reviewer 3 and understand his concern. We have tried to fit the COMBINED models to all available variables but the computational burden was much too high because the area is very extended and the number of segments is very large (the computer crashed). However, we have revised the method to make it more robust and we hope this will meet the reviewer's expectations. Following Symonds & Moussalli (2011), we have determined the importance of each variable in the ENVIRONMENTAL and SEAPODYM models by summing the Akaike weights of the models in which the variable was selected and ranked all variables. We then included in the selection procedure of the COMBINED models all variables whose percentages of Akaike weight were greater than 25%, as these were the most important variables in the ENVIRONMENTAL and SEAPODYM models. With this method, all possible relevant variables were included in the COMBINED model and we can see in S3.2 Fig (Supplementary material S3) that a large number of variables included in the

selection of the COMBINED model were not important so we think that the method is appropriate to answer our question. The new methodology was included lines 263-271.

2. The authors do not reference/mention spatial auto-correlation or how it was accounted for within the models (e.g. include correlation structure in the model, bin the data at resolutions that are broader than the correlation).

ANSWER: We thank reviewer 3 for pointing this omission. Moran's and Geary's indexes have been calculated to ensure there was no spatial autocorrelation in the data using the 'spdep' R-package. We have added this specification lines 157-158.

3. Explained deviance and AIC are great for model comparison and selection, but are insufficient in terms of model evaluation. AIC and explained deviance are what you use to choose your model, but doesn't tell you anything about model performance. I would like to see an evaluation metric such as area under the curve (AUC) or true skill statistic (TSS). AUC indicates model performance, provide aggregate measures of performance across all possible classification thresholds, and allow performance of models to be compared. TSS measure the discriminatory ability of the SDMs (*e.g.* Becker et al. 2020).

ANSWER: Following reviewer's recommendations, we have included a new parameter for model evaluation in addition to explained deviance which is commonly used to assess model performance (Becker et al., 2017). We have added the Root Mean Squared Error (RMSE) which measures the prediction errors and the model accuracy (the lower, the better; Neill & Hashemi, 2018). We did not used TSS or AUC as proposed by reviewer 3 because these parameters are used to assess the model performance of presence-only or presence-absence models not density models, which are the models we used. In this case, RMSE was more appropriate so we used it and we hope it meet the reviewer's expectations.

4. A lesser concern is with the observations. As I am not a deep-diving whale expert, I am having trouble reconciling the use of surface observation for a study that focuses on fitting habitat models to sub-surface prey simulations. I am not contending that the use of surface observation is inappropriate, however I do think the methods would benefit from more explanation. For example, do we know how surface observation relates to deep dives for prey?

Also including a couple references for studies that have used this type of observation for deep diving species' habitat models previously would be useful.

ANSWER: We understand the reviewer's concern, it may seem inappropriate to use prey simulations at depth to explain the distribution of surface sightings, it would be more appropriate to use tracking data. However, the accepted underlying assumption is that animals observed at the surface are present because they are mostly sensitive to the prey abundance and therefore a high prey biomass at depth could explain the presence of animals at the surface. Brodie et al. (2018) included two subsurface dynamic variables in species distribution models to describe the habitats of four pelagic species in the California Current System and increased the explanatory power and predictive performance of the models for most species. A more systematic use of depth variables could improve the tools available for the planning of human activities, especially for species that would be closely linked to processes at depth. We have added a part in the discussion about this, lines 383-393.

Minor comments

Line 57-59: "cetacean distributions are barely known" is an overgeneralization. If you are referring to deep-diving cetaceans, this needs to be made more explicit. Distributions of some cetaceans are actually quite well known and well modeled (e.g. Abrahms et al. 2019 – blue whales).

ANSWER: We agree with reviewer 3, we have specified "the deep-diving cetacean distributions" line 59.

Lines 291-294; 307, etc.: The way the authors refer to the "COMBINED" model for the beaked whale is confusing. The authors should state that the "COMBINED" model is identical to the "ENVIRONMENTAL" model at the beginning of the results section referencing beaked whales. Stating the comparison of AIC scores prior to making this point is confusing and makes it seem like these are different models (i.e. contain different variables). Similarly, within the Figure 3, it seems unnecessary to show identical maps for 3a and 3c. Instead, the authors should simply indicate that the "COMBINED" model is identical, and not in fact a separate model with separate results.

ANSWER: We have followed reviewer's recommendation, we have simplified the text (lines 305-325) and we have removed the plot and the map referring to the beaked whale COMBINED model in figures 3 and 4.

Line 333-334, etc.: Remove line "All selected variables were poorly correlated" lines from the results section. This is inherent based on the methodology. As such, you would only need to report if variables were slightly correlated, e.g.

ANSWER: Done.

Line 418-424: I did find myself wondering the need to combine observation across such a large region. The authors offer an explanation in lines 418-424, but I don't think this explanation is sufficient. Why not focus this study on one of the regions referenced? Would the authors expect prey data to be more relevant and improve fit at regionally specific scales? Is it possible that such a broad spatial area masks the impacts of prey data?

ANSWER: We thank reviewer 3 for raising this point. One of the problems faced when studying the distribution of deep-diving cetaceans through habitat modelling is the lack of sighting data, it is necessary to assemble datasets from different surveys and different areas to obtain sufficient data to fit habitat models. The combination of sighting data from different ecosystems (*e.g.* North-East and North-West Atlantic) may mask inter-regional differences in the relationships between cetacean densities and the environmental predictors and may mask the influence of prey data on species distribution. The objective here, was to compare models using environmental variables, prey distribution variables, and a combination of the two types of variables to determine the extent to which the models could be improved and not necessarily explain precisely the mechanisms influencing the distribution of beaked and sperm whales, which would be more consistent at the scale of a smaller region. We built basin-wide models and did not investigate region-specific models in order to leverage large sample sizes for investigating the explanatory power of SEAPODYM variables for habitat use of deep-divers. We acknowledge that pooling regions across an oceanic basin may introduce bias, but this study is a first investigation, and appreciate region-specific models would represent an obvious improvement (once enough data are available at the regional scale). We also think that in a global conservation context, it is important to study the broad scale cetacean distribution to obtain a more global prediction of this distribution and limit anthropogenic threats. We have

added a paragraph to discuss this point lines 494-407. We thus agree with reviewer 3 it would be better to focus on one region, which is what we will intend to do in our further studies.

Lines 483-484: Generally, in this part of the discussion, the authors continue to refer to the "COMBINED" models (plural) limiting the uncertainty within the interpolation. However, the authors previously state that the "COMBINED" model is identical to the "ENVIRONMENTAL" model for beaked whales, meaning that these conclusions would not be true for the beaked whales. The authors need to specifically reference the sperm whale example when making these statements/conclusions.

ANSWER: We agree with reviewer 3, we have changed the text through the whole discussion.

References

Becker E.A, Forney K.A, Thayre B.J, Debich A.J, Campbell G.S, Whitaker K, et al. (2017). Habitat-based density models for three cetacean species off Southern California illustrate pronounced seasonal differences. Frontiers in Marine Science, 4:121.

Brodie S, Jacox M.G, Bograd S.J, Welch H, Dewar H, Scales K.L, et al. (2018). Integrating dynamic subsurface habitat metrics into species distribution models. Frontiers in Marine Science, 5: 219.

Cañadas A, de Soto N.A, Aissi M, Arcangeli A, Azzolin M, B-Nagy A., et al. 2018. The challenge of habitat modelling for threatened low density species using heterogeneous data: The case of Cuvier's beaked whales in the Mediterranean. Ecological Indicators, 85: 128–136.

Gobeyn S, Mouton A.M, Cord A.F, Kaim A, Volk M, Goethals P.L. (2019). Evolutionary algorithms for species distribution modelling: A review in the context of machine learning. Ecological modelling, 392: 179-195.

Lambert C, Mannocci L, Lehodey P, Ridoux V. 2014. Predicting cetacean habitats from their energetic needs and the distribution of their prey in two contrasted tropical regions. PloS one, 9(8): e105958.

Lehodey P, Murtugudde R, Senina I. 2010. Bridging the gap from ocean models to population dynamics of large marine predators: a model of mid-trophic functional groups. Progress in Oceanography, 84(1): 69-84.

Neill S.P, & Hashemi M.R. (2018). Fundamentals of ocean renewable energy: generating electricity from the sea. Academic Press, pp 193-235.

Roberts J.J, Best B.D, Mannocci L, Fujioka E, Halpin P.N, Palka D.L, et al. 2016. Habitat-based cetacean density models for the U.S. Atlantic and Gulf of Mexico. Scientific Report, 6.

Symonds M.R, & Moussalli A. (2011). A brief guide to model selection, multimodel inference and model averaging in behavioural ecology using Akaike's information criterion. Behavioral Ecology and Sociobiology, 65(1): 13-21.

Virgili A, Authier M, Boisseau O, Cañadas A, Claridge D, Cole T, et al. 2019. Combining multiple visual surveys to model the habitat of deep- diving cetaceans at the basin scale. Global Ecology and Biogeography, 28(3): 300-314.
