# Supplementary material for: Towards a better characterisation of deep-diving whales’ distributions by using prey distribution model outputs?
Source: PLoS One. 2021 Aug 4;16(8):e0255667. doi: 10.1371/journal.pone.0255667 (PMC8336804; doi:10.1371/journal.pone.0255667)
Supplement: Attachment — Submitted filename: RebuttalLetter.docx [file pone.0255667.s008.docx]

Auriane Virgili

La Rochelle University, France

auriane.virgili@univ-lr.fr

Dear Editor,

Thank you for considering our article entitled “Towards a better characterisation of deep-diving whales’ distributions by using prey distribution model outputs?”, for publication in the journal PLOS ONE. We have taken into account all comments from the editor and the reviewers regarding the article.

We thank the reviewers for their careful reading of this new version of the manuscript and for pointing out elements in the abstract, methods and discussion that needed to be more clarified. We understand reviewer's comments about the way we run species distribution models and we believe we have clearly developed the limitations associated with our methodology in the discussion. The use of bottom variables is a very interesting proposal, especially for deep divers, and we have therefore mentioned it in the discussion. These variables are the subject of recent development and require further investigation before they can be used. This work is currently in project in our team and we feel is outside the scope of this study. The treatment of bottom variables or variables integrated in the water column is more complex than that of surface variables and it can also be criticised to use bottom variables to explain sightings recorded at the surface. The limitations associated with the use of bottom variables would be added to the limitations described in our study, and we believe it was better to separate the two approaches for greater understanding. We also understand the reviewer's caveats about limiting the number of variables in our models. Indeed, limiting the number of variables when your aim is solely to predict may be detrimental (but not necessarily, *e.g.* Authier et al. 2017), but for explanation it is not (*e.g.* Achen 2002, Shmuéli, 2010). Considering many variables in tandem will inevitably pose interpretation challenges, and may also lead to estimation problems of each individual effects because the information in the data is being spread thin on estimating a large number of parameters (see below for further explanation). We agree with the reviewer that an improvement in the models of only 0.8 was not sufficient and therefore we have modified the conclusions of our study rejecting our initial hypothesis. We hope this will meet the reviewer’s expectations.

We have fixed all typos and grammatical mistakes during the revision and ensured that the manuscript meets the PLOS ONE’s style requirements.

We hope that the revised manuscript addresses the different concerns raised by the editor and the reviewers, whom we thank for their careful reading and review of this manuscript. We feel the revised manuscript has improved as a result and hope it will be deemed acceptable for consideration of publication in PLOS ONE.

Sincerely,

On behalf of all co-authors,

Auriane Virgili

Concerning specific reviewer’s comments:

**Reviewer 1 #**

**Specific comments in the manuscript:**

Line 45: need reformulation

**ANSWER:** We have reformulated the sentence lines 43-45 “For beaked whales, no SEAPODYM variable was selected in the best model, possibly because SEAPODYM does not accurately simulate the organisms on which beaked whales feed on”.

Line 49: I'm wondering how seapodym could be ideal to predict the whale's distribution if it is "not ideal for modelling prey of deep-diving cetaceans"? your sentence needs to be rephrased to be consistent with your results.

**ANSWER:** We have rephrased the sentence lines 46-47 “SEAPODYM outputs were at best weakly correlated with sightings of deep-diving cetaceans, suggesting SEAPODYM may not accurately predict the prey fields of these taxa”.

Line 322: is it why you have one panel missing for BW? you mean your smooth curves and explained deviances were exactly the same for the ENV and COMBINED models? surprising...

**ANSWER:** As suggested by reviewer 3 in the previous version of the manuscript, we have pooled the results of the ENVIRONMENTAL and CONBINED models in the figures because the models were identical (same variables selected).

Line 329: > 15°C seems already to be associated with higher densities, right?

**ANSWER:** That is right, we have changed the result.

Lines 376, 384: again I found these SEAPODYM maps hard to interpret using the same colorbar as the 2 other models. I understand your concern of being able to compare the 3 models, but why don't you put the scale into log to have a better contrast? again hard to see from your maps

**ANSWER:** We have used a square root scale to have a better contrast because a log scale is not appropriate for low values. We hope it is better now.

Line 454: again I found this argument very questionable, especially when using SDMs

**ANSWER:** Limiting the number of variables when your aim is solely to predict may be detrimental (but not necessarily, *e.g.* Authier et al., 2017), but for explanation it is not (see *e.g.* Achen, 2002). See Shmuéli (2010) for an indepth treatment of the difference between predictive modelling and explanatory modelling. Considering many variables in tandem will inevitably pose interpretation challenges, and may also lead to estimation problems of each individual effects (not to mention interactions) because the information in the data is being spread thin on estimating a large number of parameters. Another problem is the inevitable “all else being equal” assumption which result when individual effects are presented. These effects are conditional on all other variables in the model being set to their average values. Yet even slight covariations between variations makes this interpretation brittle as when one variable change, so do all the others (there by violating the underlying assumption of all-else-being-equal). One solution may be to present average predictive comparisons (Gelman & Pardoe, 2007) but these are more involved in their computations and not very much used (although they would be more accurate as they are marginal estimates, integrating over the other covariates in a model). The problem of having many variables in an explanatory model is that the geometry of the parameters space quickly become unwieldy and it is increasingly difficult to understand this geometry and interpret these parameters well. We are thus of the mind that for explanatory purposes, an appropriate strategy is to restrict the complexity. This may not be warranted when the goal is purely predictive, in which case interpretation is not sought either. In general, we should not expect a good predictive model to be also a good explanatory one (Shmuéli 2010, Smith & Santos, 2020). In the grey zone, a safety net is needed, and we have found the exposition in Achen (2002) to be quite persuasive. Out motivation for limiting the complexity to a maximum of 4 covariates can be traced back to ‘ART’: A Rule of Three. A statistical specification with more than three explanatory variables is meaningless (Achen, 2002, page 446). This seemingly outrageous statement is motivated by the empirical observation (which in our own experience is correct) that ‘With more than three independent variables, no one can do the careful data analysis to ensure that the model specification is accurate and that the assumptions fit as well as the researcher claims’ (Achen, 2002).

Line 643: I still found this sentence unclear and contradictory

**ANSWER:** We have rephrased the sentence lines 592-593 “SEAPODYM did not seem to model accurately the prey of deep-diving cetaceans assuming the later are sighted at the surface of their foraging grounds”.

Line 644: by 0.8 !! this result is highly negligible so I won't mention it

**ANSWER:** We agree with the reviewer, we have changed the sentence lines 593-595 “By combining SEAPODYM and environmental variables the increase model performance for sperm whale was only marginal”.

Line 655: given your main findings I'm not sure SEAPODYM will be very helpful to predict the distribution of such species

**ANSWER:** We agree with the reviewer, we have changed the sentence lines 602-605 “A more systematic use of SEAPODYM could allow to better define the limits of its use and a development of the model that would simulate larger prey beyond 1,000 m would probably better characterise the prey of deep-diving cetaceans”.

**Specific comments in the rebuttal letter:**

Here we rewrite the response we have made in the rebuttal letter, we add the reviewer's comment about that response and we respond to the comment.

Rebuttal letter: We do not believe that it makes sense to use the same data source for the environmental and SEAPODYM variables as this will necessarily lead to a correlation between the variables and therefore we would not be able to obtain a combination of the environmental and SEAPODYM variables in the COMBINED models.

Reviewer’s comment: I disagree. Nowadays there are plenty of ocean products available from different databases and these can differ from one another. If SEAPODYM assimilates sst and currents data from one source, it does not necessarily mean that these 2 variables will be strongly correlated to your COMBINED model because complex calculations are done to produce mid-trophic level outputs. But if you're more familiar with AVISO products fine, you should simply say so because your justification to use such products is not valid

**ANSWER:** We are indeed more familiar with AVISO products, we use them routinely and the possibilities offered by AVISO are very large. However, it would be possible that the variables used to simulate SEAPODYM variables would be correlated with our variables if we used the same source, we did not have the opportunity to test this.

Rebuttal letter: We thank reviewer 1 for this information but we do not think this type of variable at a very fine spatial resolution is very relevant for visual observation data, it would be more relevant for tracking data and therefore would not be applicable here.

Reviewer’s comment: The eddy atlas from AVISO is not at a finer resolution as the other products you are using since the eddies are estimated from SLA and possibly from ARGO floats, so it is not a matter of spatial resolution. Would be interesting to see if your whales select some particular eddy types, just a suggestion. At least you could add few lines to the Discussion?

**ANSWER:** We agree with the reviewer, we have added few lines to mention that lines 524-526 “It would be particularly interesting to characterise eddies (*e.g.* cyclonic or anticyclonic) to assess whether deep-divers preferentially select a certain type of eddy as it would appear that prey aggregating at eddies are different depending on their rotation cycles [78]”.

Rebuttal letter: We fully agree with reviewer 1 and the use of depth variables will be the subject of another publication. Here, the objective was to compare the SEAPODYM variables to environmental variables classically used in habitat models such as static variables and surface variables that was why we did not include bottom temperature in the models.

Reviewer’s comment: it is a pity you exclude so many variables of interest, especially for deep divers. Bottom temperature has been used in a recent publication on sperm whales (Chambault et al 2021, Eco Evol) and I don't believe it will be that complicated to rerun your models with this additional variable

**ANSWER:** As mentioned above, the use of bottom variables is very interesting, especially for deep divers, and we have therefore mentioned it in the discussion lines 396-402 and 520-526. These variables are the subject of recent development and require further investigation before they can be used. This work is currently in project in our team and we feel is outside the scope of this study. The treatment of bottom variables or variables integrated in the water column is more complex than that of surface variables and it can also be criticised to use bottom variables to explain sightings recorded at the surface. The limitations associated with the use of bottom variables would be added to the limitations described in our study, and we believe it was better to separate the two approaches for greater understanding. Contrary to what the reviewer thinks, it is tedious and very time-consuming to re-run the models with other variables because it is necessary to extract the variables over 18 years, to associate the variables with the effort segments (more than 244,000 segments) and to re-run the models, which requires several days per model, and even more if variables are added. This will therefore be the subject of another study.

Rebuttal letter: The dissimilarities between ENVIRONMENTAL and SEAPODYM predictions mainly raised the effect of the variables and this is likely due to the spatial resolution of the SEAPODYM variables as discussed lines 540-545 “Beaked and sperm whales seemed homogeneously distributed in oceanic waters, which was not very consistent with the sightings and highlighted the poorer performance of the models (higher RMSEs). As previously showed in Lambert et al. (2014), these smoother predictions may be due to the large resolution of the variables. The spatial resolution of the SEAPODYM variables is currently set to 0.25° but is planned to be refined to 0.125° and a finer spatial resolution may reduce this smooth effect.”.

Reviewer’s comment: 0.08 degrees daily and not 0.125°. Not convinced this has anything to do with the spatial resolution but more of the lack of sightings and/or not inappropriate covariates. I bet if you would conduct the same analysis with the new resolution of SEAPODYM the results would be identical, meaning the differences are not due to the spatial resolution but rather to the covariates themselves

**ANSWER:** We thank the reviewer for pointing out this mistake, we have changed the value in the text. We agree that SEAPODYM variables are certainly not suitable for deep divers as SEAPODYM does not simulate large enough prey and deep enough layers, however a finer resolution of the variables could perhaps allow for more structured predictions and not obtaining homogeneous predictions. It could also be due to the lack of data as suggested by the reviewer but in this case, we would have observed the same problem on the maps of the ENVIRONMENTAL and COMBINED models.

Rebuttal letter: We agree with the reviewer’s comment but as shown in S3.2 Fig in Supplementary material 3, no SEAPODYM variables were important in the beaked whale COMBINED model and only production of epipelagic organisms was an important variable in the sperm whale COMBINED model so even if the number of variables was higher, SEAPODYM variables probably would not have been selected in the models or they would not have been significant.

Reviewer’s comment: in your Table SI 31, I don't understand your last column "delta AIC". It does not match the AIC column so please explain. In the 4th and 5th rows of the combined models, there ARE seapodym layers,and the AIC are very similar to teh best model so I'm not really convinced by your answer here

**ANSWER:** There was an error in the Appendix due to rounding, the correction has been made. However, this does not change the results. We refer here to figure S3.2 which ranks the variables according to their Akaike weights and we can see that in the COMBINED beaked whale model there are only 4 variables that stand out as important and no SEAPODYM variables and for sperm whales 5 variables stand out with only epipelagic production for the SEAPODYM variables so including additional variables would probably not change anything in the model results.

Rebuttal letter: We do not understand why our results show the opposite, the slope was not selected in the COMBINED model (Fig S3) and was not considered as important as shown in S3.2 Fig in Supplementary material, sperm whales are likely to feed at the bottom as well, but perhaps less consistently than beaked whales, which performed deeper dives in average.

Reviewer’s comment: your results (at least the previous version) showed no improvement when incorporation seapodym variables, suggesting the ENV model was more performant, that's why to me your results show the opposite

**ANSWER:** The reviewer's comment is still unclear, as slope does not appear to be an important variable for sperm whales unlike beaked whales, as shown in Figure S3.2, we can reasonably assume that they are less slope dependent than beaked whales.

Rebuttal letter: We agree with reviewer 1; the mixed layer depth would probably be more informative for deep-divers because it provides information on the depth of the thermocline which is frequently used by diving animals and we will explore this in future studies. Here we used euphotic depth because it is a variable available in SEAPODYM but mixed layer depth would probably have been more relevant. We discuss this on lines 515-521.

Reviewer’s comment: rather than justifying in the text why you have not included this variable, it would have been much more robust and convincing to actually include it! The fact that Zeu is available from SEAPODYM is not a convincing justification since the MLD is easily available from CMEMS website and can be incorporated in your models the same way your you did with the seapodym variables

**ANSWER:** We agree with the reviewer but as mentioned above, it takes a long time to add a variable and the objective here was not to test all the variables available for deep divers but to compare with the SEAPODYM variables so indeed it would have been preferable to add the MLD but it was not a priority for the study, it was more meaningful to focus on the temporality of the variables at 7 days.

Rebuttal letter: We thank reviewer 1 for raising this point. As previously said, machine learning algorithms are justified if the main objective is to predict the distribution of species without necessarily explaining this distribution (Gobeyn et al., 2019) but here our objective was primarily to determine the explanatory performance of models that use different types of explanatory variables. It therefore seemed more relevant to use statistical models rather than machine learning. In addition, surface environmental variables are commonly used in generalized additive models to model the distribution of deep-divers and we wanted to assess whether the addition of prey distribution variables provided additional information to explain their distribution. Therefore, a comparable modelling approach was required. In our future studies, we will consider machine learning algorithms to predict the distribution of species.

Reviewer’s comment: I agree if your scope is also to explain the relationships then the GAMs or GLMs are a better choice than ML. However, the justification of keeping surface variables only because this is the "common way" to do it is not scientifically appropriate

**ANSWER:** As mentioned above, we plan to use bottom variables in habitat models but these variables are the subject of recent development and require further investigation before they can be used. We do not consider that keeping only surface variables because they are commonly used is the most appropriate but it is a fact that many studies on deep divers use surface variables in habitat modelling (*e.g.* Roberts et al., 2016; Becker et al., 2017; Cañadas et al., 2018), the use of bottom variables is very recent. Our objective was therefore to propose new variables, the SEAPODYM variables, which are integrated in the water column and could have been appropriate variables for deep divers. It turned out that they were not and for this reason new work was undertaken to use other variables that would be more suitable, so this is another study and mixing the two approaches would have complicated the message considerably.

**References**

Achen, C. H. (2002). Toward a new political methodology: Microfoundations and ART. Annual review of political science, 5(1), 423-450.

Authier, M., Saraux, C., & Péron, C. (2017). Variable selection and accurate predictions in habitat modelling: a shrinkage approach. Ecography, 40(4), 549-560.

Becker E.A, Forney K.A, Thayre B.J, Debich A.J, Campbell G.S, Whitaker K, et al. (2017). Habitat-based density models for three cetacean species off Southern California illustrate pronounced seasonal differences. Frontiers in Marine Science, 4:121.

Cañadas A, de Soto N.A, Aissi M, Arcangeli A, Azzolin M, B-Nagy A., et al. 2018. The challenge of habitat modelling for threatened low density species using heterogeneous data: The case of Cuvier’s beaked whales in the Mediterranean. Ecological Indicators, 85: 128–136.

Gelman, A., & Pardoe, I. (2007). 2. Average predictive comparisons for models with nonlinearity, interactions, and variance components. Sociological Methodology, 37(1), 23-51.

Roberts J.J, Best B.D, Mannocci L, Fujioka E, Halpin P.N, Palka D.L, et al. 2016. Habitat-based cetacean density models for the U.S. Atlantic and Gulf of Mexico. Scientific Report, 6.

Shmueli, G. (2010). To explain or to predict? Statistical science, 25(3), 289-310.

Smith, A. B., & Santos, M. J. (2020). Testing the ability of species distribution models to infer variable importance. Ecography, 43(12), 1801-1813.
